# Pilot testing the EARS-Vet surveillance network for antibiotic resistance in bacterial pathogens from animals in the EU/EEA

**DOI:** 10.3389/fmicb.2023.1188423

**Published:** 2023-05-22

**Authors:** Justine Lagrange, Jean-Philippe Amat, Cristina Ballesteros, Peter Damborg, Thomas Grönthal, Marisa Haenni, Eric Jouy, Heike Kaspar, Kevin Kenny, Babette Klein, Agnese Lupo, Jean-Yves Madec, Charlotte Mark Salomonsen, Elisabeth Müller, Cristina Muñoz Madero, Oskar Nilsson, Madelaine Norström, Suvi Nykäsenoja, Gudrun Overesch, Karl Pedersen, Tarja Pohjanvirta, Rosemarie Slowey, Cristiana Teixeira Justo, Anne Margrete Urdahl, Christos Zafeiridis, Eric Zini, Géraldine Cazeau, Nathalie Jarrige, Lucie Collineau

**Affiliations:** ^1^Laboratory of Lyon, Epidemiology and Surveillance Support Unit, French Agency for Food, Environmental and Occupational Health & Safety (ANSES), University of Lyon, Lyon, France; ^2^Claude Bernard University of Lyon 1, Lyon, France; ^3^Agencia Española de Medicamentos y Productos Sanitarios, Madrid, Spain; ^4^Department of Veterinary and Animal Sciences, University of Copenhagen, Frederiksberg, Denmark; ^5^Finnish Food Authority, Helsinki, Finland; ^6^Laboratory of Lyon, Antimicrobial Resistance and Bacterial Virulence Unit, French Agency for Food, Environmental and Occupational Health & Safety (ANSES), University of Lyon, Lyon, France; ^7^Laboratory of Ploufragan-Plouzané-Niort, Mycoplasmology, Bacteriology and Antimicrobial Resistance Unit, French Agency for Food, Environmental and Occupational Health & Safety (ANSES), Ploufragan, France; ^8^Federal Office of Consumer Protection and Food Safety, Berlin, Germany; ^9^Department of Agriculture, Food and the Marine Laboratories, Celbridge, Ireland; ^10^Laboklin GmbH & Co. KG, Bad Kissingen, Germany; ^11^Veterinary Laboratory, Danish Agriculture & Food Council F.m.b.A., Copenhagen, Denmark; ^12^National Veterinary Institute of Sweden, Uppsala, Sweden; ^13^Norwegian Veterinary Institute, Ås, Norway; ^14^Institute of Veterinary Bacteriology, Vetsuisse Faculty, University of Bern, Bern, Switzerland; ^15^Seconded National Expert to the European Commission (DG Health and Food Safety), Ministry of Rural Development and Food of Greece, General Directorate of Veterinary Services, Athens, Greece; ^16^AniCura Istituto Veterinario Novara, Granozzo con Monticello, Italy; ^17^Vetsuisse Faculty, Clinic for Small Animal Internal Medicine, Zurich, Switzerland; ^18^Department of Animal Medicine, Production and Health, University of Padova, Padua, Italy

**Keywords:** antimicrobial resistance, veterinary clinical pathogens, monitoring, integrated surveillance, One Health

## Abstract

**Introduction:**

As part of the EU Joint Action on Antimicrobial Resistance (AMR) and Healthcare-Associated Infections, an initiative has been launched to build the European AMR Surveillance network in veterinary medicine (EARS-Vet). So far, activities included mapping national systems for AMR surveillance in animal bacterial pathogens, and defining the EARS-Vet objectives, scope, and standards. Drawing on these milestones, this study aimed to pilot test EARS-Vet surveillance, namely to (i) assess available data, (ii) perform cross-country analyses, and (iii) identify potential challenges and develop recommendations to improve future data collection and analysis.

**Methods:**

Eleven partners from nine EU/EEA countries participated and shared available data for the period 2016–2020, representing a total of 140,110 bacterial isolates and 1,302,389 entries (isolate-antibiotic agent combinations).

**Results:**

Collected data were highly diverse and fragmented. Using a standardized approach and interpretation with epidemiological cut-offs, we were able to jointly analyze AMR trends of 53 combinations of animal host-bacteria–antibiotic categories of interest to EARS-Vet. This work demonstrated substantial variations of resistance levels, both among and within countries (e.g., between animal host species).

**Discussion:**

Key issues at this stage include the lack of harmonization of antimicrobial susceptibility testing methods used in European surveillance systems and veterinary diagnostic laboratories, the absence of interpretation criteria for many bacteria–antibiotic combinations of interest, and the lack of data from a lot of EU/EEA countries where little or even surveillance currently exists. Still, this pilot study provides a proof-of-concept of what EARS-Vet can achieve. Results form an important basis to shape future systematic data collection and analysis.

## Introduction

Antimicrobial resistance (AMR) has been widely recognized as a major public health problem, responsible for an estimated 23,100 human deaths every year in Western and Central Europe (Murray et al., 2022). To address this issue, a One Health surveillance approach is needed, as stated in the European Union (EU) One Health Action Plan against AMR (European Commission, 2017). In the human sector, the European Centre for Disease Prevention and Control (ECDC) coordinates the European Antimicrobial Resistance Surveillance Network (EARS-Net), which monitors AMR in bacteria isolated from invasive infections in blood and cerebrospinal fluid in hospitalized patients (European Centre for Disease Prevention and Control and World Health Organization, 2022). Furthermore, the European Food- and Waterborne Diseases and Zoonoses Network (FWD-Net) monitors AMR in *Salmonella* and *Campylobacter* from human infections (European Centre for Disease Prevention and Control, 2022).

In the animal and food sector, the European Food Safety Authority (EFSA) coordinates a mandatory active monitoring of AMR in zoonotic (*Salmonella* and *Campylobacter*), indicator bacteria (*Escherichia coli*) and extended-spectrum-cephalosporin-resistant and carbapenemase-producing *E. coli* from healthy food-producing animals (cattle, poultry, pigs) at slaughter and meat thereof, according to Directive 2003/99/EC (European Commission, n.d.) and Decision 2020/1729/EU (EU, n.d.). While the majority of AMR data in the human sector originate from diseased individuals, the existing European surveillance programs lack AMR data in pathogens from diseased animals. Thus, information is missing to guide antimicrobial stewardship initiatives such as treatment guidelines, and to guide policymakers in regulating veterinary antibiotic use, toward the shared goal of reducing AMR while ensuring optimal treatment of animal infections (Mader et al., 2021). Hence, the lack of a coordinated program on surveillance of AMR in bacterial pathogens of animals is an important gap in the current European AMR surveillance strategy.

As part of the EU Joint Action on AMR and Healthcare-Associated Infections (EU-JAMRAI), an initiative was launched in 2017 to build the European Antimicrobial Resistance Surveillance network in veterinary medicine (EARS-Vet) (Mader et al., 2021). An initial review of existing national monitoring systems for AMR in animal bacterial pathogens across Europe showed that in 2020, 12 out of 27 EU/EEA countries had at least one national monitoring system in place (15 programs in total), although with highly diverse structures and operations, including diverse laboratory methods and standards (Mader et al., 2022b).

Following this review, a group of experts from 14 EU/EEA countries, the majority of which were actively involved in the 15 identified programs, joined forces to establish a methodological basis for EARS-Vet, including the EARS-Vet objectives, standards (i.e., antimicrobial susceptibility testing (AST) methods and interpretation criteria), and scope (i.e., the combinations of animal host–bacterial species–antibiotics) (Mader et al., 2021, 2022a). Paralleling EARS-Net in the human sector, EARS-Vet aims to describe the AMR situation, follow AMR trends and detect emerging AMR in bacteria from diseased animals in Europe, in order to advise policy, evaluate interventions, and support antimicrobial stewardship in veterinary medicine, among other potential applications (Mader et al., 2021). Tentative EARS-Vet standards and scope were defined by consensus and following a bottom-up approach, i.e., considering the surveillance activities already performed in the majority of participating countries (Mader et al., 2022a). The proposed scope included combinations of six animal host (cattle, swine, chickens, turkeys, cats, and dogs) and 11 bacterial species (Mader et al., 2022a).

Drawing on the EARS-Vet milestones achieved so far, the objective of this study was to pilot test surveillance by EARS-Vet. Specifically, we aimed to (i) assess available data from partner countries, (ii) perform cross-country analyses for selected bacteria–antibiotic combinations, (iii) identify potential challenges, and (iv) formulate recommendations for future improvement of EARS-Vet data collection and analysis.

## Materials and methods

### Data sources

Upon signature of a data sharing agreement, 11 partners from nine European countries participated in the EARS-Vet pilot study and shared their available data for the years 2016 to 2020. The majority of partners retrieved data from national monitoring systems of AMR in bacterial isolates from diseased animals, the structures and operations of which have been described previously (Mader et al., 2022b). Additional data on companion animals were retrieved in Germany from LABOKLIN GmbH & Co KG, a large private veterinary diagnostic laboratory providing services in the whole country, and in Italy from a large veterinary referral hospital (AniCura Istituto Veterinario Novara) receiving animal patients from Northern Italy and subcontracting bacteriology testing to a private clinical laboratory (Idexx). A brief description of data providers is available in Table 1.

**Table 1:** Description of data providers.

Country	Partner or program	Animal species covered	Number of contributing laboratories	Geographical coverage	Reference
Denmark	UCPH^1^	Cat, dog	1	National	-
Denmark	Laboratory for Pig Diseases^2^	Cattle, swine	1	National	-
Germany	LABOKLIN	Cat, dog	1	National	-
Germany	GE*RM*-Vet	Cat, cattle, dog, swine	2	National	Bundesamt für Verbraucherschutz und Lebensmittelsicherheit (2022)
Finland	FINRES-Vet	Cattle, Chicken, Swine, Turkey	2	national	Finnish Food Authority (2020)
France	RESAPATH	Cat, cattle, Chicken, Dog, swine, turkey	71	national	ANSES (2021)
Italy	AniCura^3^	Cat, dog	1	Regional (Northern Italy)	-
Norway	NORM-VET	Cat, cattle, chicken, dog, swine, turkey	1	National	NORM/NORM-VET 2020 (2021)
Spain	SEVAE^4^	Cattle, swine	22	Regional (North-eastern Spain)	Plan Nacional Resistencia Antibióticos (2022)
Sweden	SVA^5^	Cat, cattle, chicken, dog, swine, turkey	1	National	Swedres-Svarm (2021)
Switzerland	ZOBA^6^	Cat, cattle, chicken, dog, swine	1	National	Federal Office of Public Health and Federal Food Safety and Veterinary Office (2022)

^1^University of Copenhagen, Denmark. ^2^Danish Agriculture and Food Council, Denmark. ^3^AniCura Istituto Veterinario Novara, Italy. ^4^Sistema Español de Vigilancia de Animales Enfermos, Spain. ^5^National Veterinary institute, Sweden. ^6^Institute of Veterinary Bacteriology, University of Bern, Switzerland. Additional details on each program are available in Mader et al. (2022b).

### Laboratory techniques

**Figure 1:**
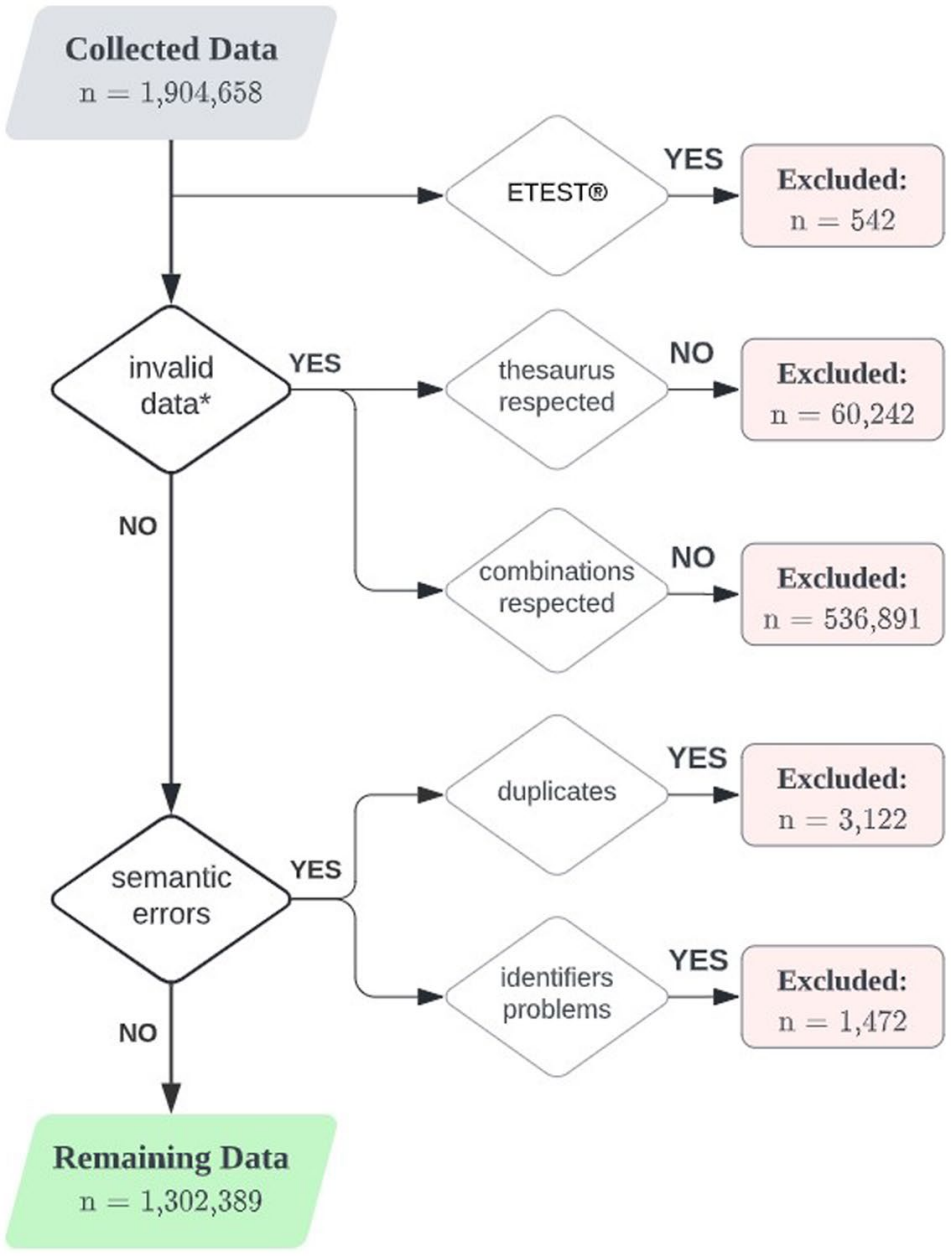
Flow diagram depicting the data trimming process. n = number of entries, i.e., combinations of isolate ID–antibiotic agent tested for+ *Data were considered invalid in case they were out of the EARS-Vet scope previously defined by Mader et al. (2022a).

Bacterial identification was performed using MALDI-TOF (all 11 partners) and biochemical tests (API galleries, bioMérieux, two out of 11 partners, Supplementary Table S1). Nine partners provided broth microdilution data, whereas RESAPATH provided disk diffusion data only and SEVAE a combination of broth microdilution, disk diffusion, and antibiotic gradient strips (i.e., ETEST^®^) data (Supplementary Table S1). Due to the limited number of isolates and concerns about data comparability, ETEST^®^ data were excluded from further analysis (Figure 1). Aside from AniCura and SEVAE (prior to 2019), both of which used VITEK 2^®^ technology (bioMérieux), and Laboklin which used MERLIN Diagnostika GmbH, partners who used broth microdilution all used commercial microtiter plates (either VetMIC before production ceased in 2018 or Thermo Fisher Scientific Inc.). Eight partners referred to methodological standards of the Clinical and Laboratory Standards Institute [CLSI, VET01 standards (CLSI, 2018)]. NORM-VET and SVA used the norm ISO 20776-1:2019 as recommended by the European Committee on Antimicrobial Susceptibility Testing (EUCAST). RESAPATH used the AFNOR standard NF U 47–107 associated to the guidelines of the French society for microbiology (CA-SFM Vet) antibiogram committee (veterinary group). Virulence testing of swine *E. coli* isolates was performed using PCR (SVA and ZOBA), hemolysis testing (Laboratory for Pig Diseases), or a combination of PCR and hemolysis testing (FINRES-Vet).

### Data collection

A data collection template was circulated to all partners in the form of a Microsoft Excel sheet (data dictionary available in Supplementary material—Supplementary Table S2). For each year and partner, 26 variables were collected when available for each isolate, including: country, year, identifier (ID) of the isolate, of the animal and the herd (for livestock species), animal host, bacterial species, specimen (i.e., collection site), antibiotic agent tested for, AST technique (i.e., microdilution, disk diffusion, ETEST^®^) and standards used (i.e., CLSI, EUCAST, AFNOR/CA-SFM Vet), AST quantitative result (minimum inhibitory concentration (MIC) or inhibition zone diameter), as well as production of AmpC- or extended-spectrum beta-lactamase – ESBL, virulence profile (for swine *Escherichia coli*), and presence of *mecA* or *mecC* gene (for *Staphylococcus* spp.). For disk diffusion data, disk concentrations used for AST were also collected. Data were pseudo-anonymized so that no link could be made to the animal or owner of origin.

### Data cleaning

First steps of data processing included corrections of misnamed columns or incorrect data formats (e.g., antibiotics in columns instead of rows). Taking into consideration the study objectives, six variables were deemed essential: year, isolate ID, animal host, bacterial species, antibiotic agent, and MIC/disk diameter value. Entries for which those variables were incorrect, ambiguous, or missing (i.e., thesaurus not respected) were removed from the dataset (Figure 1). Other variables were either considered as optional or could readily be obtained from the data provider. In addition, entries corresponding to combinations of animal species/bacterial species/antibiotic agents outside of the EARS-Vet scope were discarded from the dataset.

Semantic errors in the dataset were also looked for. First, all true duplicated entries (i.e., exact same rows) were deleted. Then we checked that (i) each isolate ID corresponded to a unique animal species, bacterial species, country and year, and (ii) each antibiotic agent tested on a given isolate had a unique MIC/disk diameter value. When one or two of these properties were violated, the data were either corrected or excluded from the dataset. For each partner, feedback was provided in the form of an individual report on data quality, to facilitate data verification and improve future data extractions.

### Data analysis

For each combination of bacterial species–antibiotic, distribution of MIC and disk diameter values were graphically displayed and visually checked for concordance with existing EUCAST epidemiological cut-off values (ECOFFs) or tentative ECOFFs (TECOFFs), when available by May 2022. Of note, several partners tested only for a narrow range of antibiotic concentrations and for a proportion of the isolates provided semi-quantitative MIC data (e.g., “<=2,” “>16”), instead of an exact MIC; these data were arranged by ascending order and visualized on the graph to better capture the overall MIC distribution.

To evaluate proportions of resistance across the years, MIC/disk diameter values were used to categorize isolates as wild-type (WT) or non-wild-type (non-WT) in reference to an antibiotic, using either (T)ECOFFs from EUCAST (for MIC data) (EUCAST, 2022) or epidemiological cut-off values from the CA-SFM Vet (for French inhibition zone diameter data) (Société Française de Microbiologie, 2021), when available.

Combinations of bacterial species–antibiotic agents with no available (T)ECOFF or CA-SFM Vet cut-off were not categorized as WT/non-WT, and were excluded from the analyses. Similarly, MIC data for which the tested concentration ranges were insufficient to allocate WT vs. non-WT categories based on EUCAST (T)ECOFFs were excluded from the analyses. Spanish CLSI disk diffusion data were also excluded from the analyses, since CLSI currently has no epidemiological cut-off values publicly available.

To cope with the fact that partners were testing for different antibiotic agents within certain antibiotic classes, and similarly to what is done by EARS-Net, AMR trends analyses were performed at the level of either antibiotic agent or antibiotic categories, the latter grouping together antibiotic agents with similar resistance mechanisms. Hence, an isolate was considered as resistant to an antibiotic category in case it was resistant to a least one antibiotic agent of the category. For information, a detailed list of antibiotic categories used for each combination of animal species - bacterial species is available in Mader et al. (2022a). In accordance with Arieti et al. (2020), trends were displayed for selected combinations of bacterial species - antibiotic categories with at least 30 isolates per year, animal species and partner (Arieti et al., 2020). Due to small data volumes and to the lack of data on origin of specimens, trends were analyzed including all specimen types together, with the exception of bovine *E. coli* data, where sufficient information was available to split between milk versus other types of specimens. All analyses were performed using R statistical software version 3.6.1 (R Core Team, 2019).

## Results

### Description of available data

From 1,904,658 entries (i.e., combinations of isolate ID - antibiotic agent tested for) initially collected, a total of 1,302,389 entries (68.4%) were retained in further analyses, representing 140,110 isolates and 317 combinations of animal species–bacterial species–antibiotic. Main reasons for exclusion were animal species–bacterial species–antibiotic agent combinations out of the EARS-Vet scope as defined previously (Mader et al., 2022a) or non-respect of thesaurus (Figure 1).

The number of isolates for which data were provided per partner was highly variable, with RESAPATH and SVA being the largest contributors and providing 77.1 and 11.8% of all available isolates, respectively (Table 2). Bacterial isolates originated from cattle (34.2%), dogs (25.7%), chickens (16.2%), swine (13.1%), cats (6.5%) and turkeys (4.3%).

**Table 2:** Number of isolates included per partner and animal category, over 2016–2020.

Country	Partner or program	Number of isolates	Cat	Cattle	Chicken	Dog	Swine	Turkey
Denmark	UCPH	2,303	212	0	0	2,091	0	0
Denmark	Laboratory for Pig Diseases	3,998	0	142	0	0	3,856	0
Germany	LABOKLIN	152	42	0	0	110	0	0
Germany	GE*RM*-Vet	3,272	7	1,597	0	496	1,172	0
Finland	FINRES-Vet	2,340	0	1,464	228	0	584	64
France	RESAPATH	107,965	6,254	43,109	21,542	20,624	10,513	5,923
Italy	AniCura	170	30	0	0	140	0	0
Norway	NORM-VET	559	13	4	175	304	22	41
Spain	SEVAE	1,781	0	3	0	0	1,778	0
Sweden	SVA	16,542	2,407	1,226	447	12,103	353	6
Switzerland	ZOBA	1,028	80	409	292	190	57	0
Total		140,110	9,045	47,954	22,684	36,058	18,335	6,034

Most isolates were *E. coli* (66.1% of all collected isolates) followed by *Staphylococcus pseudintermedius* (14.0%) (Table 3). *E. coli* was the only bacterial species for which data were provided by all countries, and collected for all animal categories. Detailed distributions of isolates per bacterial species and partner are provided in Supplementary Table S3. The amount of data was approximately equally distributed across the 5-year targeted period, with each year including from 18.5 to 21.1% of the data.

**Table 3:** Number of isolates included per bacterial species over 2016–2020.

Bacterial species	Number of isolates	%
*Escherichia coli*	92,671	66.1
*Staphylococcus pseudintermedius*	19,604	14.0
*Streptococcus uberis*	6,435	4.6
*Staphylococcus aureus*	6,269	4.4
*Pasteurella multocida*	5,178	3.7
*Streptococcus suis*	3,005	2.1
*Actinobacillus pleuropneumoniae*	2,228	1.6
*Mannheimia haemolytica*	2,205	1.6
*Streptococcus dysgalactiae*	1,207	0.9
*Klebsiella pneumoniae*	798	0.6
*Staphylococcus hyicus*	510	0.4
Total	140,110	100

Virulence data were provided by four partners (1,850 isolates characterized in total), ESBL/AmpC phenotype confirmation by four partners (140 isolates), and *mecA*/*mecC* presence confirmation by three partners (151 isolates) (Supplementary Table S4).

### Categorization of isolates

Out of the 148 bacterial species–antibiotic agent combinations retained in this study, 81 (54.7%) had a EUCAST (T)ECOFF available for MIC data interpretation. For six of them, the antibiotic concentration ranges used by some laboratories were insufficient to apply the EUCAST (T)ECOFF, leading to discard more than 30% of the collected data for these six combinations; in this case, the data from the partner were removed entirely for this particular bacterial species–antibiotic agent combination. For French disk diffusion data, 146 (98.6%) combinations had a CA-SFM Vet epidemiological cut-off value available (i.e., all except *Streptococcus dysgalactiae*–ceftiofur and *Streptococcus uberis*–ceftiofur).

### AMR trends analysis

**Figure 2:**
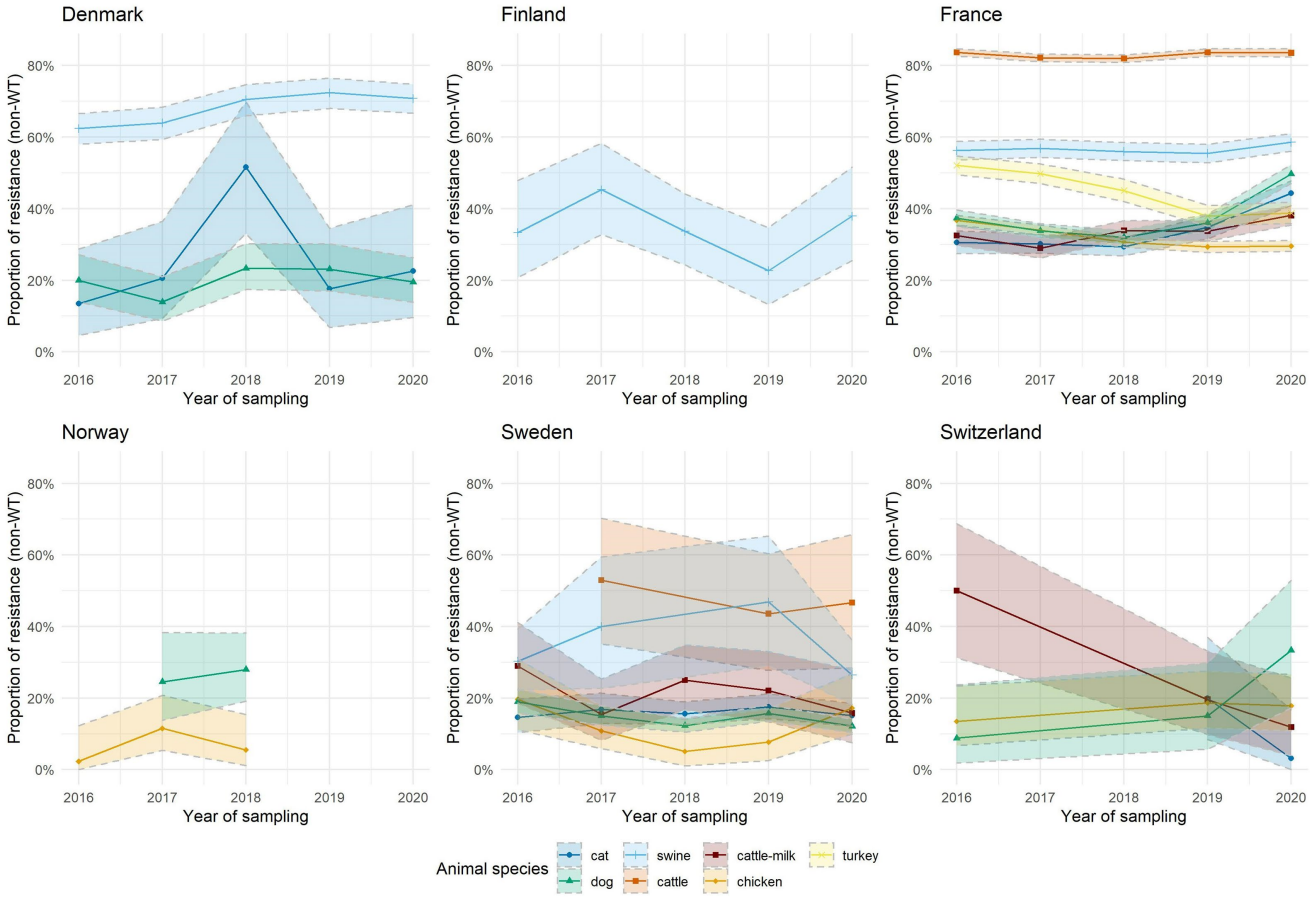
Trends of *E. coli* resistance (non-wild-type) to aminopenicillins over 2016–2020. Only countries and animal species with sufficient data (at least 30 isolates per animal species and per year) are displayed here. Colored areas around the curves represent 95% confidence intervals.

**Figure 3:**
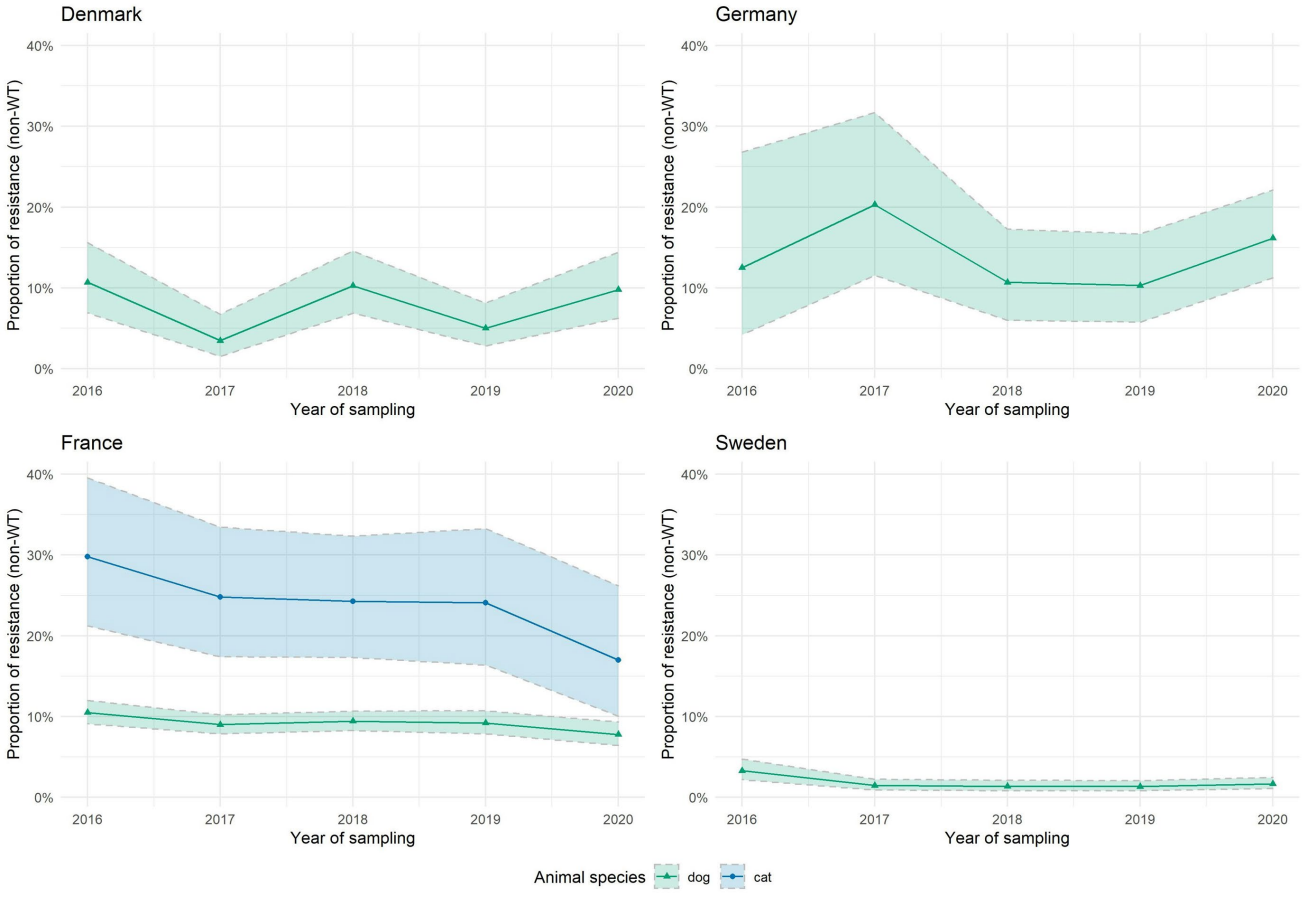
Trends of *S. pseudintermedius* resistance (non-wild-type) to fluoroquinolones over 2016–2020. Only countries and animal species with sufficient data (at least 30 isolates per animal species and per year) are displayed here. Colored areas around the curves represent 95% confidence intervals.

Figures 2, 3 depict AMR trends analyses over 2016–2020 for two selected combinations of bacterial species–antibiotic categories, namely *E. coli* resistant to aminopenicillins and *S. pseudintermedius* resistant to fluoroquinolones. These were selected to illustrate EARS-Vet results for a diversity of bacteria (Gram-positive and Gram-negative), animal species (livestock and companion animals), critically and non-critically important antibiotic classes, and countries. AMR trends for the 51 other combinations of bacterial species–antibiotic categories of interest with sufficient data and available interpretation criteria are provided in supplementary material (Supplementary Figures S1–S51). Figure 2 shows wide variability in the proportion of *E. coli* resistant to aminopenicillins, both within and among countries, although with relatively large confidence intervals due to limited number of isolates in certain countries or animal species. Proportions of resistance generally appeared higher in cattle (approximately 80% in France and 50% in Sweden) and swine (approximately 60% in Denmark and France, 40% in Finland and Sweden) compared to dogs (approximately 40% in France, 30% in Norway and 20% in Denmark, Sweden and Switzerland) and cats (approximately 20 to 50% in Denmark, 30% in France, 20% in Sweden and 5 to 20% in Switzerland). Similarly, the proportion of fluoroquinolone-resistant *S. pseudintermedius* from dogs was variable between countries, with Sweden having proportions below 5%, Denmark and France between 5 and 10%, and Germany between 10 and 20% (Figure 3). Only France provided data for *S. pseudintermedius* in cats, showing higher resistance proportions compared to isolates from dogs but with a decreasing trend.

## Discussion

This study is one of the first attempts to jointly analyze AMR data originating from animal clinical isolates at a European level. It demonstrates that several EARS-Vet partners are already able to nationally centralize large amounts of data, although data volumes were highly variable among partners (with France and Sweden providing most of the data), and from one animal/bacterial species to another. This not only depended on the amount of data owned by the partners, but also on their ability to easily curate and extract them, since the lack of efficient data management tools was reported as a major weakness across existing systems (Mader et al., 2022b). Except for two partners (AniCura and SEVAE), all partners provided data with national geographic coverage. However, considering that all systems relied on passive data collection, often with limited number of isolates, the majority of partners reported their representativeness of the general animal population to be low or unknown (Mader et al., 2022b). Furthermore, some partners were referral clinics or university hospitals, where a high fraction of animals had likely received one or more antibiotic courses prior to sampling for AST, hence leading to potential over-estimation of resistance proportions. Information on prior antimicrobial treatment was not obtained as part of this pilot study, since it is rarely available from routine diagnostic laboratory data. Similar to the EARS-Net methodology (European Centre for Disease Prevention and Control and World Health Organization, 2022), EARS-Vet could consider developing qualitative indicators of coverage and representativeness, e.g., to estimate the proportion of animal populations covered, or the representativeness of included isolates. The likelihood of performing AST when facing a bacterial infection in animals may also vary between countries, animal species, or type of infections (Bourély et al., 2018), and could be captured in a similar way, paralleling the blood-culture rate used in EARS-Net.

Data availability and quality varied among expected variables. While isolate IDs were systematically provided, animal ID and herd ID were poorly informed, since these kinds of data are not commonly recorded by diagnostic laboratories. Although some animals and herds may have been sampled multiple times over the time period of interest, hence introducing some bias in the analyses, it likely represented a negligible proportion of the collected data. Production type was hardly ever available, and dairy *vs* beef should be considered to be replaced by calf *vs* adult cattle, since these tend to have distinct bacterial infections. In addition, dairy *vs* beef production are not always easy to distinguish, since calves born in dairy herds are commonly transferred and later reared for beef production. Similarly, specimen data were difficult to retrieve (except for milk in cattle), and some partners had difficulties to allocate recorded specimen into the EARS-Vet predefined list. Alternatively, EARS-Vet could consider working with a list of predefined infection types (e.g., respiratory, digestive, urinary, mastitis), to which data could be allocated more easily, hence facilitating data interpretation for the next data collection rounds. While almost the entire scope initially envisaged for EARS-Vet (Mader et al., 2022a) was covered by the data collected, some bacteria–antibiotic combinations were poorly covered; for example, resistance data to carbapenems, which are of primary interest for public health, were hardly ever available, since these antibiotics are not authorized in veterinary medicine, and consequently not routinely tested by veterinary diagnostic laboratories. Data on virulence and confirmation of phenotypic resistance profiles using molecular techniques were also limited, since they are not necessarily performed by veterinary diagnostic laboratories and rarely included in national monitoring systems. Such data could be left out for the upcoming data collection rounds, and then reintroduced once more laboratories are able to provide these data.

As described by Mader et al. (2022b), laboratory techniques and standards used by EARS-Vet partners were highly diverse, with a mix of microdilution and disk diffusion techniques, as well as EUCAST, CLSI and national guidelines (Mader et al., 2022b). A strength of this study was to have access to raw MIC and inhibition zone diameter data, making it possible to reinterpret the data using common interpretation criteria. Following previous recommendations from the EARS-Vet expert group (Mader et al., 2022b), and to facilitate early detection of emerging acquired resistance, as well as improve comparability with the EFSA monitoring, it was decided to interpret MIC data using the EUCAST (T)ECOFFs where available, assuming EUCAST and CLSI methods for broth microdilution were equivalent. While this assumption is acceptable for non-fastidious organisms (e.g., *E. coli*, *Staphylococcus* spp.), its applicability to fastidious organisms (e.g., streptococci) is somewhat questionable due to differences between CLSI *vs* EUCAST methodology; this can be considered as a minor limitation to this study. Conversely, recognizing that EUCAST and CLSI standards differ for disk diffusion (e.g., different disk concentrations are used), and since CLSI epidemiological cut-off values (ECVs) are not publicly available, disk diffusion data based on CLSI standards provided by the Spanish partner were excluded from trends analysis. The French data were interpreted with national (CA-SFM Vet) epidemiological cut-off values, since CA-SFM Vet disk diffusion standards differ from EUCAST standards.

Still, working with EUCAST (T)ECOFFs was not straightforward because several partners worked with narrow concentration ranges that did not necessarily include the EUCAST (T)ECOFF value. For those working with MICs, providing exact MICs (instead of semi-quantitative ones) should strongly be recommended for future EARS-Vet data collection. Should these not be routinely available, a representative subset of isolates could be re-tested using MIC panels with a wider range of concentrations. Similar to the EFSA monitoring, EARS-Vet could consider obtaining MIC plates customized to the needs of the network. Tentative concentration ranges to be used for broth microdilution in animal pathogens have recently been proposed (Teale and Borriello, 2021) and could be considered for EARS-Vet. However, even if ideal concentration ranges were provided by all laboratories, this would not solve the problem that EUCAST (T)ECOFFs were missing for 45.3% (MIC) and 76.9% (disk diffusion) of bacterial species – antibiotic agent combinations of interest to EARS-Vet. There is an urgent need for EUCAST to fill this gap. While the VetCAST subcommittee and several initiatives [IMPART (Website of the One Health EJP IMPART project, 2023), ENOVAT (Website of the ENOVAT Cost Action, n.d.)] are working toward defining missing (T)ECOFFs for selected animal pathogen-antibiotic agent combinations using EUCAST standards, there is still a long way to go before all combinations of the tentative EARS-Vet scope (Mader et al., 2022a) are covered with EUCAST (T)ECOFFs. EARS-Vet could also play an active role in collecting and centralizing isolates to be used for defining missing ECOFFs based on EUCAST standards (Mader et al., 2021).

In the meantime, alternative strategies are needed. An option that was initially explored as part of this study was to use CLSI breakpoints (CLSI, 2020) to interpret MIC data with no available EUCAST ECOFFs. However, this solution was discarded, since it would imply mixing up clinical breakpoints with epidemiological cut-off values. It also required extrapolating clinical breakpoints across animal and bacterial species, antibiotics, and types of specimens, since CLSI clinical breakpoints are available for only few combinations of interest to EARS-Vet. After extrapolation, CLSI breakpoints only covered an extra 16% of the 148 EARS-Vet combinations not already covered by an EUCAST (T)ECOFF, while 29% of the combinations had no (T)ECOFF or breakpoints available (data not shown). To parallel the EARS-Net approach, another alternative would be to work with EUCAST clinical breakpoints; however, defining these breakpoints for animal pathogens is still work in progress, e.g., via the VetCAST initiative (Toutain et al., 2017).

Despite these limitations, we managed to perform cross-country data analysis for selected bacterium–antibiotic category combinations with sufficient data entries and available interpretation criteria. This work showed substantial variations in reported levels and trends of resistance, both within and between countries, and between animal species (Figures 2, 3; Supplementary Figures S1–S51). These differences should be taken into account when defining national and European strategies against AMR in animals, in particular when defining antimicrobial treatment guidelines, as well as other antimicrobial stewardship activities in veterinary medicine. Drivers behind those differences also deserve further exploration. It includes, among others, linking EARS-Vet data with national and European trends on antimicrobial use in animals, e.g., using data from the European Surveillance of Veterinary Antimicrobial Consumption (ESVAC) project (European Medicines Agency, 2022). Statistical significance of trends or differences between countries were not assessed at this stage because of concerns about comparability of data from different years. Similarly to the EARS-Net approach, this will be introduced at a later stage, with EARS-Vet progressing step by step toward collection of more comparable data.

To conclude, this pilot study provided a proof-of-concept of what EARS-Vet can achieve, and formed a basis to improve future data collection and analysis. The next steps will consist in adjusting data collection tools, addressing key methodological issues (e.g., lack of ECOFFs and clinical breakpoints), improving harmonization and comparability across countries, and more generally strengthening capacities for AMR surveillance in animal pathogens across Europe. In the mid- to long-term, EARS-Vet aims to become a sustainable initiative, with regular release of surveillance reports and the development of an online dashboard for dissemination of the results to a large audience, hence complementing the existing pool of data to support evidence-based management of AMR in animals in Europe.

## Data availability statement

The raw data supporting the conclusions of this article will be made available by the authors, without undue reservation.

## Ethics statement

Ethical review and approval was not required for the animal study because ethical approval was not necessary since data originated from routine diagnostic laboratories activities. Written informed consent for participation was not obtained from the owners because ethical approval was not necessary since data were anonymized and aggregated, preventing any breach in confidentiality.

## Author contributions

LC, GC, and NJ conceived the study and supervised data collection and analysis. JL performed data cleaning and analysis. J-PA, CB, PD, TG, MH, EJ, HK, BK, AL, J-YM, CS, EM, CM, ON, MN, SN, GO, KP, TP, CJ, AU, and EZ provided data from their country or region and significantly contributed to data analysis and interpretation. KK, RS, and CZ assisted with data analysis and interpretation. LC drafted the original manuscript. All authors substantially edited the text and approved the final version of the manuscript.

## Funding

For Sweden, data collection and reporting was in part funded by the SvarmPat program. For Switzerland, additional financial support was received from the Food Safety and Veterinary Office (FSVO) (grant no. 071-40011-65). Other partners participated with their own funding.

## Conflict of interest

BK and EM work for a commercial veterinary laboratory with EARS-Vet activity not influencing the laboratory work.

The remaining authors declare that the research was conducted in the absence of any commercial or financial relationships that could be construed as a potential conflict of interest.

## Publisher’s note

All claims expressed in this article are solely those of the authors and do not necessarily represent those of their affiliated organizations, or those of the publisher, the editors and the reviewers. Any product that may be evaluated in this article, or claim that may be made by its manufacturer, is not guaranteed or endorsed by the publisher.
