# Supplementary material for: Pilot testing the EARS-Vet surveillance network for antibiotic resistance in bacterial pathogens from animals in the EU/EEA
Source: Front Microbiol. 2023 May 22;14:1188423. doi: 10.3389/fmicb.2023.1188423 (PMC10239921; doi:10.3389/fmicb.2023.1188423)
Supplement: Supplementary file 1 [file Data_Sheet_1.pdf]

Supplementary Tables

Table S1. Distribution of laboratory techniques and standards use by each partner or programme

Country	Partner or programme	AST technique	Microdilution technique	AST standard	Bacterial identification technique
**Denmark**	UCPH	microdilution	commercial microtiter plates	CLSI	MALDI-TOF MS
**Denmark**	Laboratory for Pig Diseases	microdilution	commercial microtiter plates	CLSI	Colony morphology, selective agars, agglutinations or MALDI-TOF MS
**Finland**	FINRES-Vet	microdilution	commercial microtiter plates	CLSI	API galleries or MALDI-TOF MS
**France**	RESAPATH	disk diffusion		CA-SFM Vet	API galleries or MALDI-TOF MS
**Germany**	GE*RM*-Vet	microdilution	commercial microtiter plates	CLSI	MALDI-TOF MS
**Germany**	LABOKLIN	microdilution	MERLIN Diagnostika GmbH	CLSI	MALDI-TOF MS
**Italy**	AniCura	microdilution	VITEK 2®	CLSI	MALDI-TOF MS
**Norway**	NORM-VET	microdilution	commercial microtiter plates	ISO 20776-1:2019 (EUCAST)	MALDI-TOF MS
**Spain**	SEVAE	microdilution, disk diffusion	commercial microtiter plates	CLSI	VITEK or MALDI-TOF MS
**Sweden**	SVA	microdilution	commercial microtiter plates	ISO 20776-1:2019 (EUCAST)	MALDI-TOF MS
**Switzerland**	ZOBA	microdilution	commercial microtiter plates	CLSI	MALDI-TOF MS

Table S2. EARS-Vet data dictionary

**Name of the variable**	**Expected values**
**Isolate ID**	Fully-anonymized or pseudo-anonymized ID, for example i_FR1, i_FR2, i_FR3 (for the list of French isolates)
**Animal ID**	Fully- anonymized or pseudo-anonymized ID, for example a_FR1, a_FR2, a_FR3
**Herd ID**	Fully- anonymized or pseudo-anonymized ID, for example h_FR1, h_FR2, h_FR3
**Country**	
**Year of sampling**	2016 to 2020
**Animal host**	cat / cattle / chicken / dog / turkey / swine
**Production type**	broiler / layer / dairy / beef / piglet / fattener / sow / not available
**Specimen**	ear/ faeces / inner organs / joints / lung / milk / skin / urine / not available
**Bacterial species**	*Actinobacillus pleuropneumoniae / Escherichia coli / Klebsiella pneumoniae / Mannheimia haemolytica / Pasteurella multocida / Staphylococcus aureus / Staphylococcus hyicus / Staphylococcus pseudintermedius / Streptococcus dysgalactiae / Streptococcus suis / Streptococcus uberis*
**AST technique**	microdilution / disk diffusion
**AST standard**	CLSI / EUCAST / CA-SFM Vet
**Antibiotic agent**	amoxicillin / amoxicillin + clavulanic acid / ampicillin / cephalexin / cefalotin / cefazolin / cefepime / cefotaxime / cefovecin / cefoxitin / cefpodoxime / cefquinome / ceftiofur / chloramphenicol / ciprofloxacin / colistin / daptomycin / doxycycline / enrofloxacin / ertapenem / erythromycin / florfenicol / flumequine / gentamicin / imipenem / lincomycin / linezolid / marbofloxacin / meropenem / moxifloxacin / nalidixic acid / neomycin / oxacillin / oxytetracycline / penicillin / piperacillin-tazobactam / rifampicin / streptomycin / sulfamethoxazole – trimethoprim / tetracycline / tiamulin / tigecycline / tilmicosin / tulathromycin / vancomycin
**Disk diameter or MIC value**	Value of the inhibition zone diameter (in mm) or the minimum inhibitory concentration (in mg/L or µg/mL)
**Disk concentration**	Concentration of the antibiotic drug in the disc (in µg)
**Virulence testing technique**	PCR / serotyping / haemolysis / haemolysis+PCR / other
**Virulent strain**	yes / no / not available
**ESBL/AmpC confirmation technique**	disk diffusion / EUVSEC2 panel / chromagar / PCR / other
**ESBL/ AmpC profile**	yes / no / not available
**PCR *mecA* or *mecC* positive**	yes / no / not available

Table S3. Number of isolates included per bacterial species and partner or programme

**Bacterial species**	**AniCura**	**FINRES-Vet**	**GE*RM*-Vet**	**LABOKLIN**	**Laboratory for Pig Diseases**	**NORM-VET**	**RESAPATH**	**SEVAE**	**SVA**	**UCPH**	**ZOBA**
Actinobacillus pleuropneumoniae	0	136	136	0	649	21	828	458	0	0	0
Escherichia coli	166	508	0	47	2369	378	78723	743	8091	989	657
Klebsiella pneumoniae	0	0	0	0	0	4	580	0	214	0	0
Mannheimia haemolytica	0	334	373	0	80	0	1393	2	23	0	0
Pasteurella multocida	0	1130	915	0	151	0	2419	219	344	0	0
Staphylococcus aureus	4	118	0	30	0	0	5466	0	465	63	123
Staphylococcus hyicus	0	52	118	0	49	0	237	43	11	0	0
Staphylococcus pseudintermedius	0	0	503	75	0	156	10223	0	7311	1251	85
Streptococcus dysgalactiae	0	0	136	0	0	0	1053	0	18	0	0
Streptococcus suis	0	62	663	0	700	0	1236	316	28	0	0
Streptococcus uberis	0	0	428	0	0	0	5807	0	37	0	163

Table S4. Number of isolates with confirmed virulence, ESBL/AmpC or *mec*A/*mec*C profiles

**Country**	**Partner or programme**	**Virulence confirmation***	**ESBL/AmpC confirmation**	***mecA*/*mecC* confirmation****
Denmark	UCPH	-	-	-
Denmark	Laboratory for Pig Diseases	1,343	-	-
Germany	LABOKLIN	-	47	-
Germany	GE*RM*-Vet	-	-	24
Finland	FINRES-Vet	263	55	7
France	RESAPATH	-	-	-
Italy	AniCura	-	-	-
Norway	NORM-VET	-	3	-
Spain	SEVAE	-	-	-
Sweden	SVA	242	35	120
Switzerland	ZOBA	2	-	-
**Total**		**1,850**	**140**	**151**

* In accordance with the EARS-Vet scope, virulence date were collected only for swine *E. coli*

** *mecA* / *mecC* confirmation only applied to *Staphylococcus* spp.
