# Supplementary material for: Pilot testing the EARS-Vet surveillance network for antibiotic resistance in bacterial pathogens from animals in the EU/EEA
Source: Front Microbiol. 2023 May 22;14:1188423. doi: 10.3389/fmicb.2023.1188423 (PMC10239921; doi:10.3389/fmicb.2023.1188423)
Supplement: Supplementary file 2 [file Data_Sheet_2.pdf]

Supplementary Figures

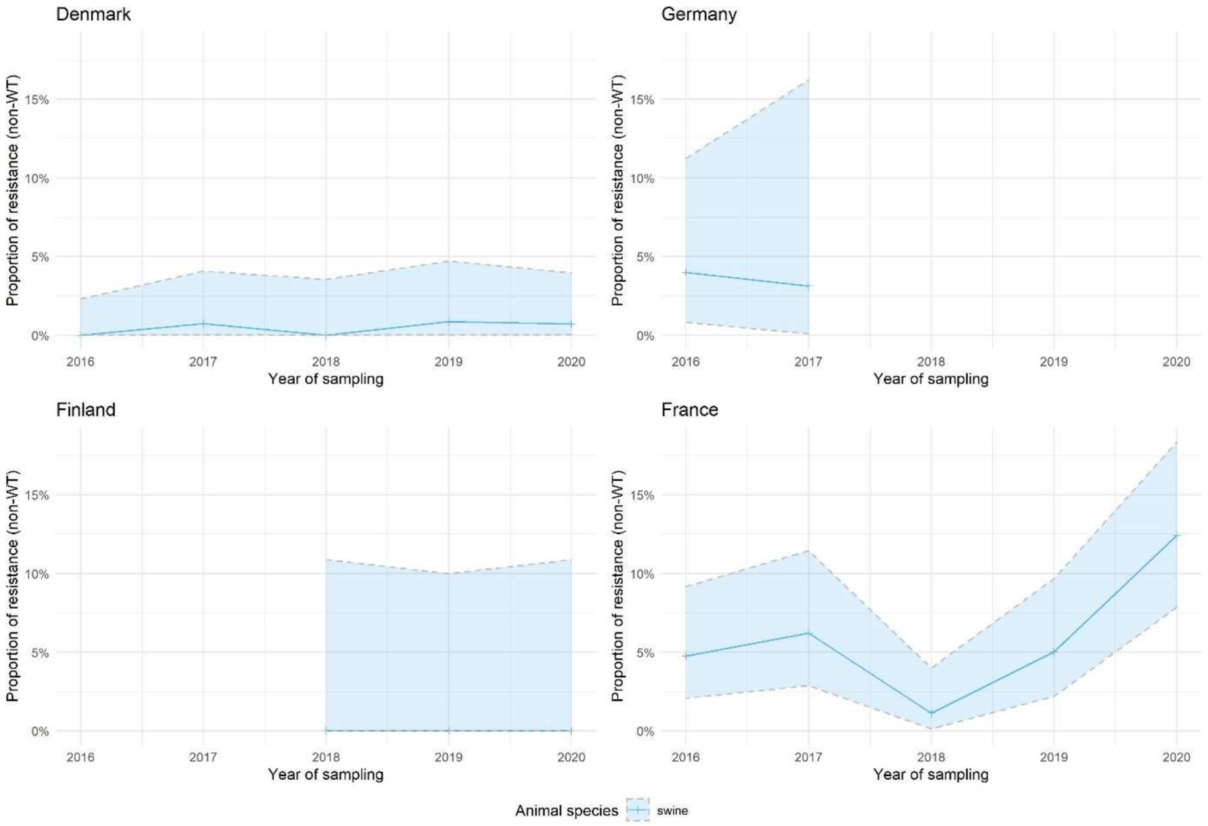

Figure S1. Trends of *Actinobacillus pleuropneumoniae* resistance (non-wild-type) to aminopenicillins over 2016-2020

Only countries and animal species with sufficient data (at least 30 isolates per animal species and per year) are displayed on the figures.

Coloured areas around the curves represent 95% confidence intervals.

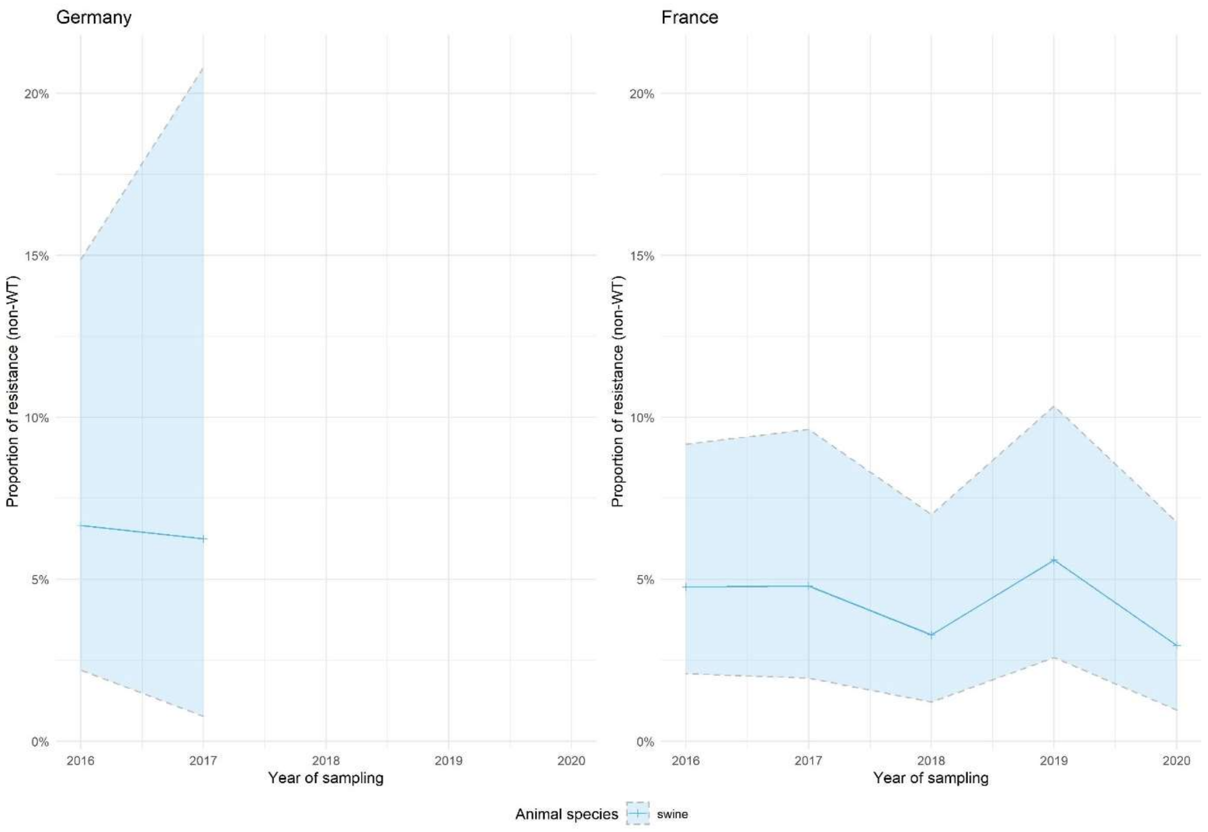

Figure S2. Trends of *Actinobacillus pleuropneumoniae* resistance (non-wild-type) to sulfonamide-trimethoprim over 2016-2020

Only countries and animal species with sufficient data (at least 30 isolates per animal species and per year) are displayed on the figures.

Coloured areas around the curves represent 95% confidence intervals.

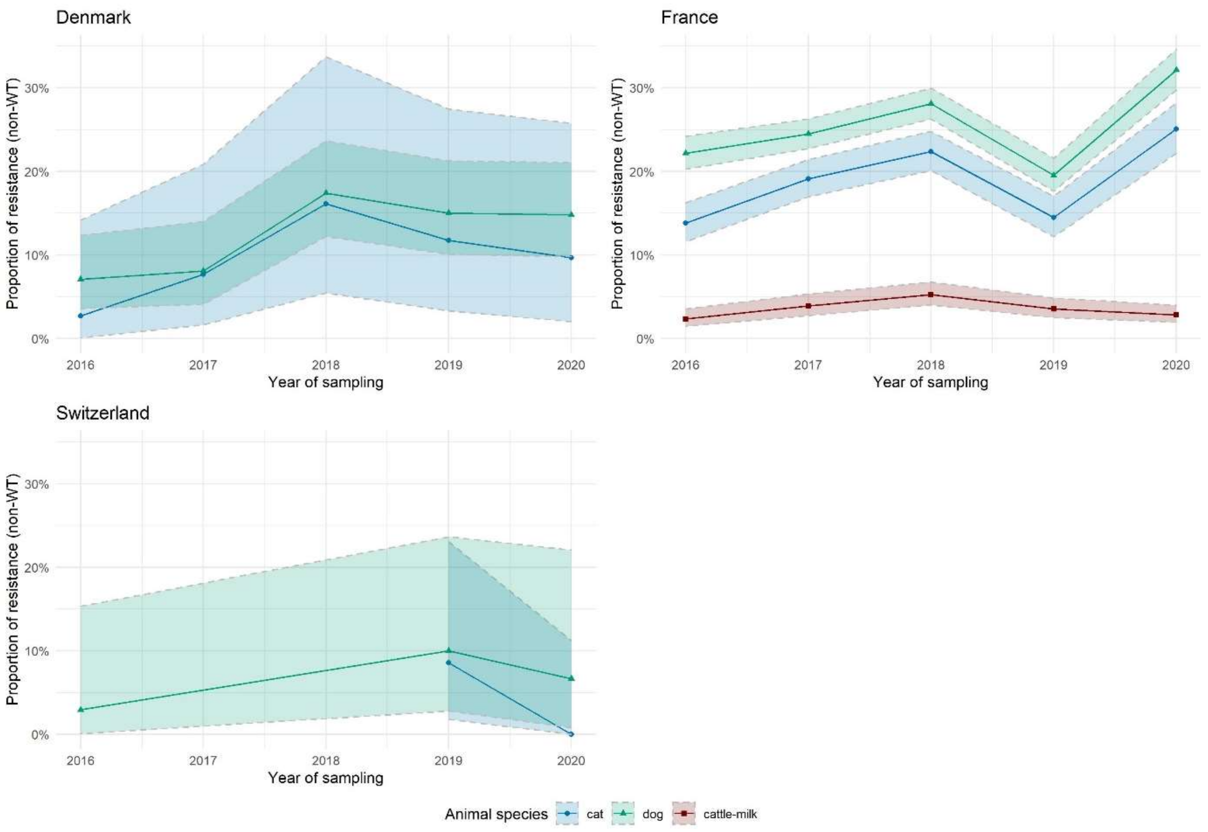

Figure S3. Trends of *Escherichia coli* resistance (non-wild-type) to second-generation cephalosporins over 2016-2020

Only countries and animal species with sufficient data (at least 30 isolates per animal species and per year) are displayed on the figures.

Coloured areas around the curves represent 95% confidence intervals.

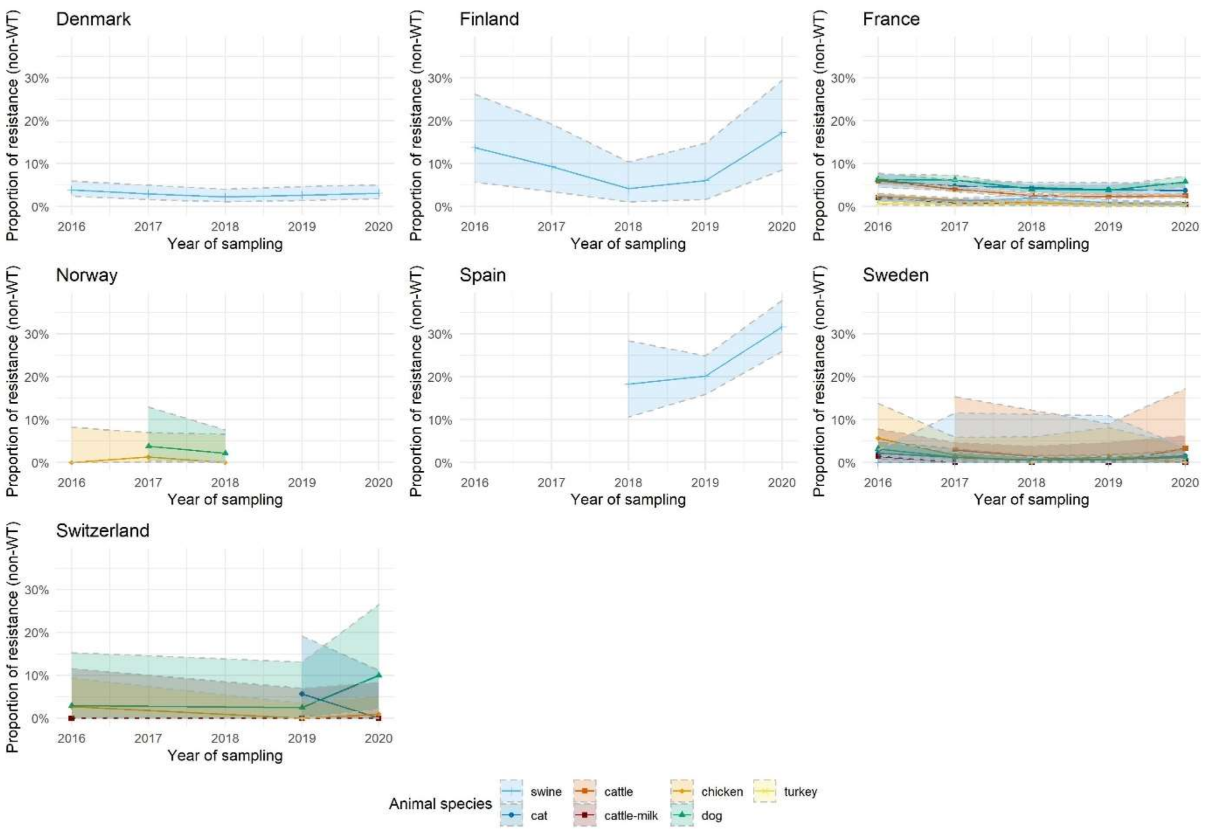

Figure S4. Trends of *Escherichia coli* resistance (non-wild-type) to third-generation cephalosporins over 2016-2020

Only countries and animal species with sufficient data (at least 30 isolates per animal species and per year) are displayed on the figures.

Coloured areas around the curves represent 95% confidence intervals.

Data from Spain (North-eastern Spain) are regional data, hence cannot be considered as representative from the entire country.

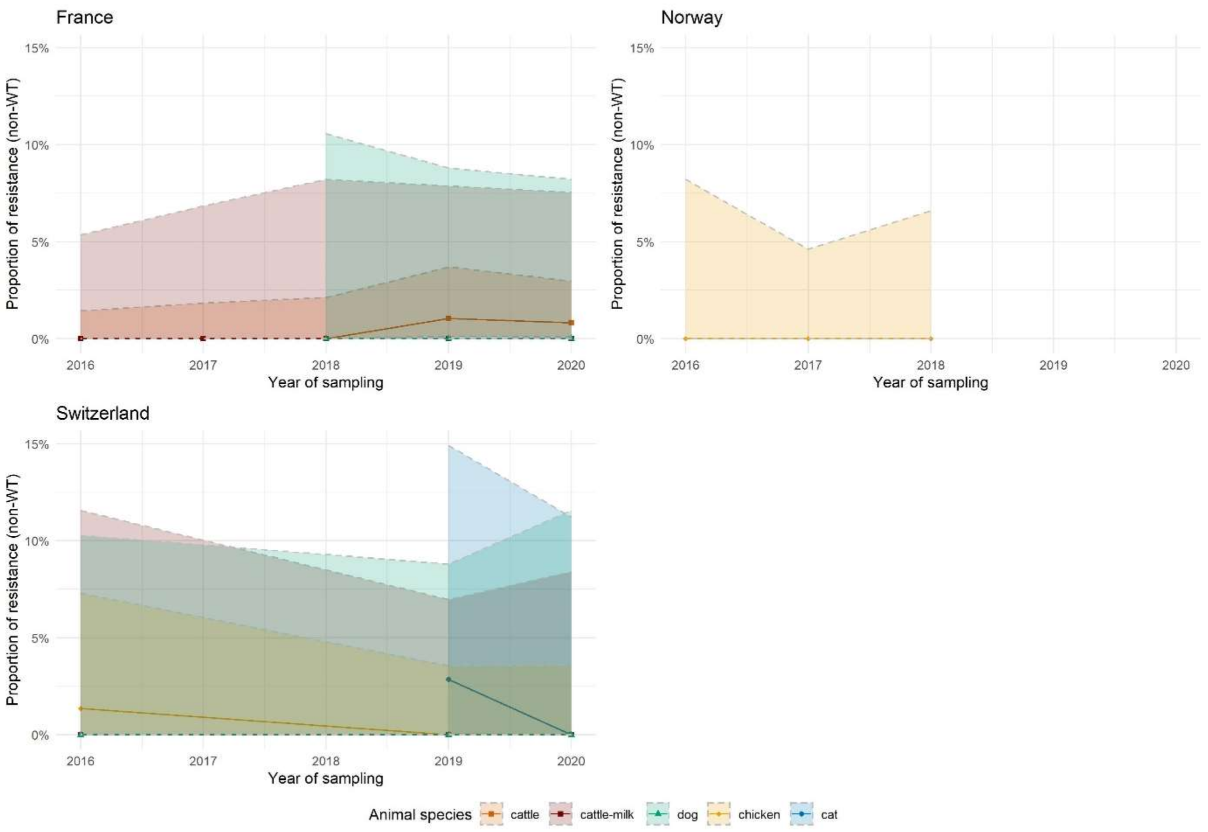

Figure S5. Trends of *Escherichia coli* resistance (non-wild-type) to carbapenems over 2016-2020

Only countries and animal species with sufficient data (at least 30 isolates per animal species and per year) are displayed on the figures.

Coloured areas around the curves represent 95% confidence intervals.

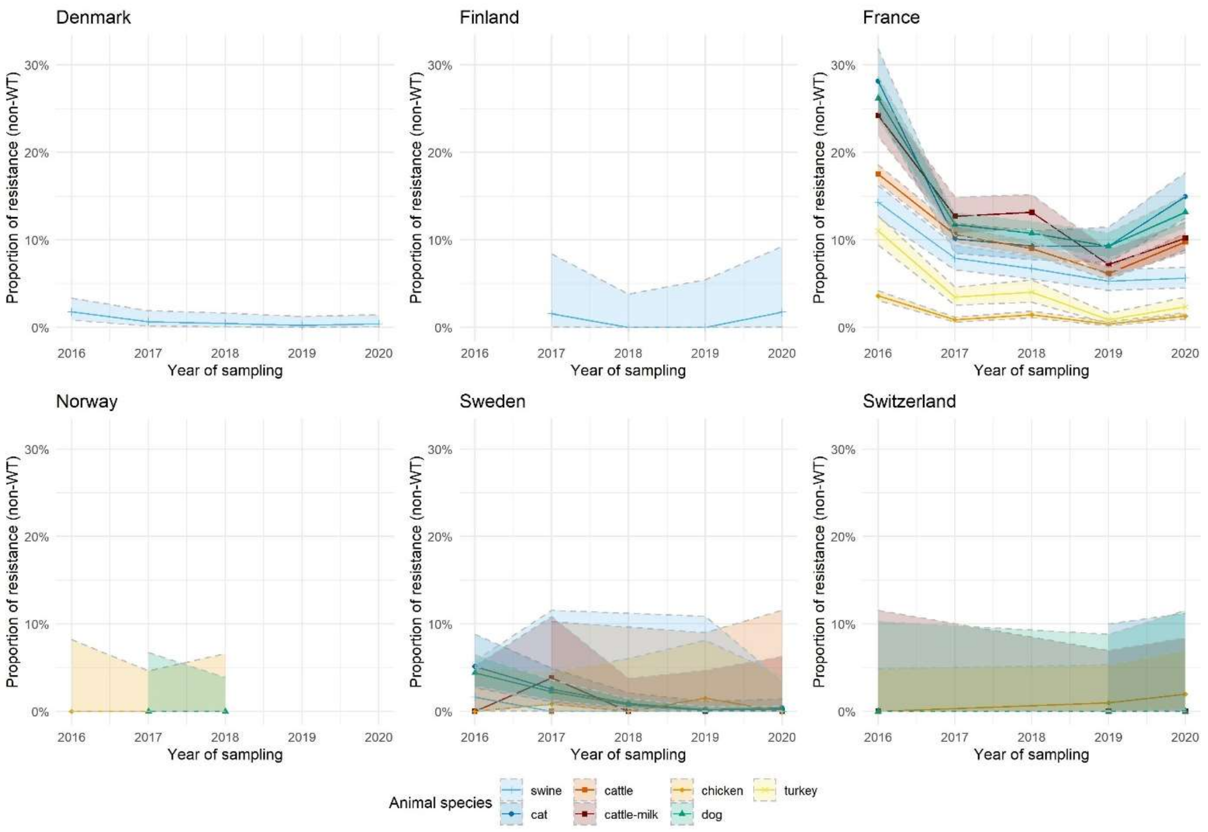

Figure S6. Trends of *Escherichia coli* resistance (non-wild-type) to colistin over 2016-2020

Only countries and animal species with sufficient data (at least 30 isolates per animal species and per year) are displayed on the figures.

Coloured areas around the curves represent 95% confidence intervals.

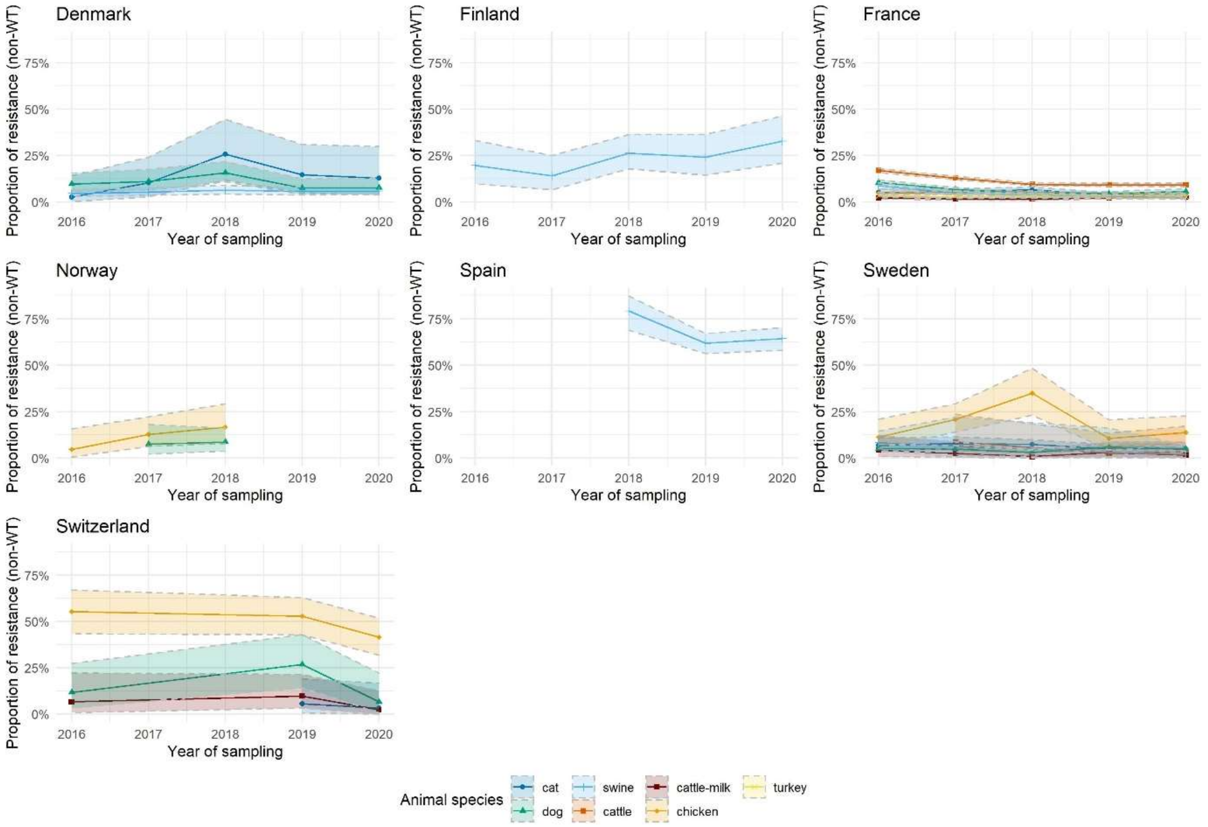

Figure S7. Trends of *Escherichia coli* resistance (non-wild-type) to fluoroquinolones over 2016-2020

Only countries and animal species with sufficient data (at least 30 isolates per animal species and per year) are displayed on the figures.

Coloured areas around the curves represent 95% confidence intervals.

Data from Spain (North-eastern Spain) are regional data, hence cannot be considered as representative from the entire country.

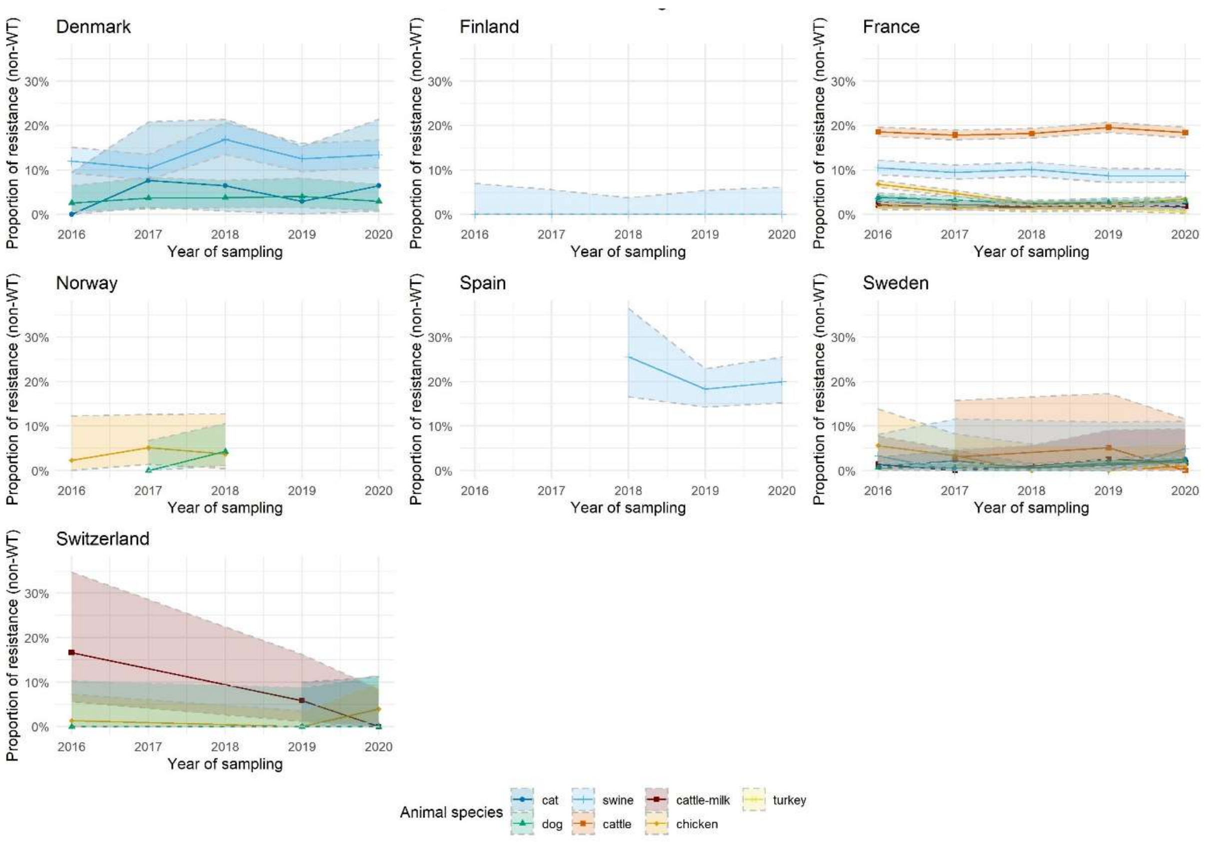

Figure S8. Trends of *Escherichia coli* resistance (non-wild-type) to gentamicin over 2016-2020

Only countries and animal species with sufficient data (at least 30 isolates per animal species and per year) are displayed on the figures.

Coloured areas around the curves represent 95% confidence intervals.

Data from Spain (North-eastern Spain) are regional data, hence cannot be considered as representative from the entire country.

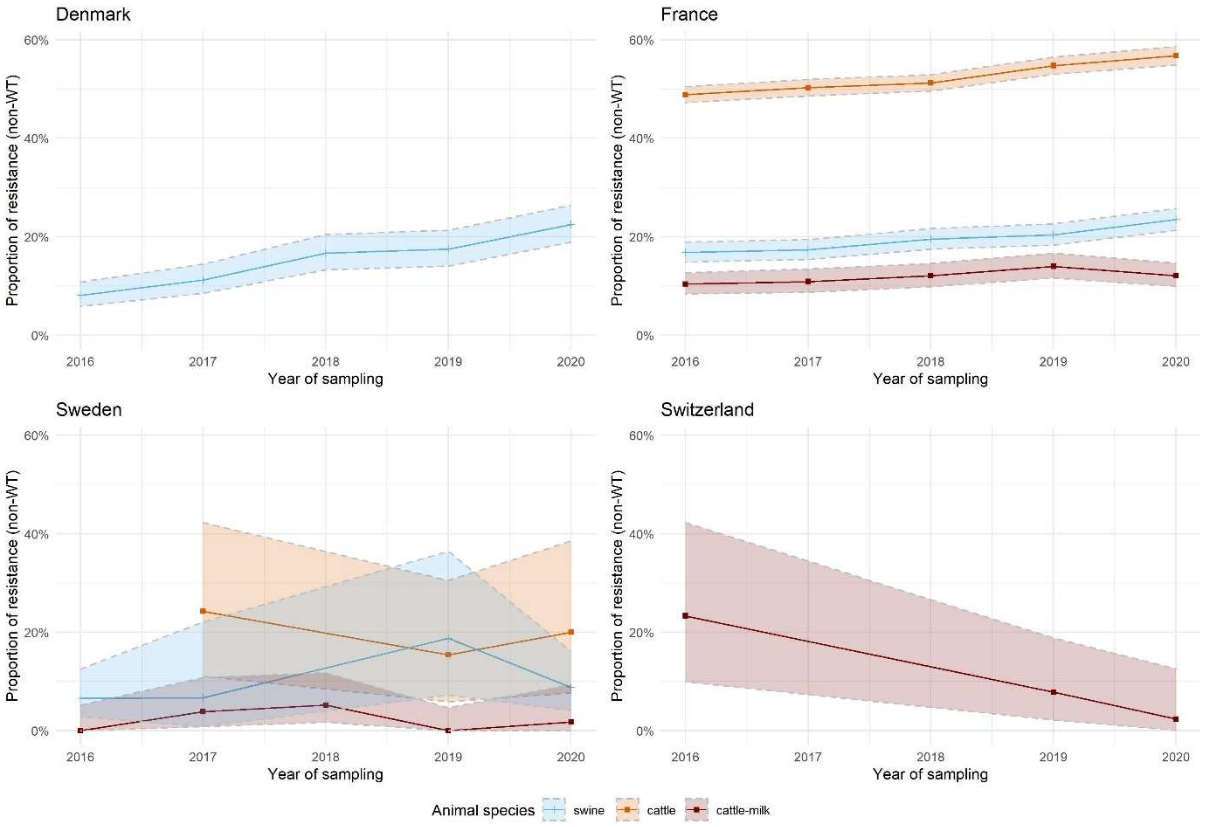

Figure S9. Trends of *Escherichia coli* resistance (non-wild-type) to neomycin over 2016-2020

Only countries and animal species with sufficient data (at least 30 isolates per animal species and per year) are displayed on the figures.

Coloured areas around the curves represent 95% confidence intervals.

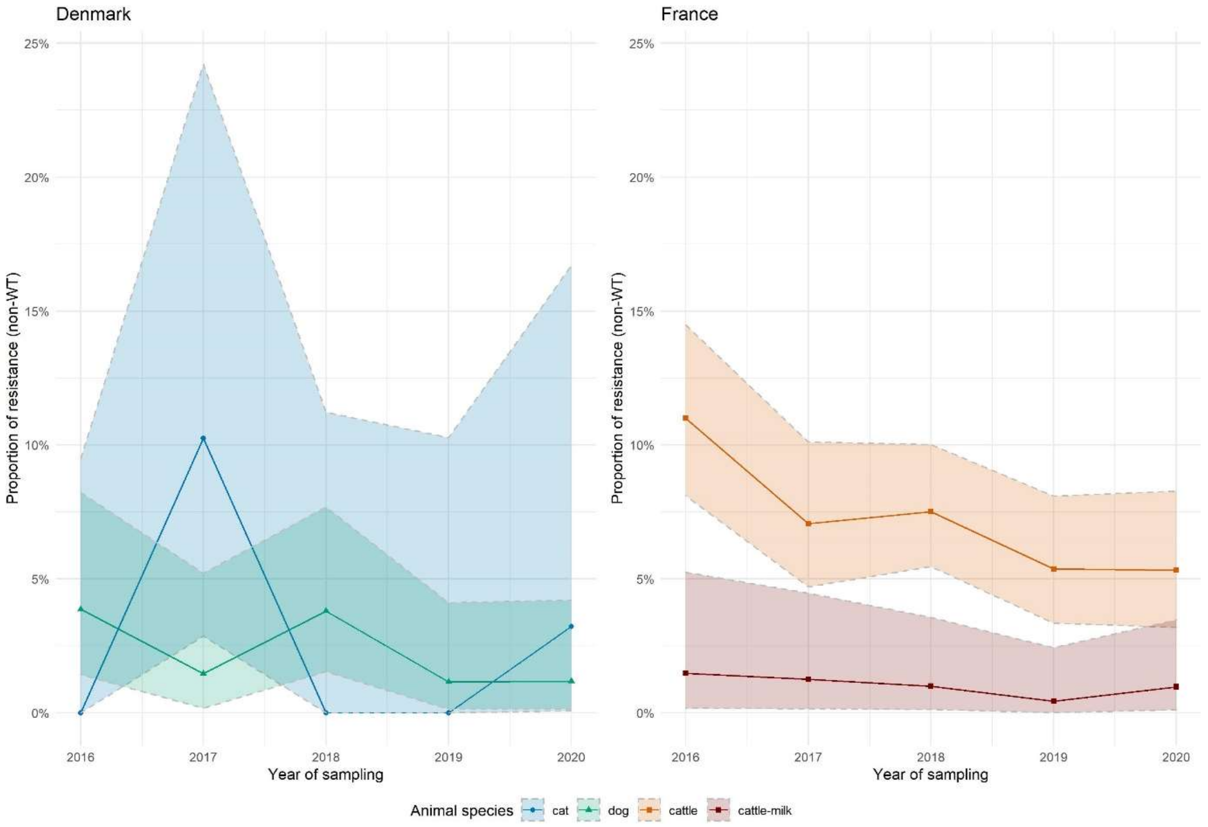

Figure S10. Trends of *Escherichia coli* resistance (non-wild-type) to piperacillin-tazobactam over 2016-2020

Only countries and animal species with sufficient data (at least 30 isolates per animal species and per year) are displayed on the figures.

Coloured areas around the curves represent 95% confidence intervals.

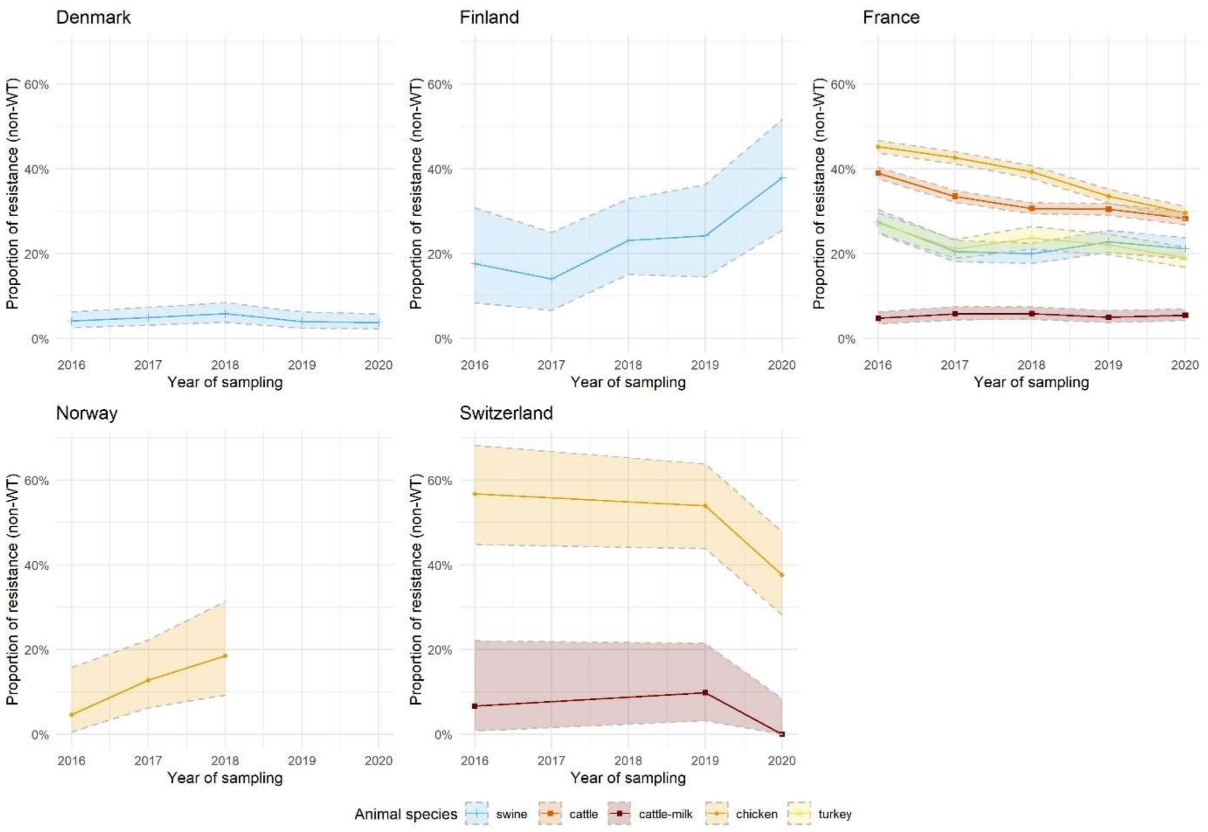

Figure S11. Trends of *Escherichia coli* resistance (non-wild-type) to quinolones over 2016-2020

Only countries and animal species with sufficient data (at least 30 isolates per animal species and per year) are displayed on the figures.

Coloured areas around the curves represent 95% confidence intervals.

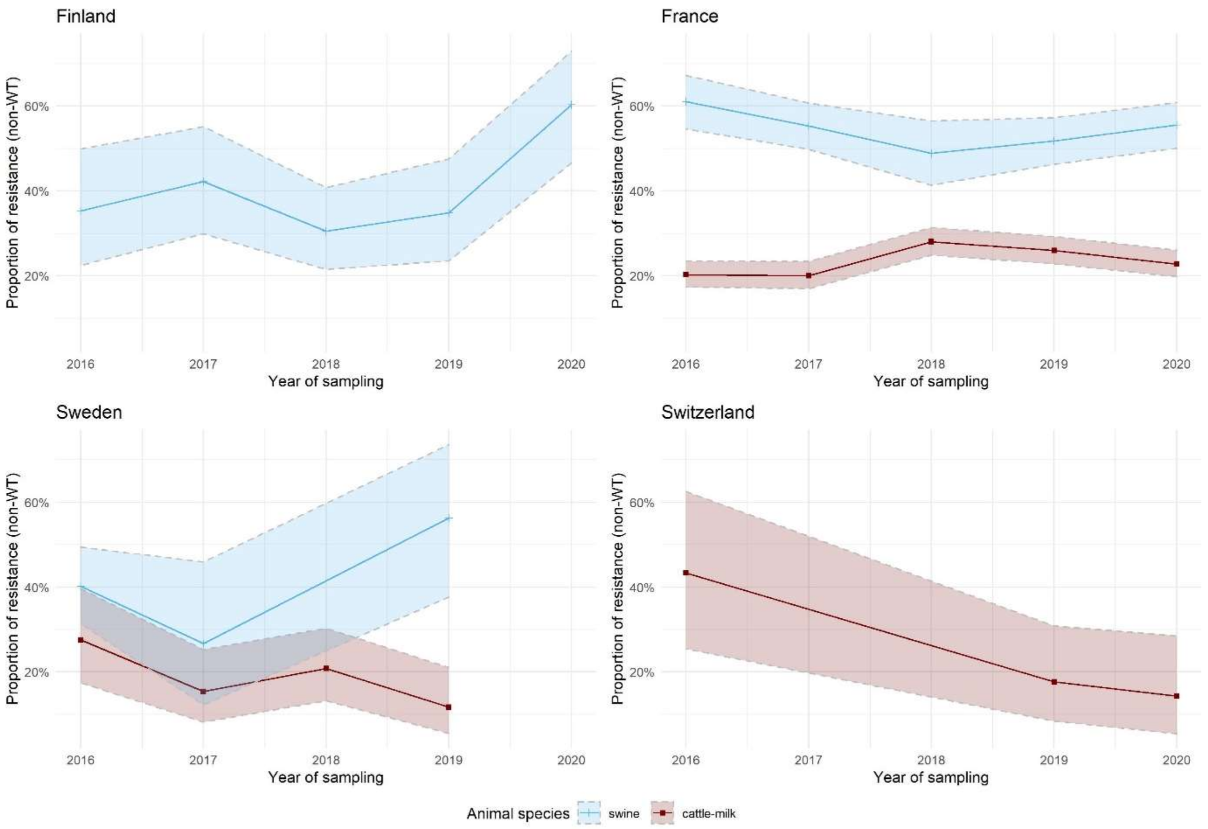

Figure S12. Trends of *Escherichia coli* resistance (non-wild-type) to streptomycin over 2016-2020

Only countries and animal species with sufficient data (at least 30 isolates per animal species and per year) are displayed on the figures.

Coloured areas around the curves represent 95% confidence intervals.

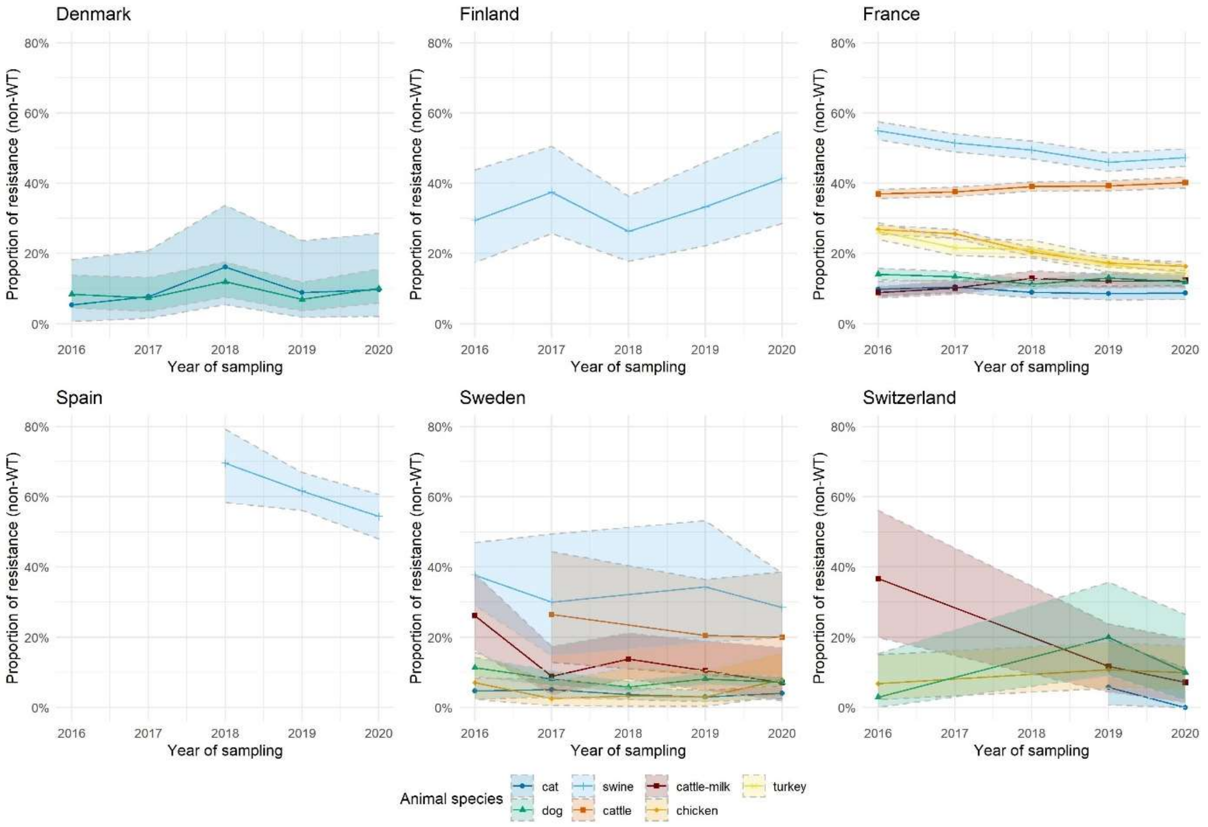

Figure S13. Trends of *Escherichia coli* resistance (non-wild-type) to sulfonamide-trimethoprim over 2016-2020

Only countries and animal species with sufficient data (at least 30 isolates per animal species and per year) are displayed on the figures.

Coloured areas around the curves represent 95% confidence intervals.

Data from Spain (North-eastern Spain) are regional data, hence cannot be considered as representative from the entire country.

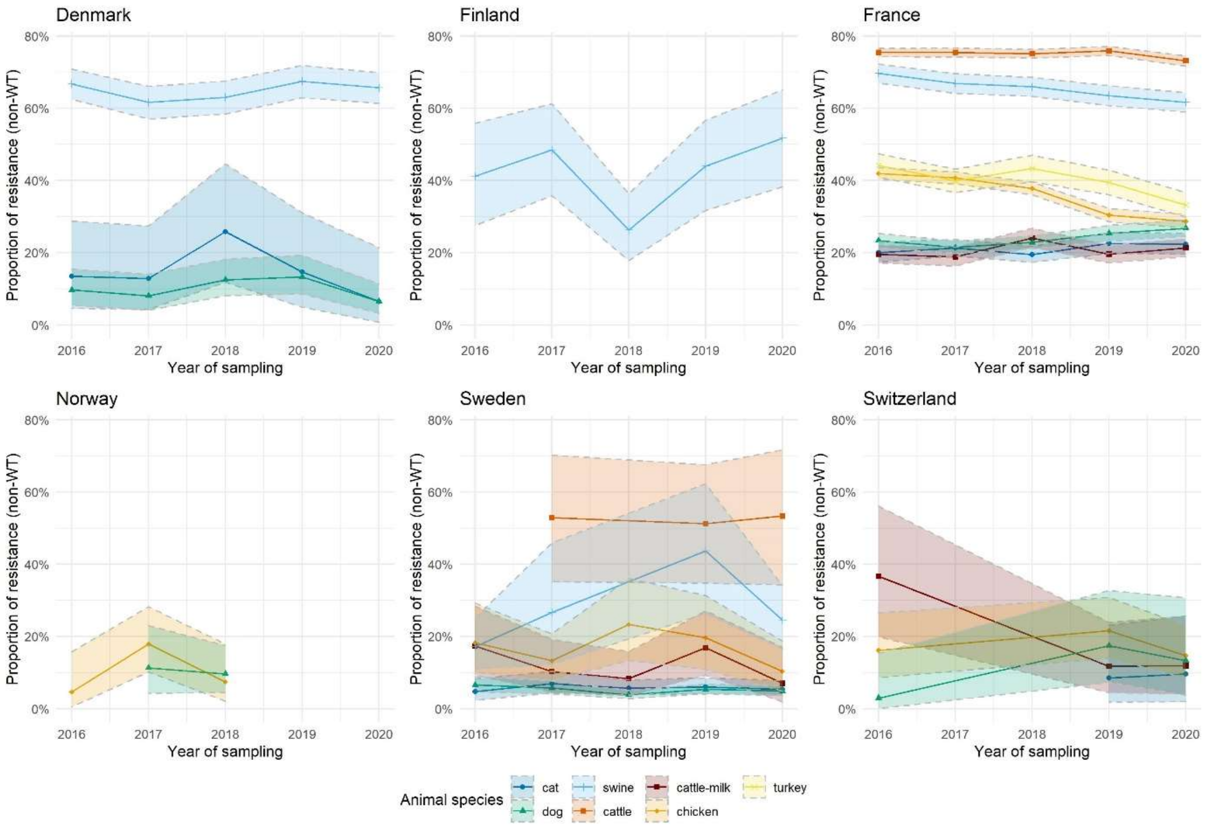

Figure S14. Trends of *Escherichia coli* resistance (non-wild-type) to tetracyclines over 2016-2020

Only countries and animal species with sufficient data (at least 30 isolates per animal species and per year) are displayed on the figures.

Coloured areas around the curves represent 95% confidence intervals.

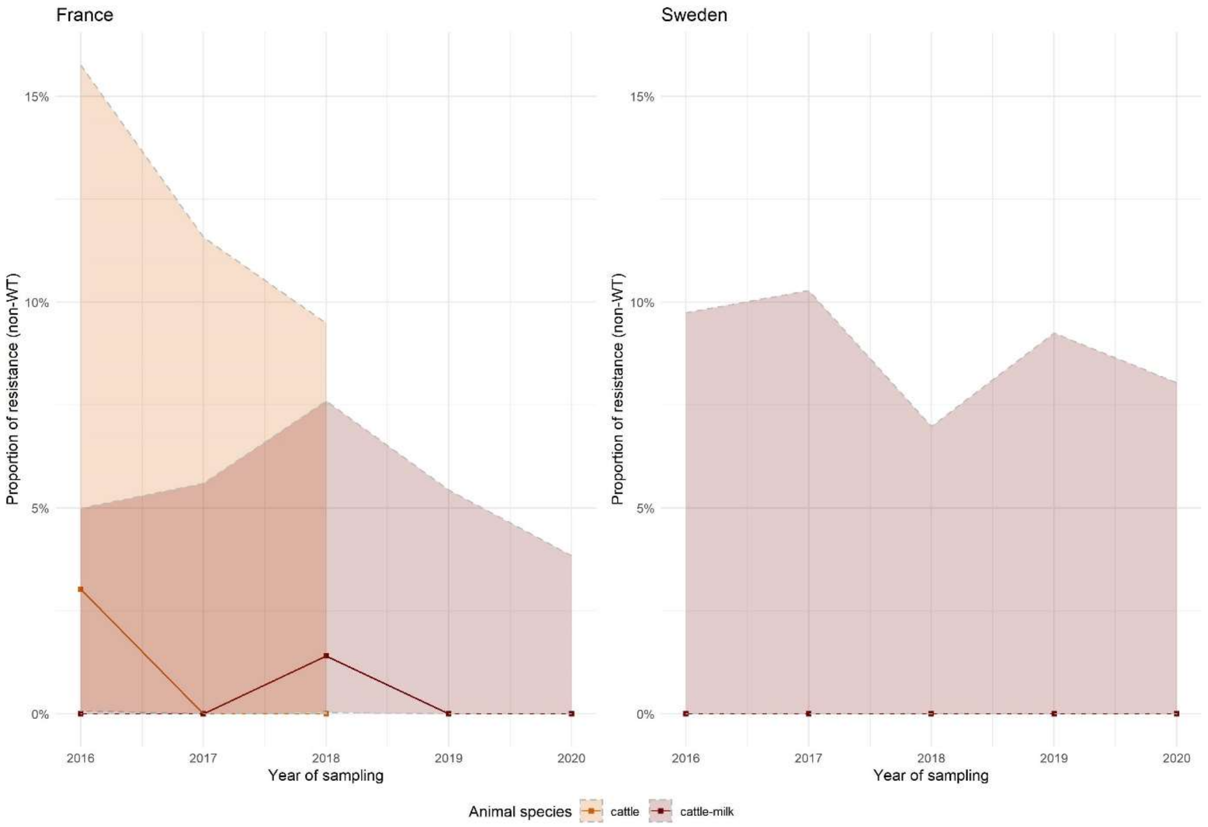

Figure S15. Trends of *Klebsiella pneumoniae* resistance (non-wild-type) to third-generation cephalosporins over 2016-2020

Only countries and animal species with sufficient data (at least 30 isolates per animal species and per year) are displayed on the figures.

Coloured areas around the curves represent 95% confidence intervals.

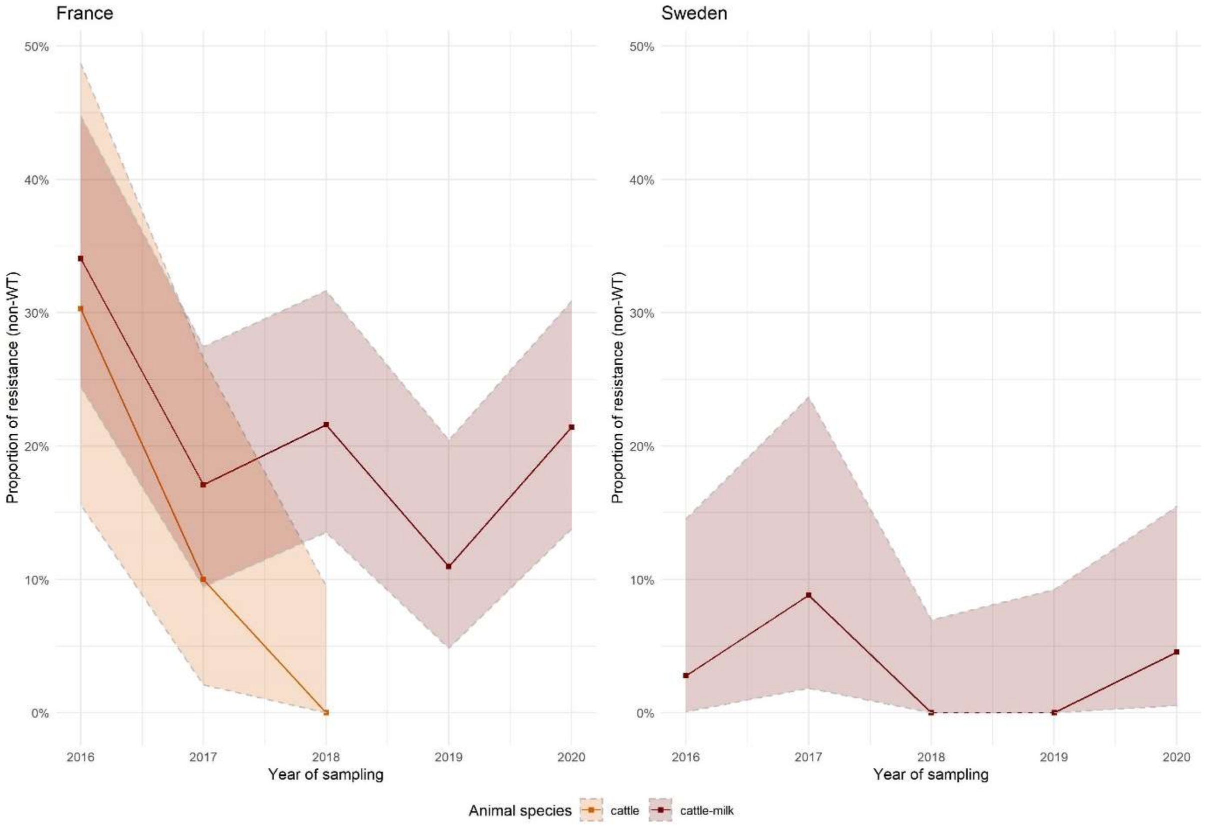

Figure S16. Trends of *Klebsiella pneumoniae* resistance (non-wild-type) to colistin over 2016-2020

Only countries and animal species with sufficient data (at least 30 isolates per animal species and per year) are displayed on the figures.

Coloured areas around the curves represent 95% confidence intervals.

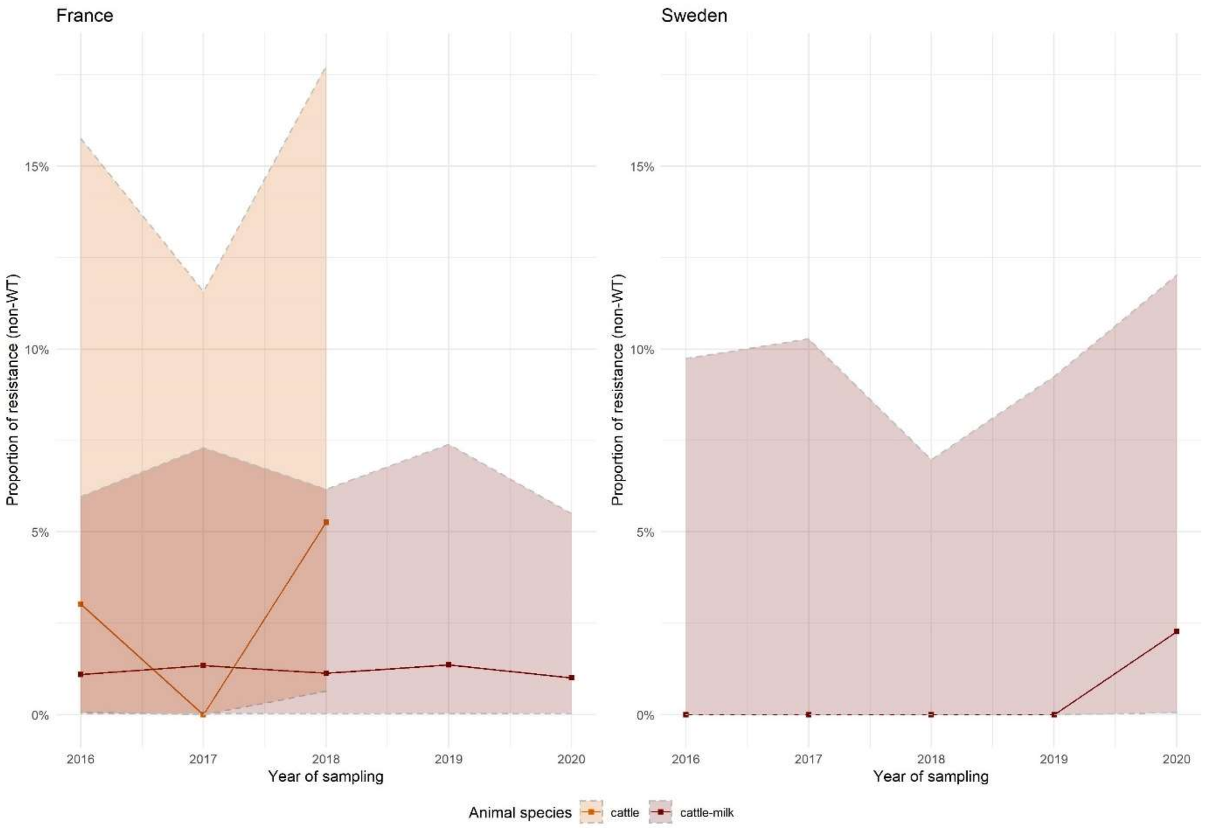

Figure S17. Trends of *Klebsiella pneumoniae* resistance (non-wild-type) to gentamicin over 2016-2020

Only countries and animal species with sufficient data (at least 30 isolates per animal species and per year) are displayed on the figures.

Coloured areas around the curves represent 95% confidence intervals.

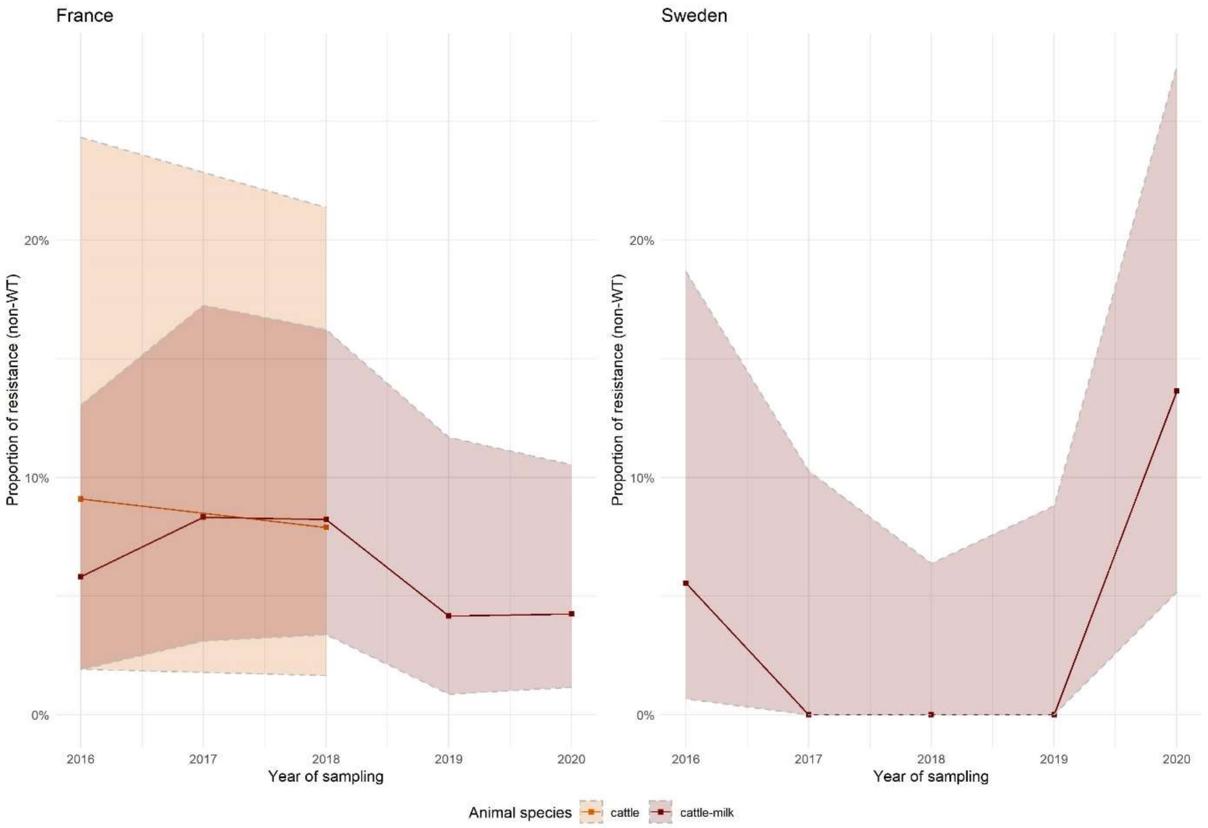

Figure S18. Trends of *Klebsiella pneumoniae* resistance (non-wild-type) to sulfonamide-trimethoprim over 2016-2020

Only countries and animal species with sufficient data (at least 30 isolates per animal species and per year) are displayed on the figures.

Coloured areas around the curves represent 95% confidence intervals.

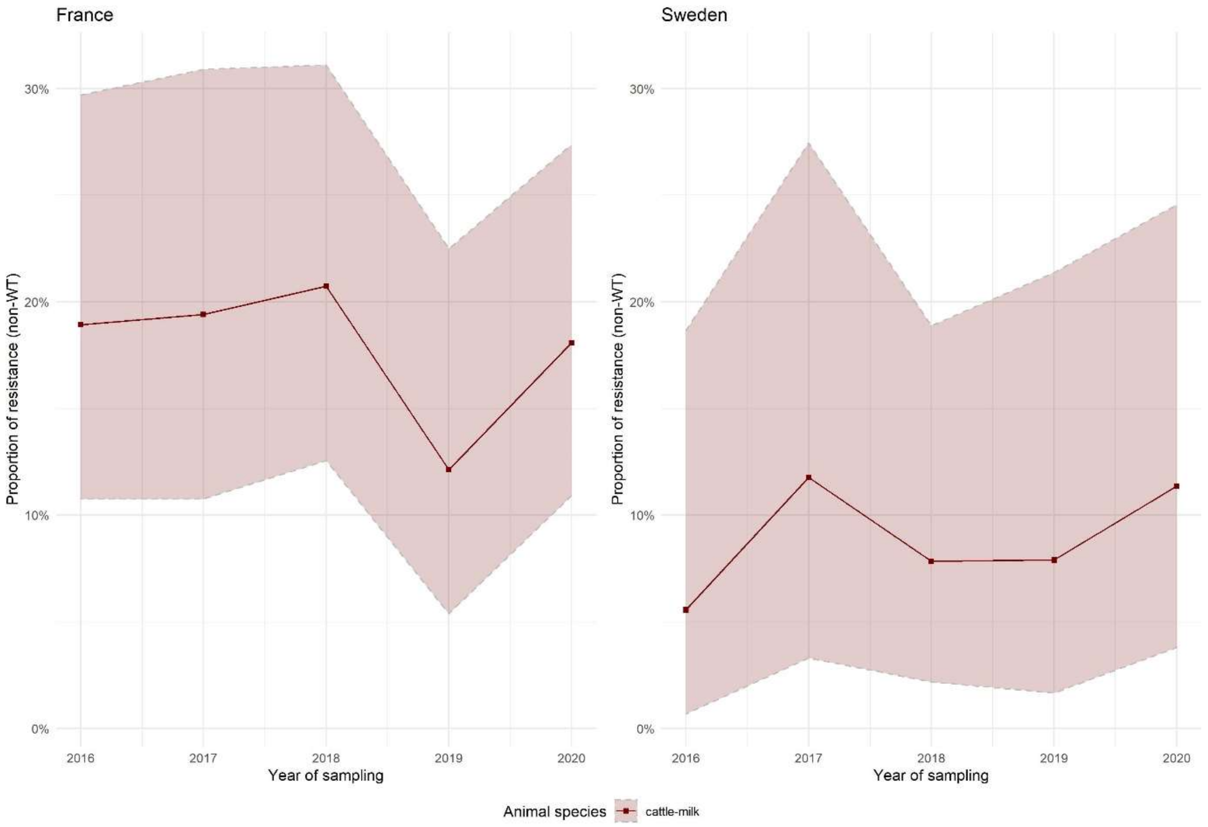

Figure S19. Trends of *Klebsiella pneumoniae* resistance (non-wild-type) to tetracyclines over 2016-2020

Only countries and animal species with sufficient data (at least 30 isolates per animal species and per year) are displayed on the figures.

Coloured areas around the curves represent 95% confidence intervals.

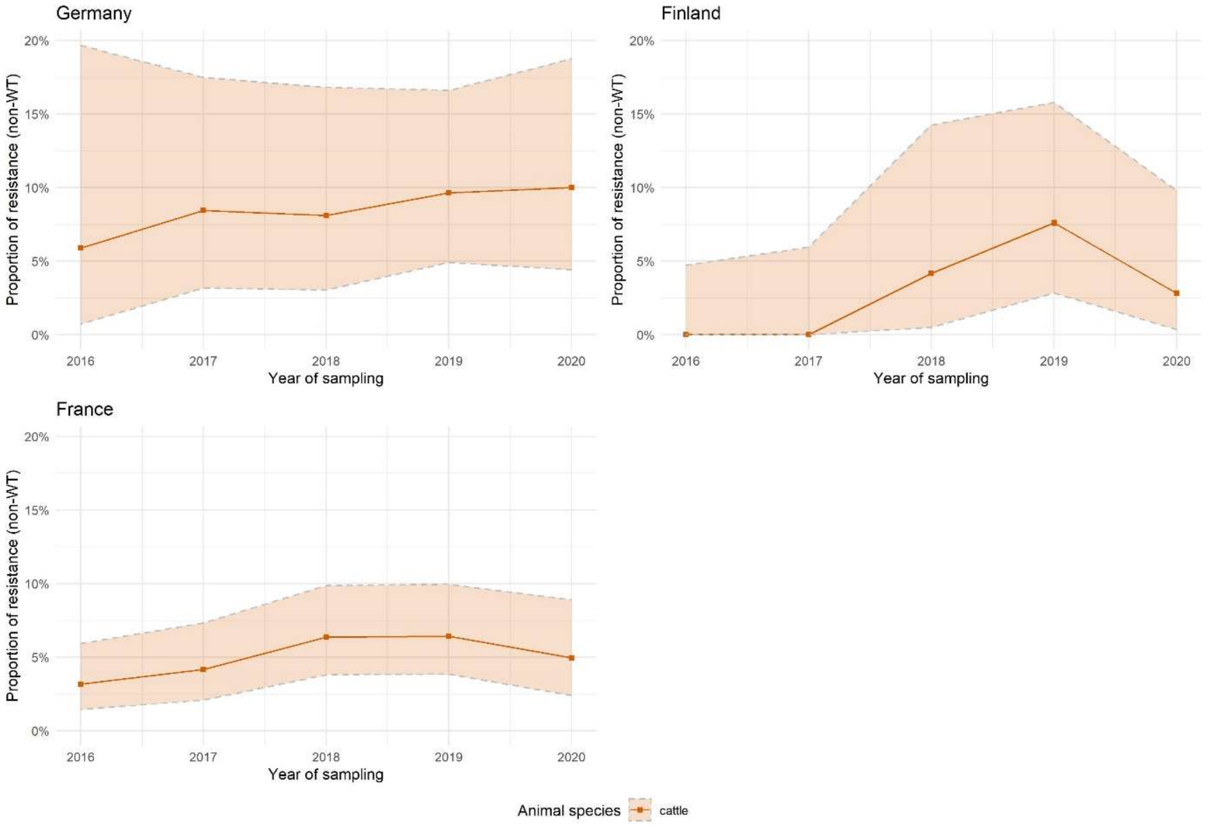

Figure S20. Trends of *Mannheimia haemolytica* resistance (non-wild-type) to aminopenicillins over 2016-2020

Only countries and animal species with sufficient data (at least 30 isolates per animal species and per year) are displayed on the figures.

Coloured areas around the curves represent 95% confidence intervals.

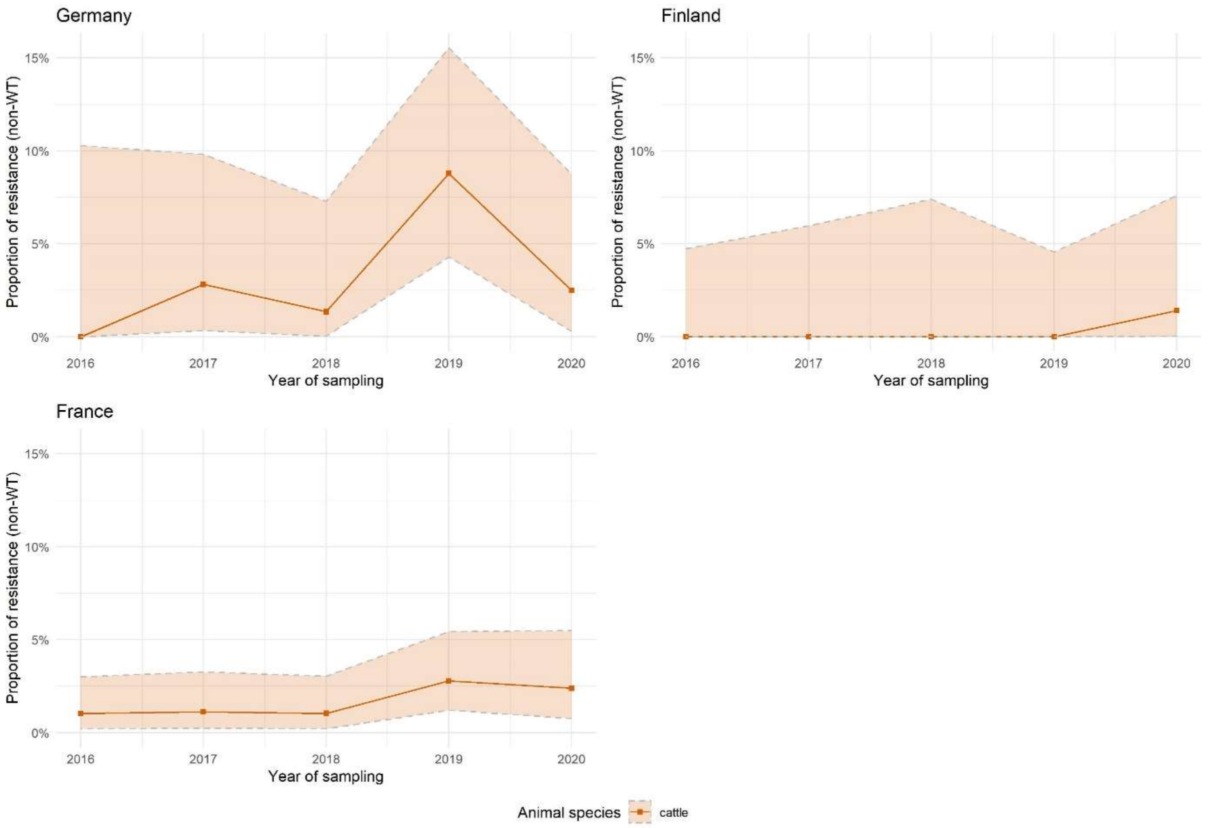

Figure S21. Trends of *Mannheimia haemolytica* resistance (non-wild-type) to amphenicols over 2016-2020

Only countries and animal species with sufficient data (at least 30 isolates per animal species and per year) are displayed on the figures.

Coloured areas around the curves represent 95% confidence intervals.

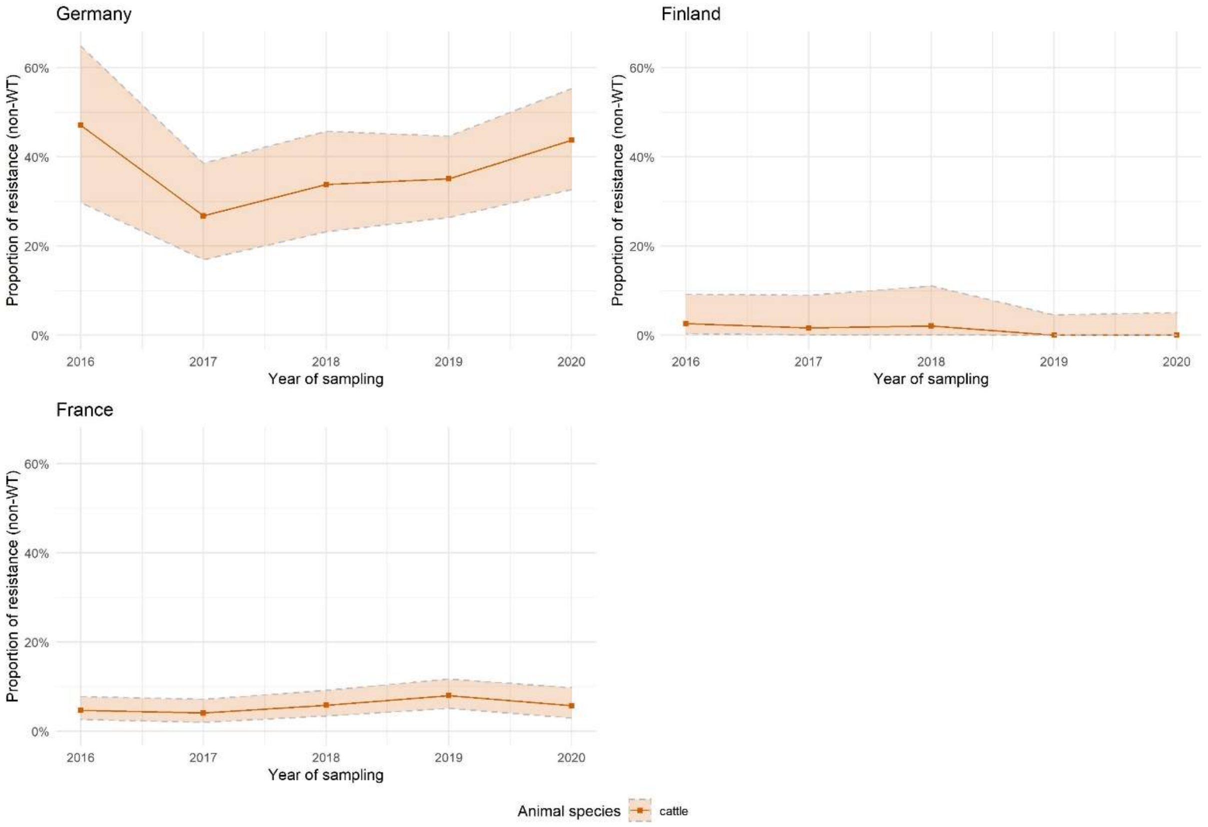

Figure S22. Trends of *Mannheimia haemolytica* resistance (non-wild-type) to fluoroquinolones over 2016-2020

Only countries and animal species with sufficient data (at least 30 isolates per animal species and per year) are displayed on the figures.

Coloured areas around the curves represent 95% confidence intervals.

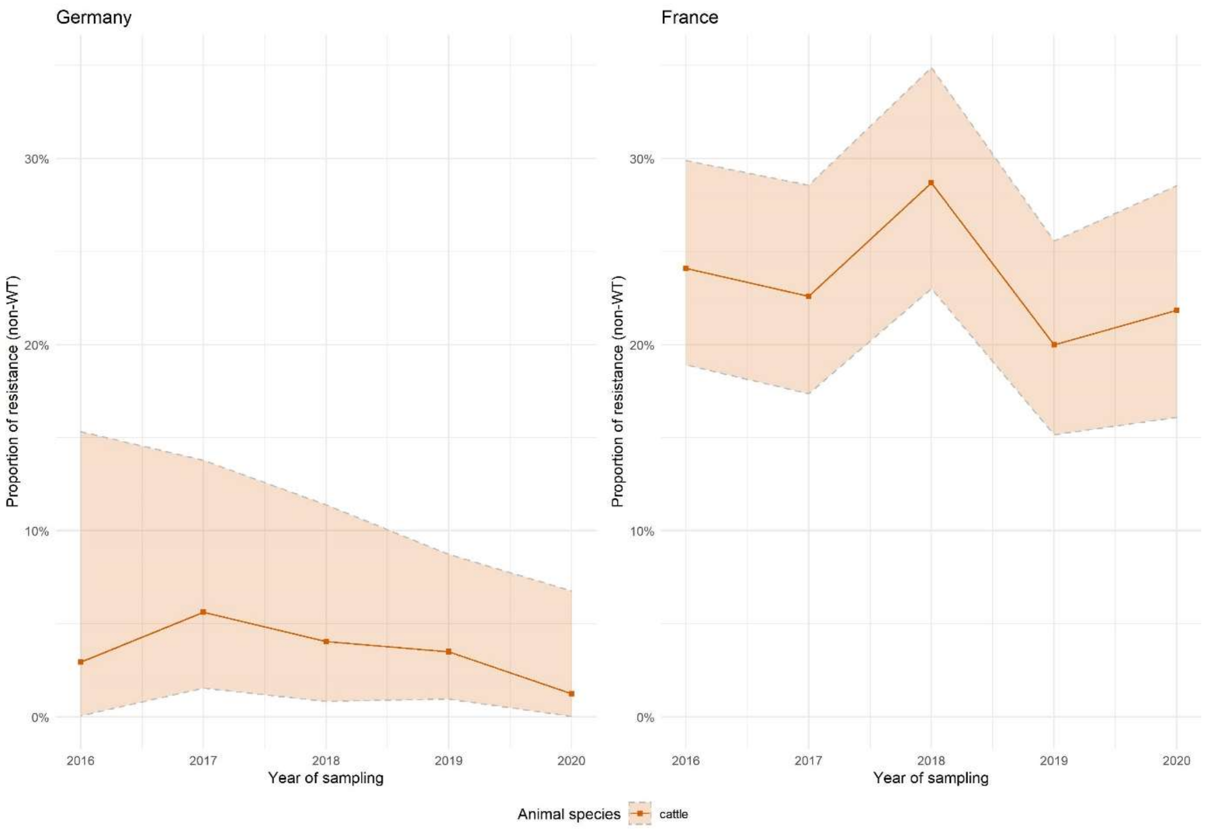

Figure S23. Trends of *Mannheimia haemolytica* resistance (non-wild-type) to gentamicin over 2016-2020

Only countries and animal species with sufficient data (at least 30 isolates per animal species and per year) are displayed on the figures.

Coloured areas around the curves represent 95% confidence intervals.

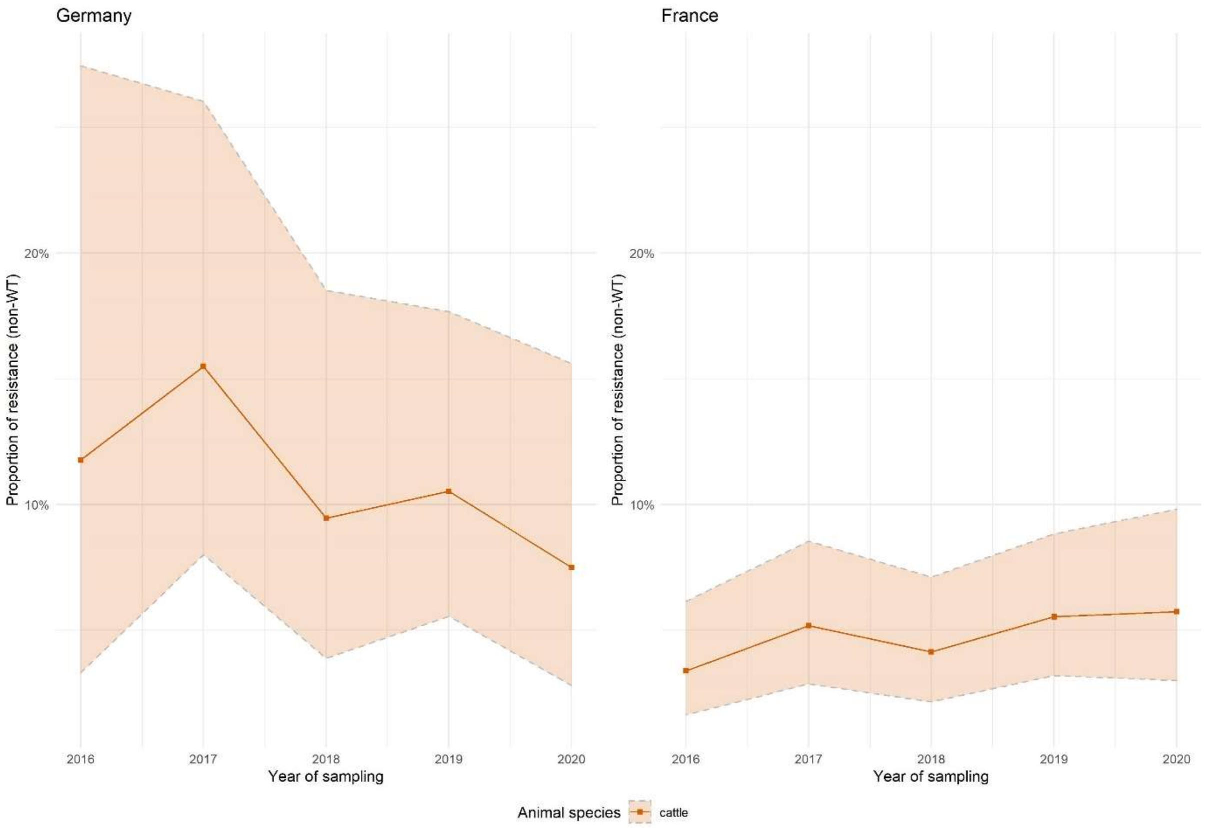

Figure S24. Trends of *Mannheimia haemolytica* resistance (non-wild-type) to sulfonamide-trimethoprim over 2016-2020

Only countries and animal species with sufficient data (at least 30 isolates per animal species and per year) are displayed on the figures.

Coloured areas around the curves represent 95% confidence intervals.

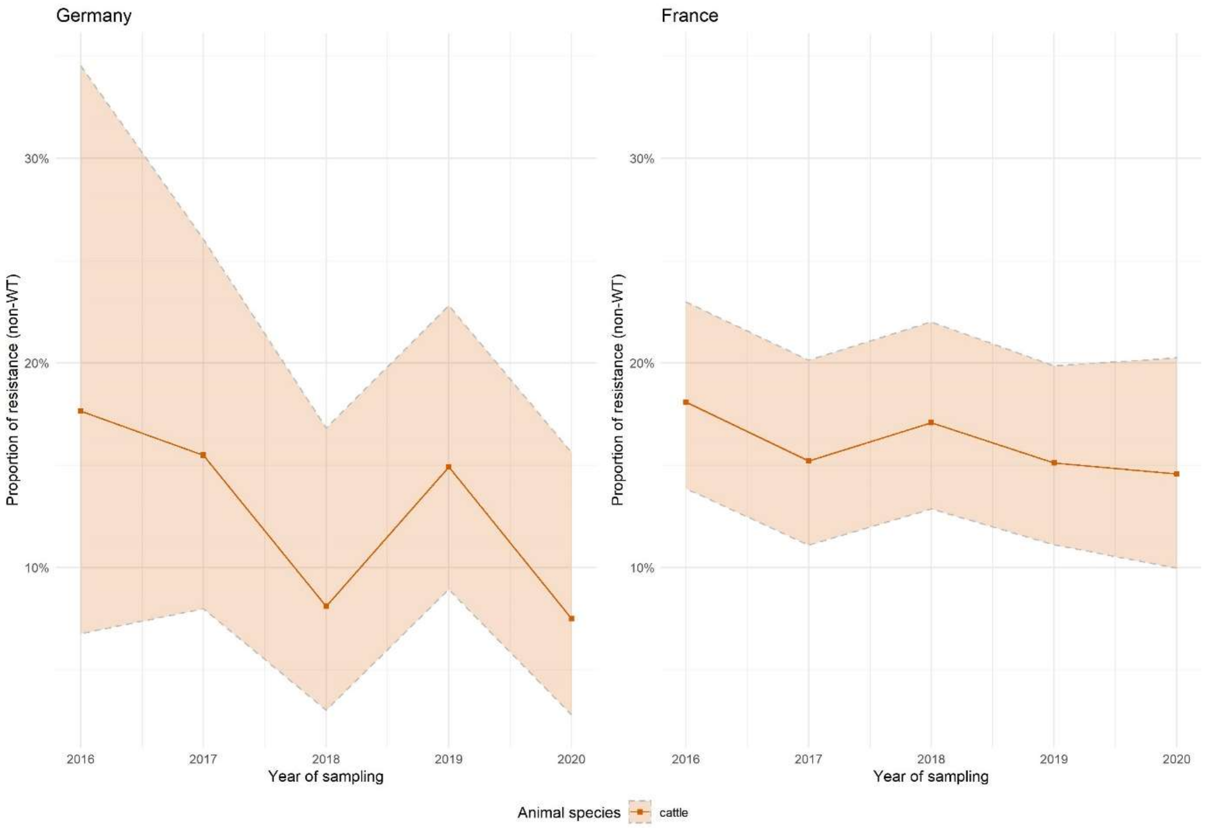

Figure S25. Trends of *Mannheimia haemolytica* resistance (non-wild-type) to tetracyclines over 2016-2020

Only countries and animal species with sufficient data (at least 30 isolates per animal species and per year) are displayed on the figures.

Coloured areas around the curves represent 95% confidence intervals.

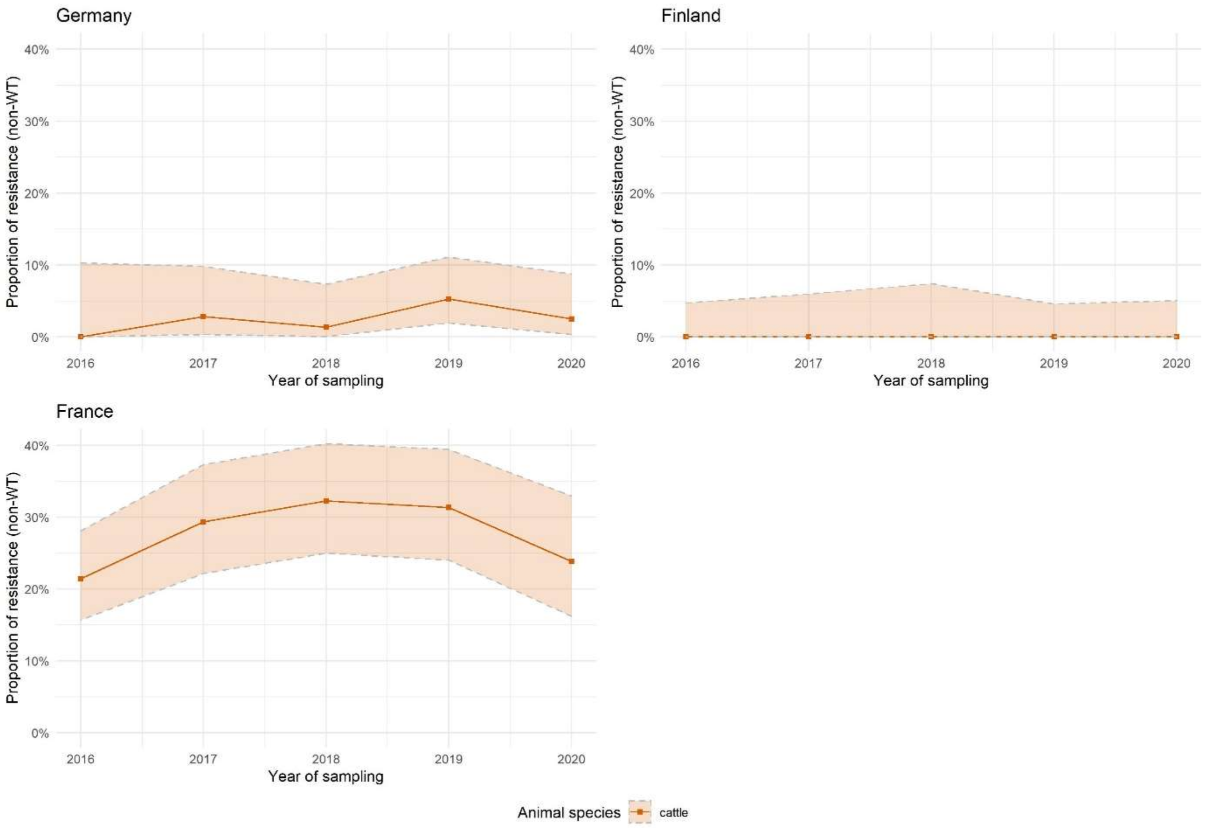

Figure S26. Trends of *Mannheimia haemolytica* resistance (non-wild-type) to tulathromycin over 2016-2020

Only countries and animal species with sufficient data (at least 30 isolates per animal species and per year) are displayed on the figures.

Coloured areas around the curves represent 95% confidence intervals.

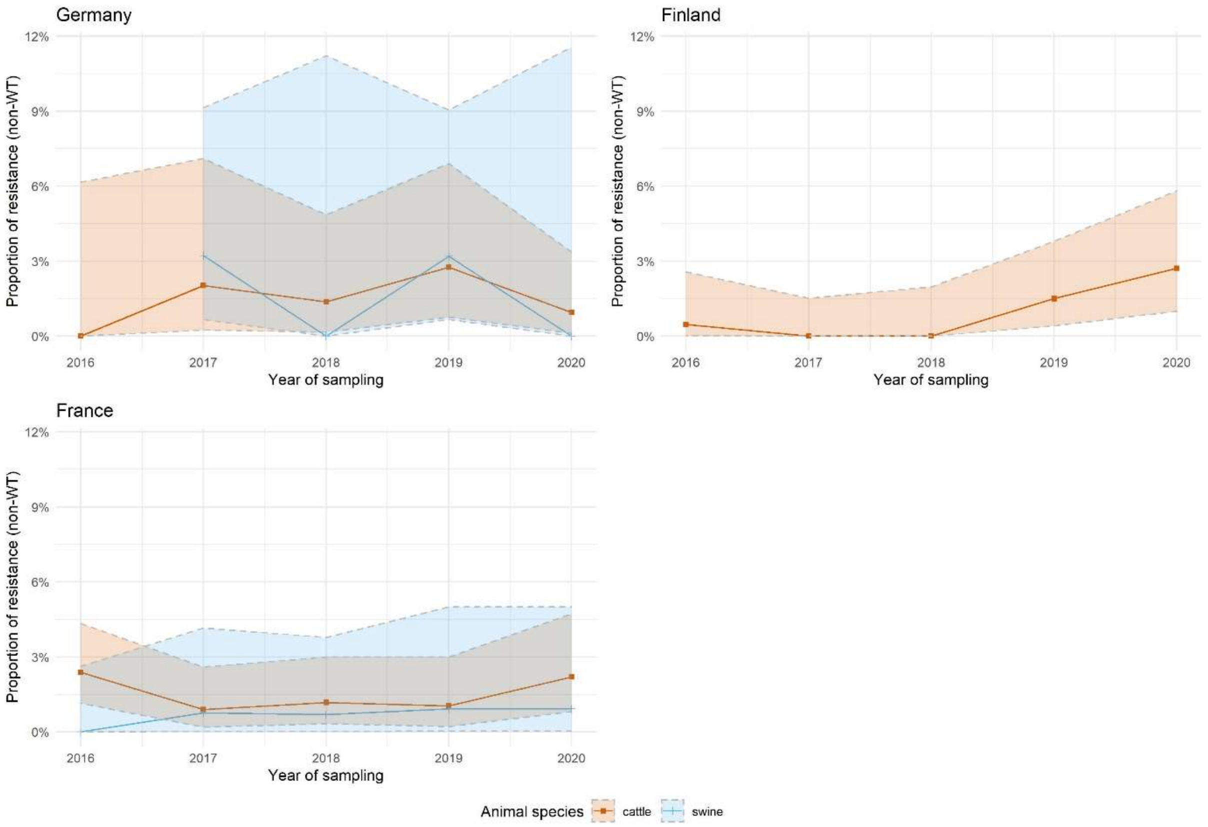

Figure S27. Trends of *Pasteurella multocida* resistance (non-wild-type) to aminopenicillins over 2016-2020

Only countries and animal species with sufficient data (at least 30 isolates per animal species and per year) are displayed on the figures.

Coloured areas around the curves represent 95% confidence intervals.

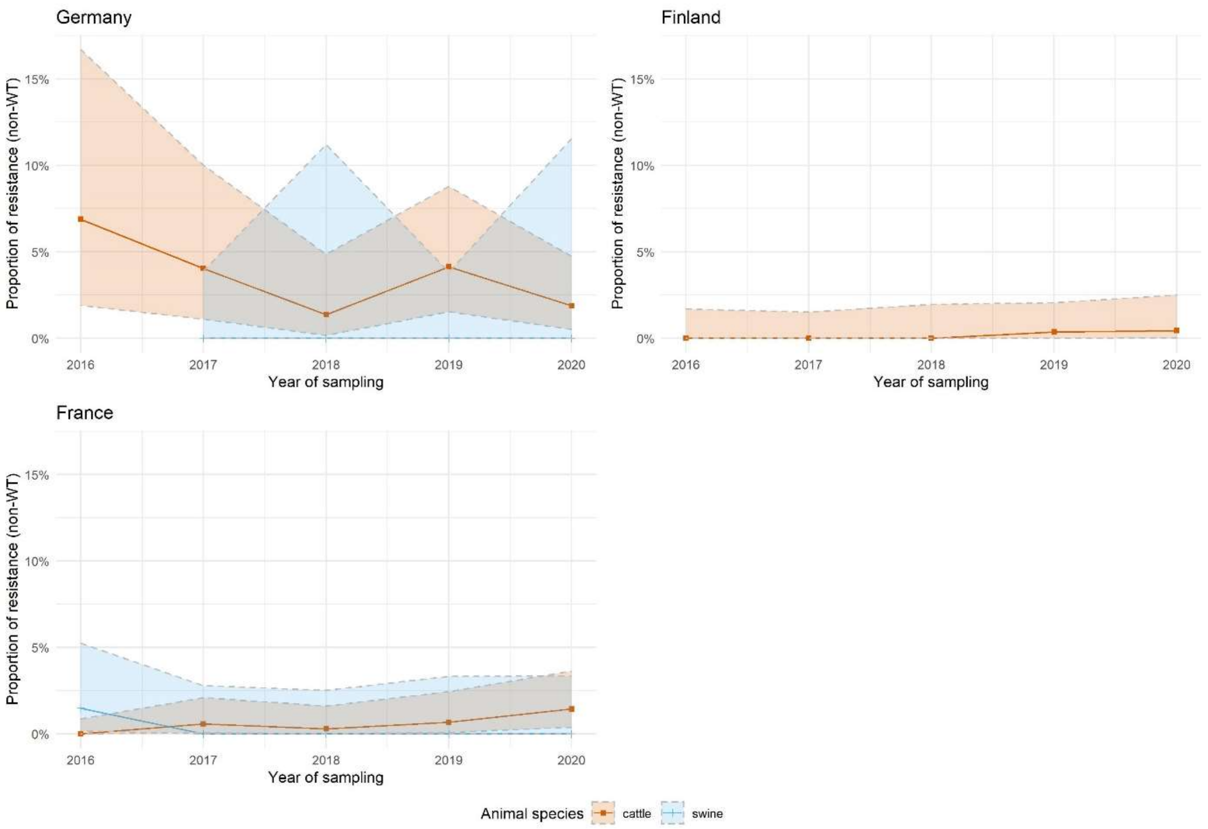

Figure S28. Trends of *Pasteurella multocida* resistance (non-wild-type) to amphenicols over 2016-2020

Only countries and animal species with sufficient data (at least 30 isolates per animal species and per year) are displayed on the figures.

Coloured areas around the curves represent 95% confidence intervals.

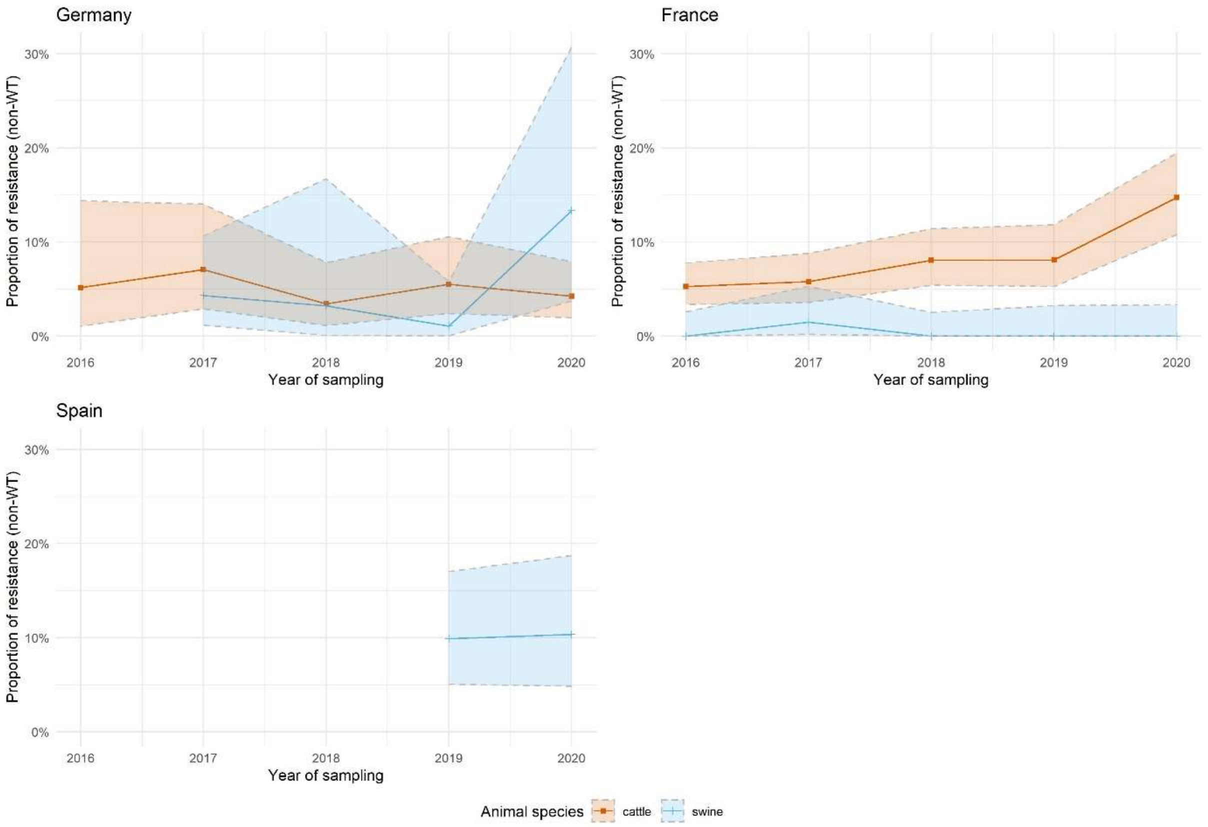

Figure S29. Trends of *Pasteurella multocida* resistance (non-wild-type) to fluoroquinolones over 2016-2020

Only countries and animal species with sufficient data (at least 30 isolates per animal species and per year) are displayed on the figures.

Coloured areas around the curves represent 95% confidence intervals.

Data from Spain (North-eastern Spain) are regional data, hence cannot be considered as representative from the entire country.

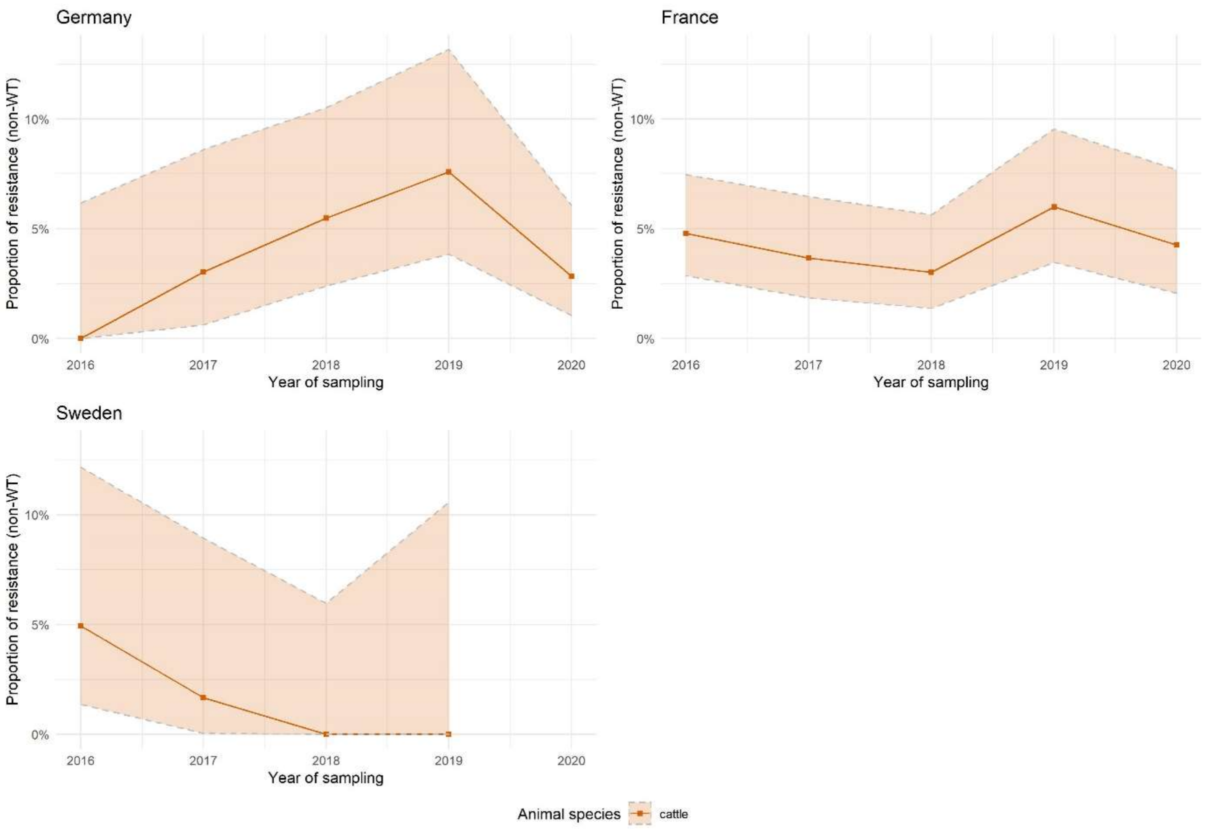

Figure S30. Trends of *Pasteurella multocida* resistance (non-wild-type) to gentamicin over 2016-2020

Only countries and animal species with sufficient data (at least 30 isolates per animal species and per year) are displayed on the figures.

Coloured areas around the curves represent 95% confidence intervals.

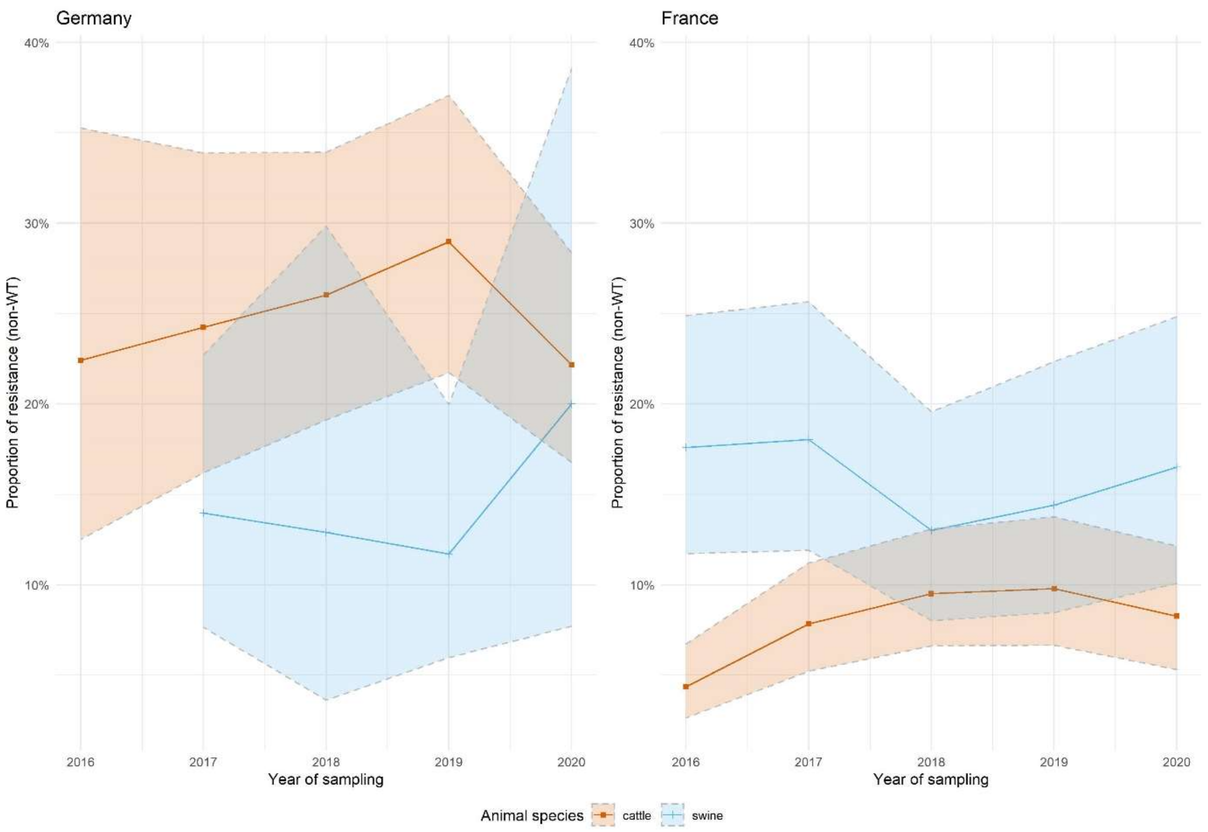

Figure S31. Trends of *Pasteurella multocida* resistance (non-wild-type) to sulfonamide-trimethoprim over 2016-2020

Only countries and animal species with sufficient data (at least 30 isolates per animal species and per year) are displayed on the figures.

Coloured areas around the curves represent 95% confidence intervals.

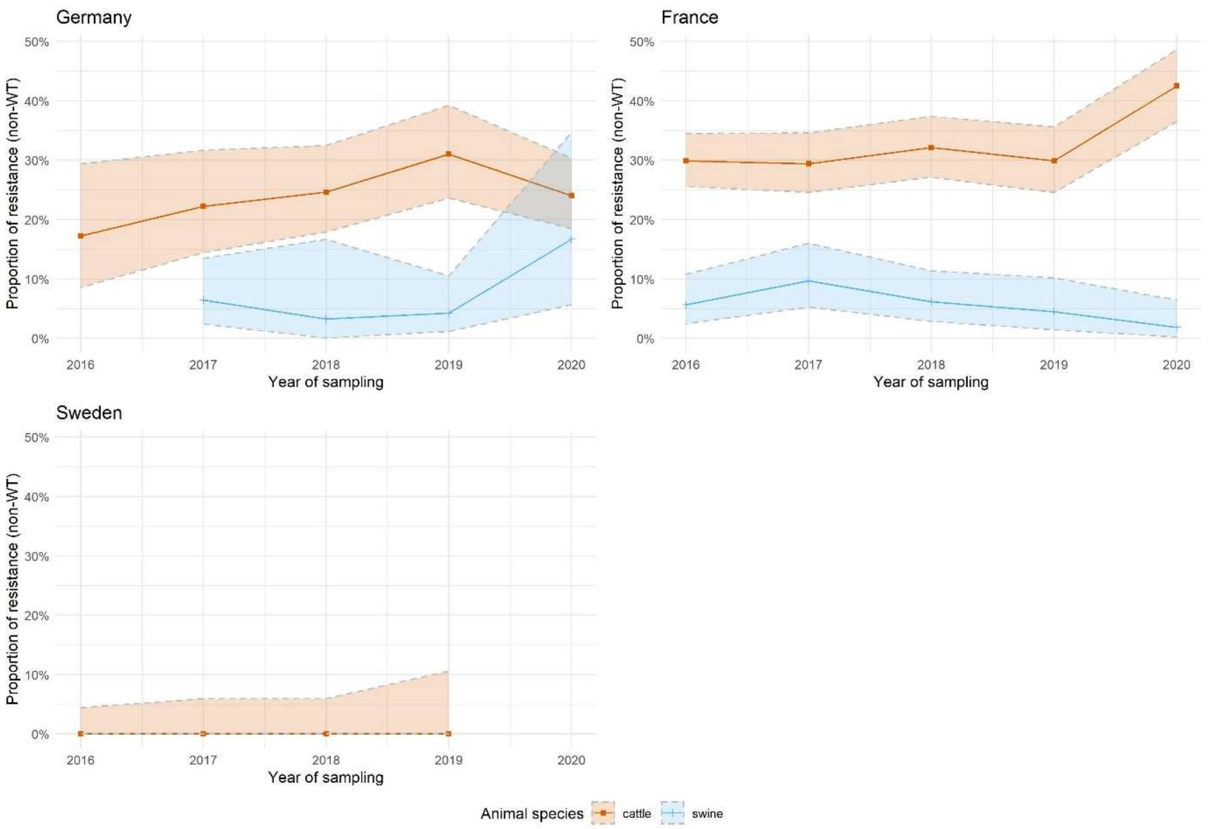

Figure S32. Trends of *Pasteurella multocida* resistance (non-wild-type) to tetracyclines over 2016-2020

Only countries and animal species with sufficient data (at least 30 isolates per animal species and per year) are displayed on the figures.

Coloured areas around the curves represent 95% confidence intervals.

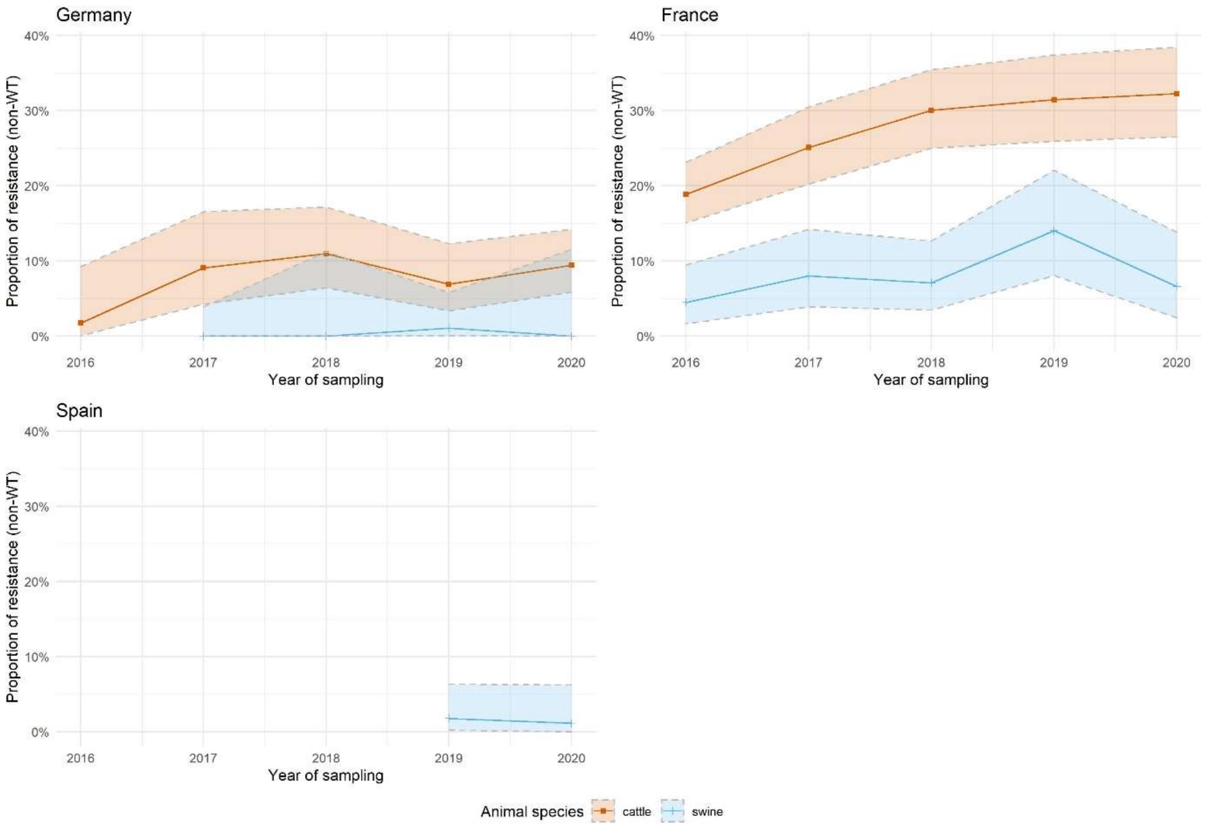

Figure S33. Trends of *Pasteurella multocida* resistance (non-wild-type) to tilmicosin over 2016-2020

Only countries and animal species with sufficient data (at least 30 isolates per animal species and per year) are displayed on the figures.

Coloured areas around the curves represent 95% confidence intervals.

Data from Spain (North-eastern Spain) are regional data, hence cannot be considered as representative from the entire country.

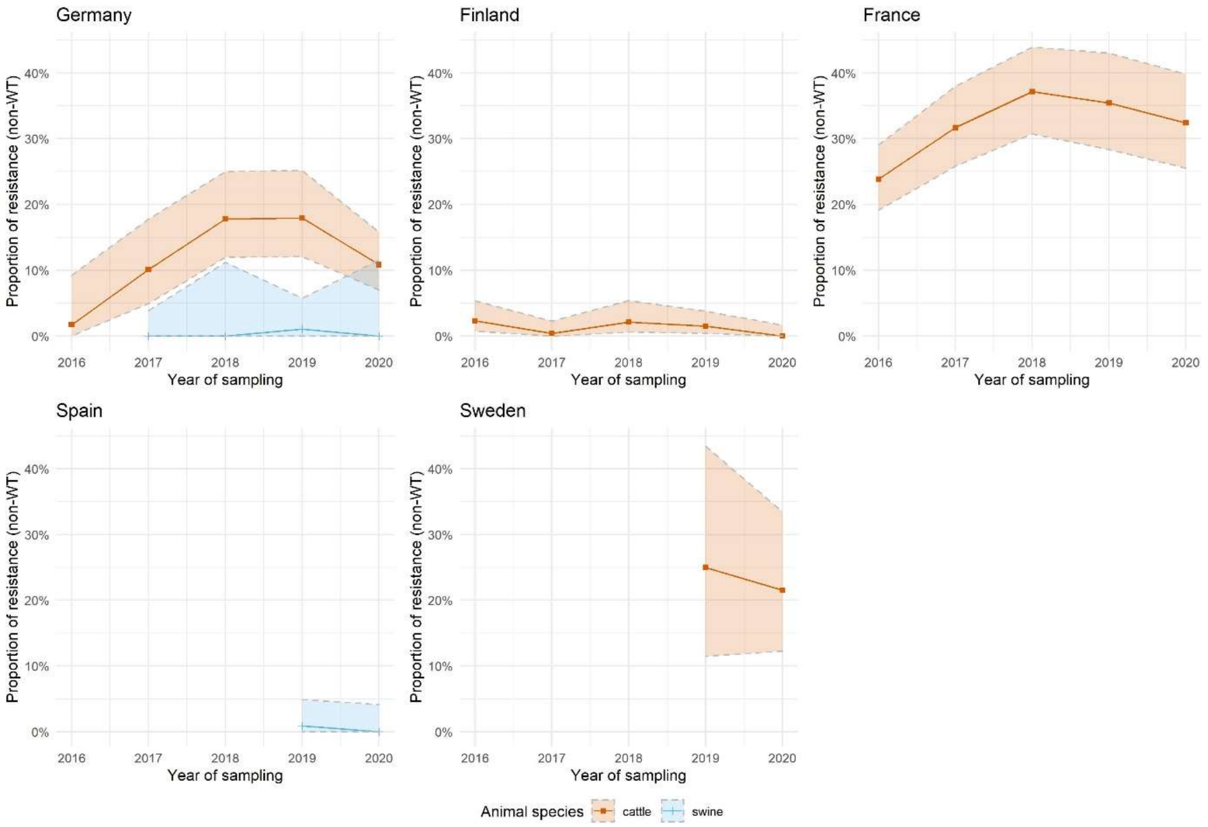

Figure S34. Trends of *Pasteurella multocida* resistance (non-wild-type) to tulathromycin over 2016-2020

Only countries and animal species with sufficient data (at least 30 isolates per animal species and per year) are displayed on the figures.

Coloured areas around the curves represent 95% confidence intervals.

Data from Spain (North-eastern Spain) are regional data, hence cannot be considered as representative from the entire country.

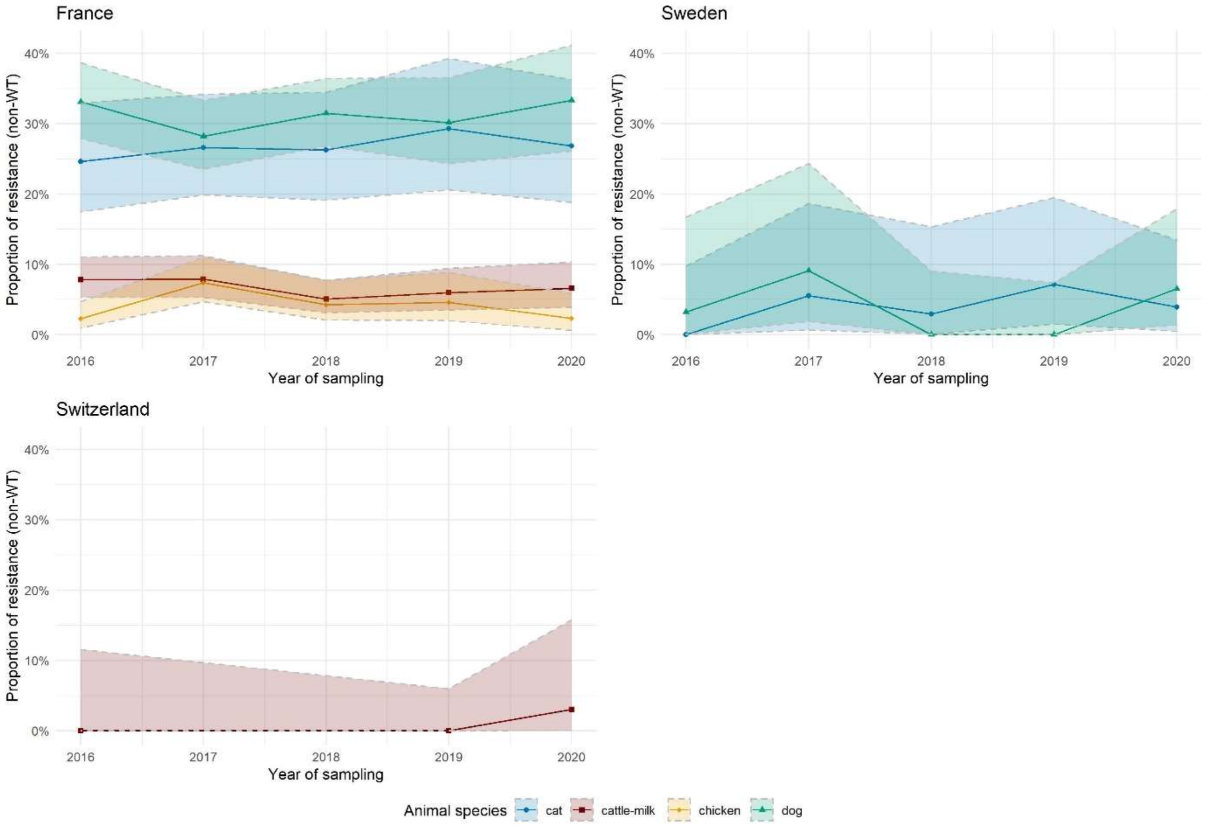

Figure S35. Trends of *Staphylococcus aureus* resistance (non-wild-type) to erythromycin over 2016-2020

Only countries and animal species with sufficient data (at least 30 isolates per animal species and per year) are displayed on the figures.

Coloured areas around the curves represent 95% confidence intervals.

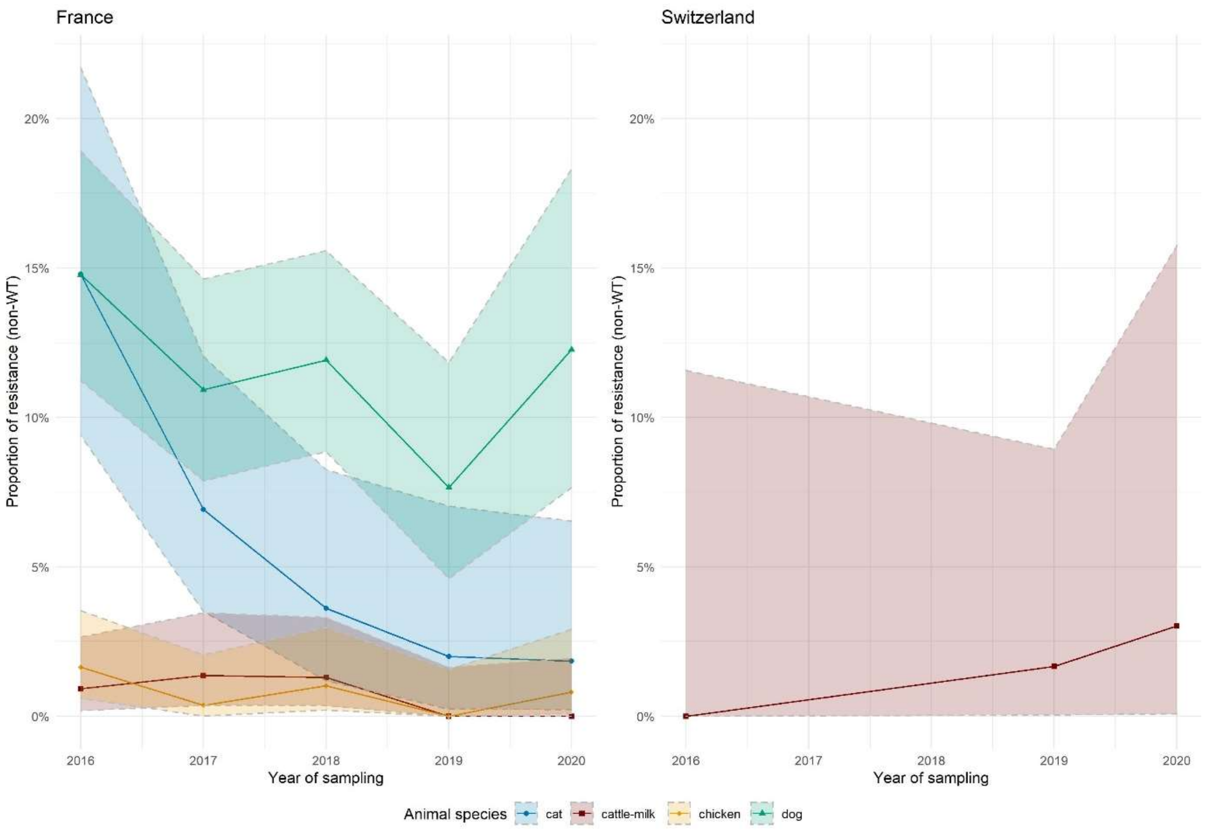

Figure S36. Trends of *Staphylococcus aureus* resistance (non-wild-type) to fluoroquinolones over 2016-2020

Only countries and animal species with sufficient data (at least 30 isolates per animal species and per year) are displayed on the figures.

Coloured areas around the curves represent 95% confidence intervals.

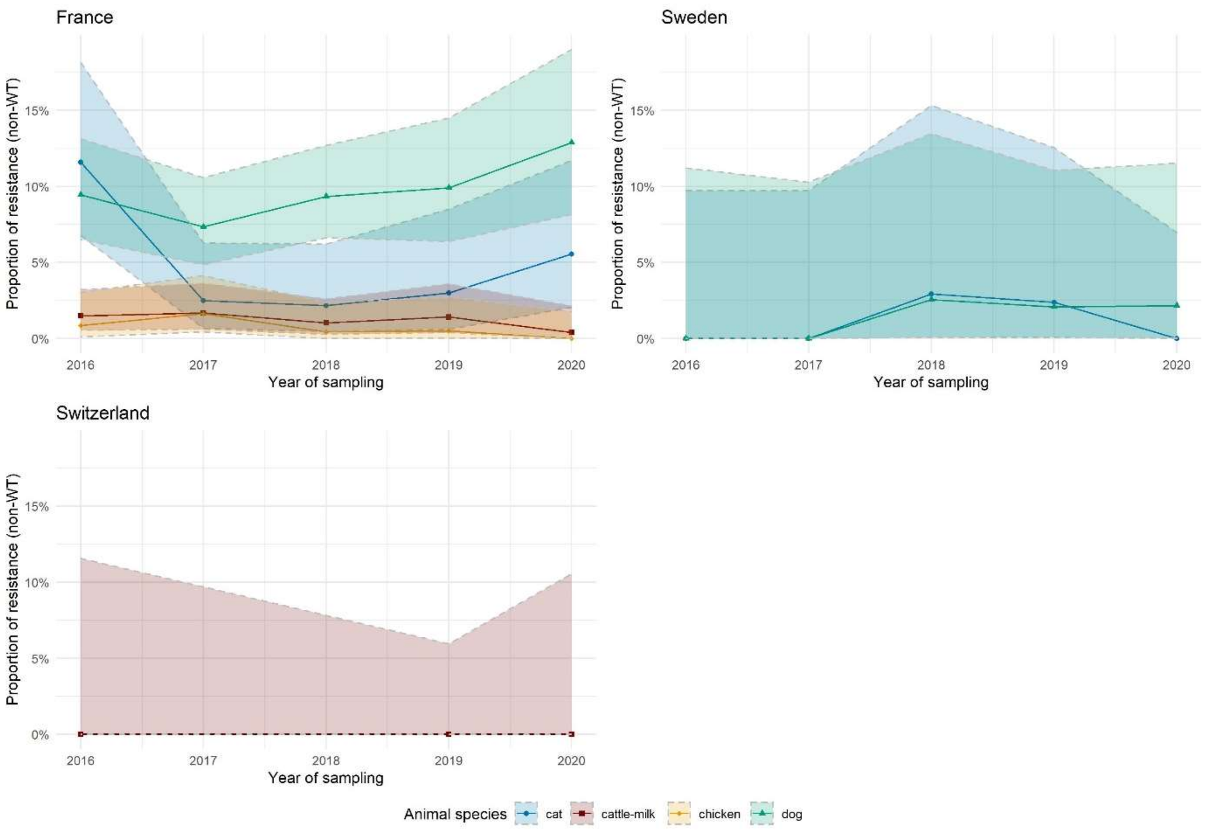

Figure S37. Trends of *Staphylococcus aureus* resistance (non-wild-type) to gentamicin over 2016-2020

Only countries and animal species with sufficient data (at least 30 isolates per animal species and per year) are displayed on the figures.

Coloured areas around the curves represent 95% confidence intervals.

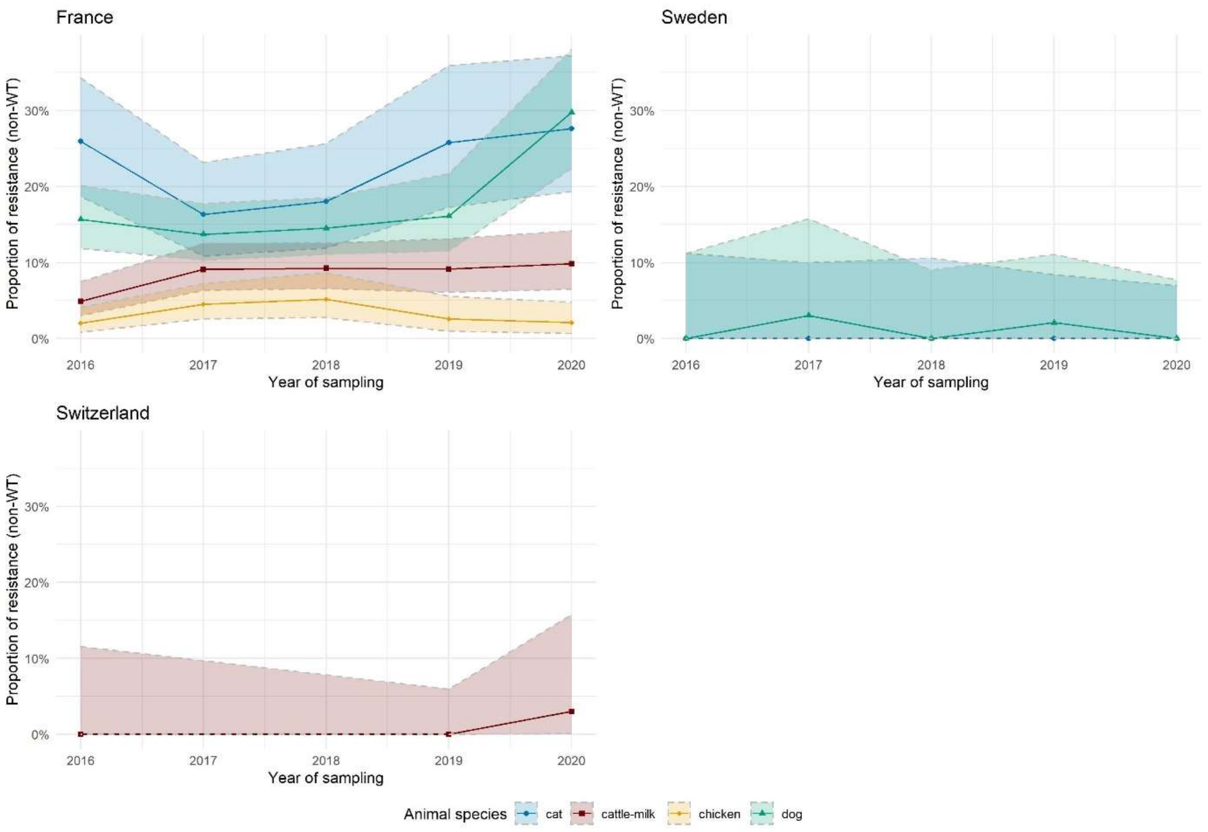

Figure S38. Trends of *Staphylococcus aureus* resistance (non-wild-type) to methicillin over 2016-2020

Only countries and animal species with sufficient data (at least 30 isolates per animal species and per year) are displayed on the figures.

Coloured areas around the curves represent 95% confidence intervals.

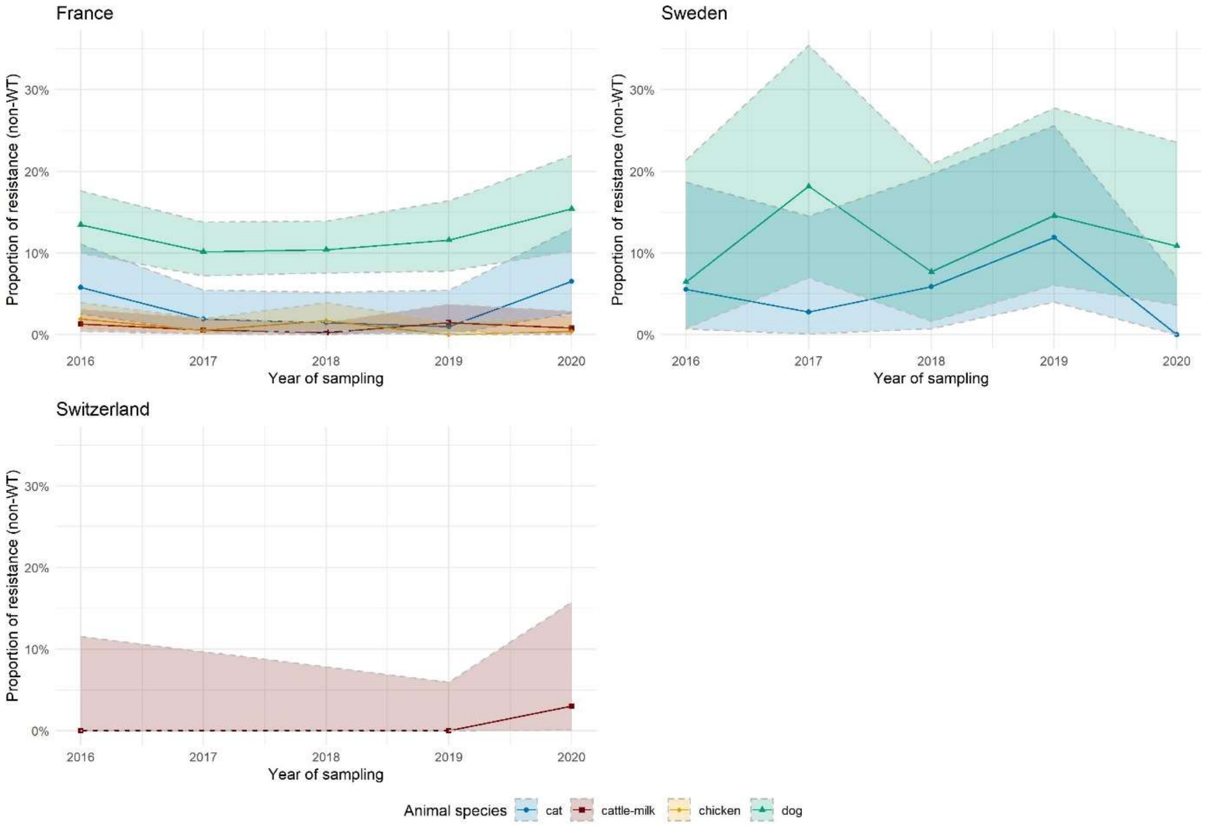

Figure S39. Trends of *Staphylococcus aureus* resistance (non-wild-type) to sulfonamide-trimethoprim over 2016-2020

Only countries and animal species with sufficient data (at least 30 isolates per animal species and per year) are displayed on the figures.

Coloured areas around the curves represent 95% confidence intervals.

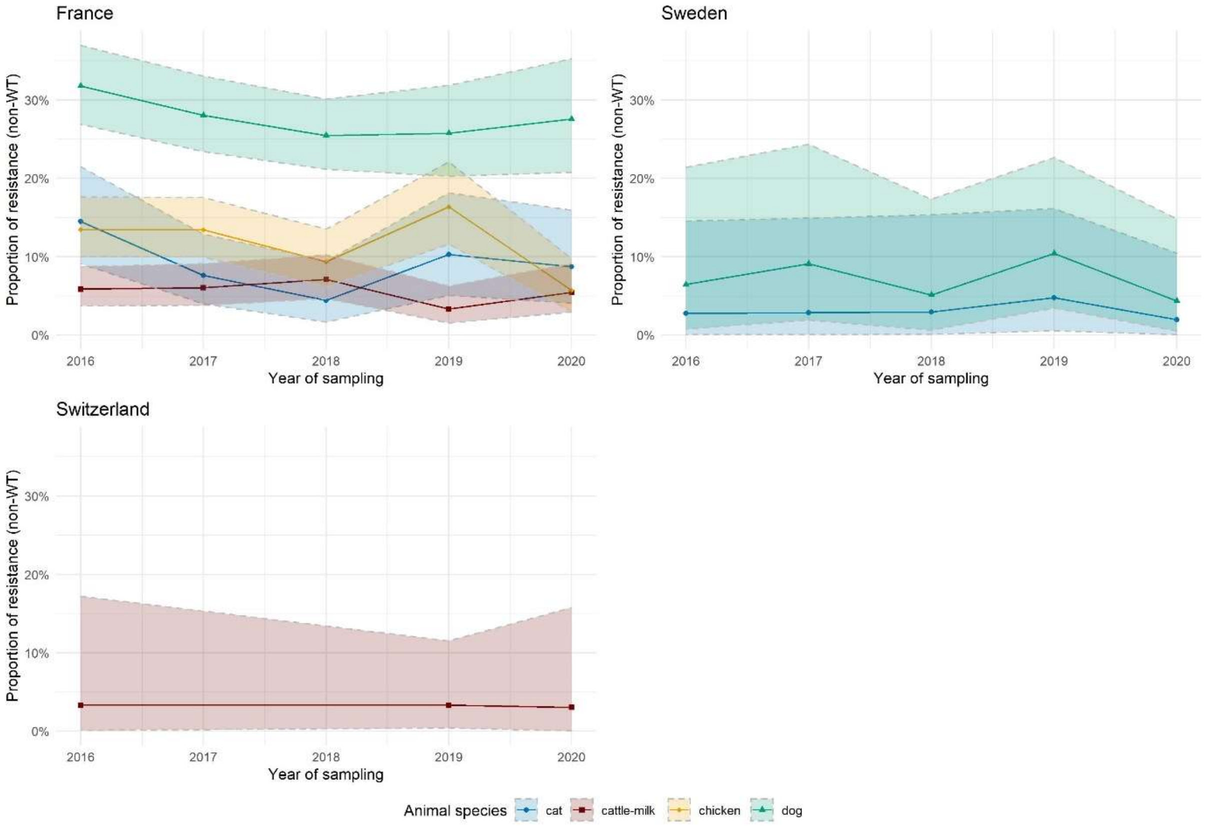

Figure S40. Trends of *Staphylococcus aureus* resistance (non-wild-type) to tetracyclines over 2016-2020

Only countries and animal species with sufficient data (at least 30 isolates per animal species and per year) are displayed on the figures.

Coloured areas around the curves represent 95% confidence intervals.

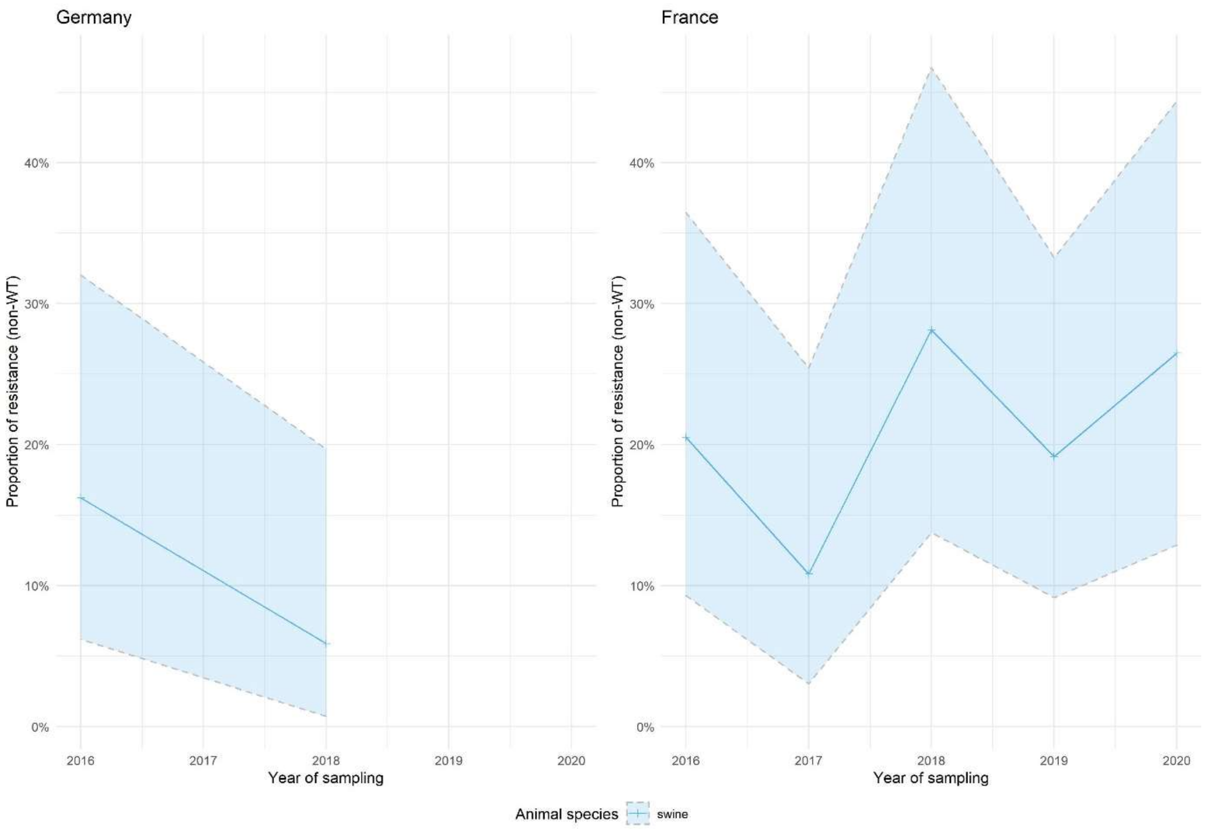

Figure S41. Trends of *Staphylococcus hyicus* resistance (non-wild-type) to erythromycin over 2016-2020

Only countries and animal species with sufficient data (at least 30 isolates per animal species and per year) are displayed on the figures.

Coloured areas around the curves represent 95% confidence intervals.

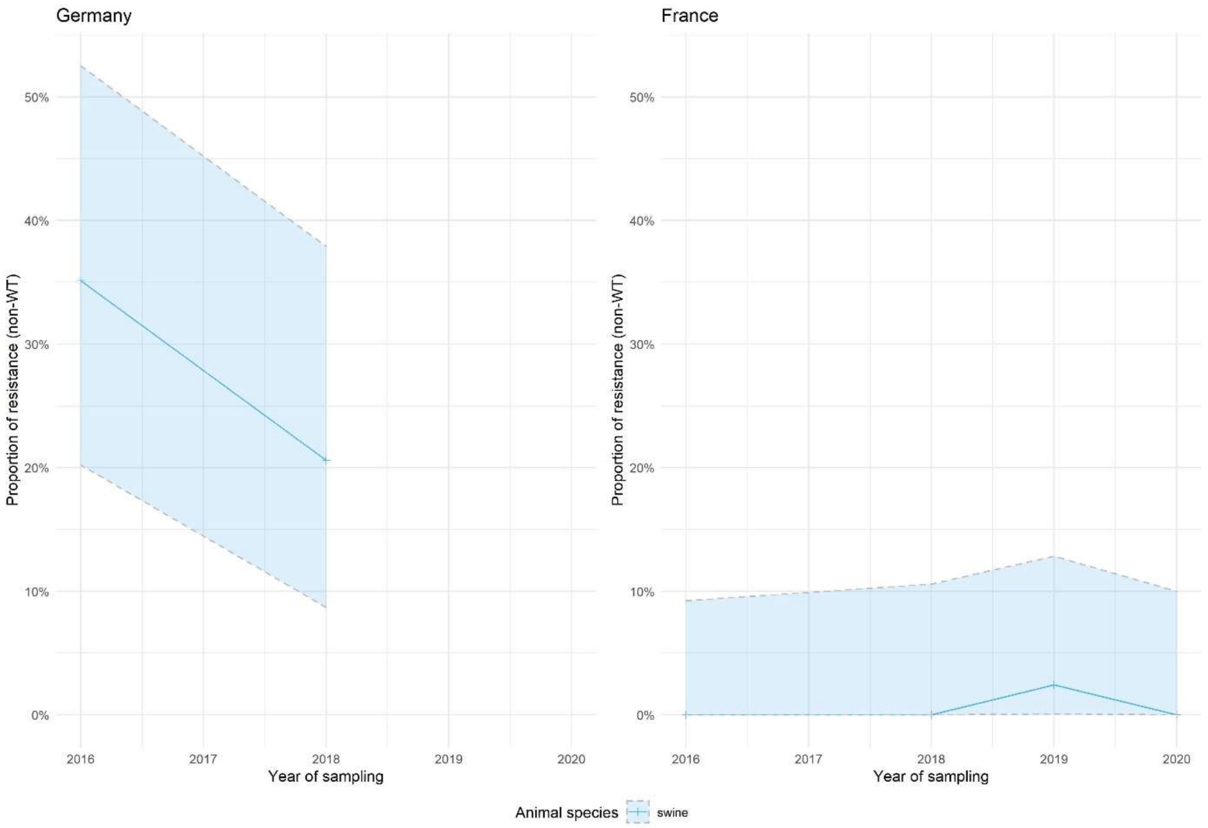

Figure S42. Trends of *Staphylococcus hyicus* resistance (non-wild-type) to fluoroquinolones over 2016-2020

Only countries and animal species with sufficient data (at least 30 isolates per animal species and per year) are displayed on the figures.

Coloured areas around the curves represent 95% confidence intervals.

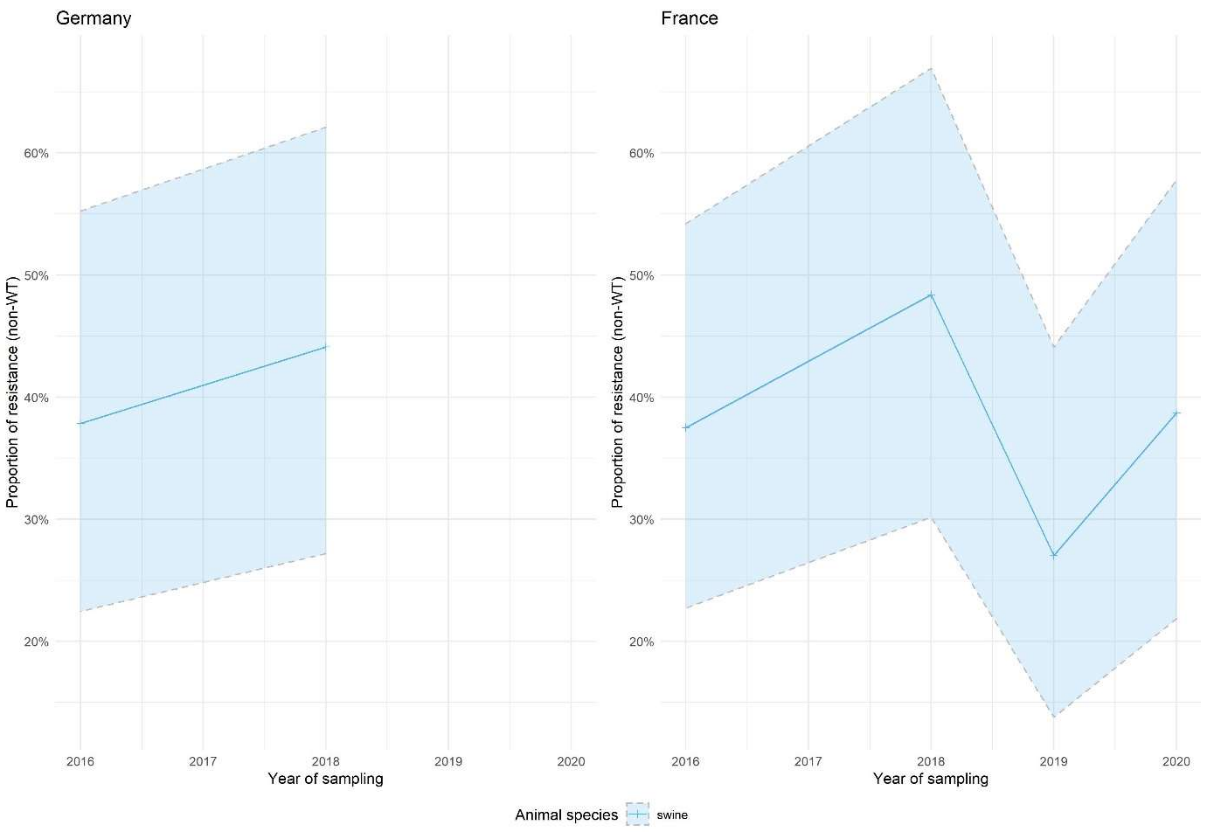

Figure S43. Trends of *Staphylococcus hyicus* resistance (non-wild-type) to tetracyclines over 2016-2020

Only countries and animal species with sufficient data (at least 30 isolates per animal species and per year) are displayed on the figures.

Coloured areas around the curves represent 95% confidence intervals.

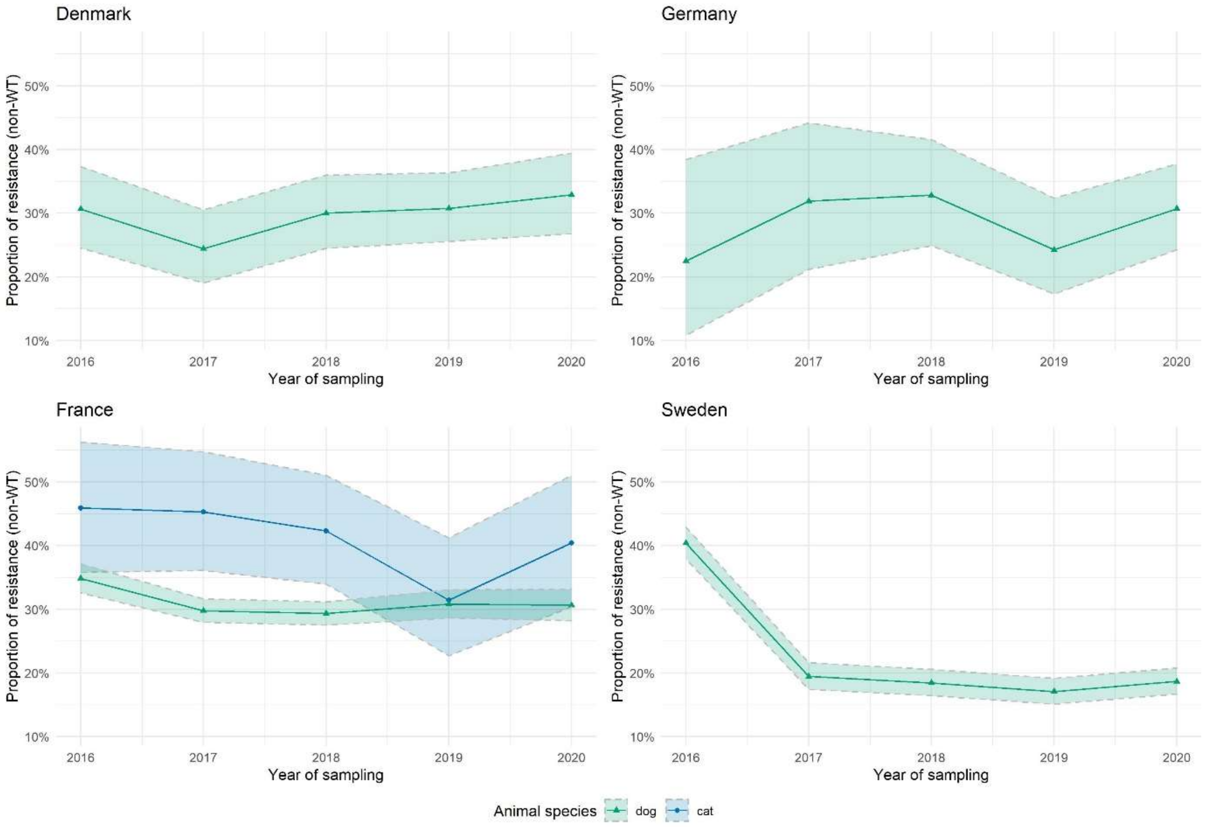

Figure S44. Trends of *Staphylococcus pseudintermedius* resistance (non-wild-type) to erythromycin over 2016-2020

Only countries and animal species with sufficient data (at least 30 isolates per animal species and per year) are displayed on the figures.

Coloured areas around the curves represent 95% confidence intervals.

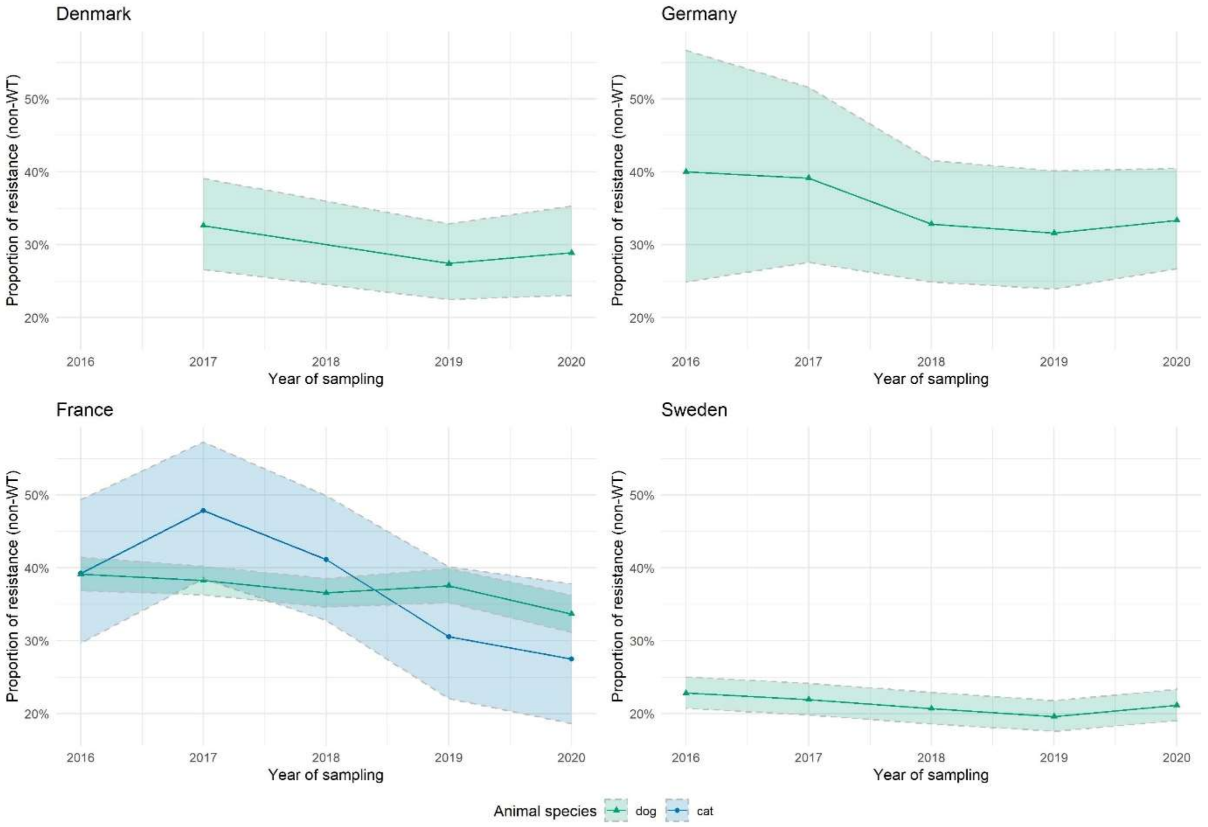

Figure S45. Trends of *Staphylococcus pseudintermedius* resistance (non-wild-type) to tetracyclines over 2016-2020

Only countries and animal species with sufficient data (at least 30 isolates per animal species and per year) are displayed on the figures.

Coloured areas around the curves represent 95% confidence intervals.

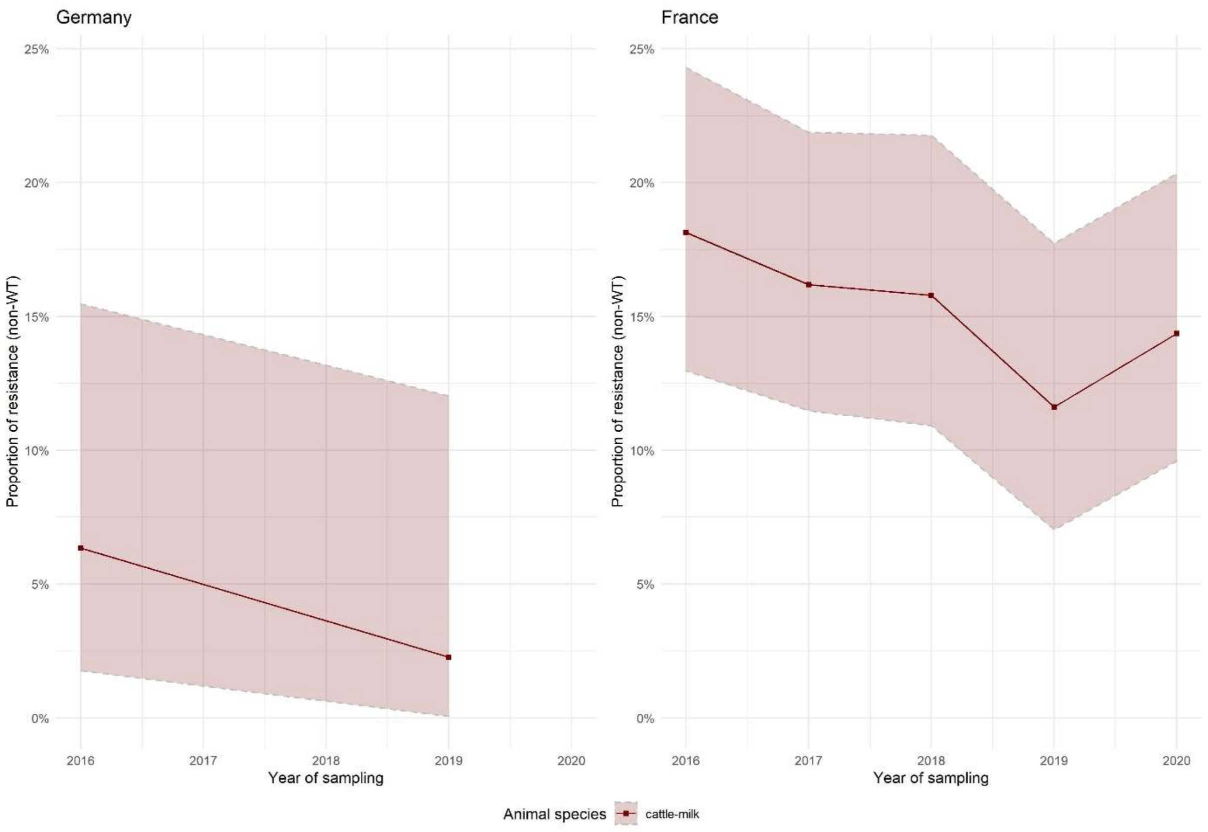

Figure S46. Trends of *Streptococcus dysgalactiae* resistance (non-wild-type) to erythromycin over 2016-2020

Only countries and animal species with sufficient data (at least 30 isolates per animal species and per year) are displayed on the figures.

Coloured areas around the curves represent 95% confidence intervals.

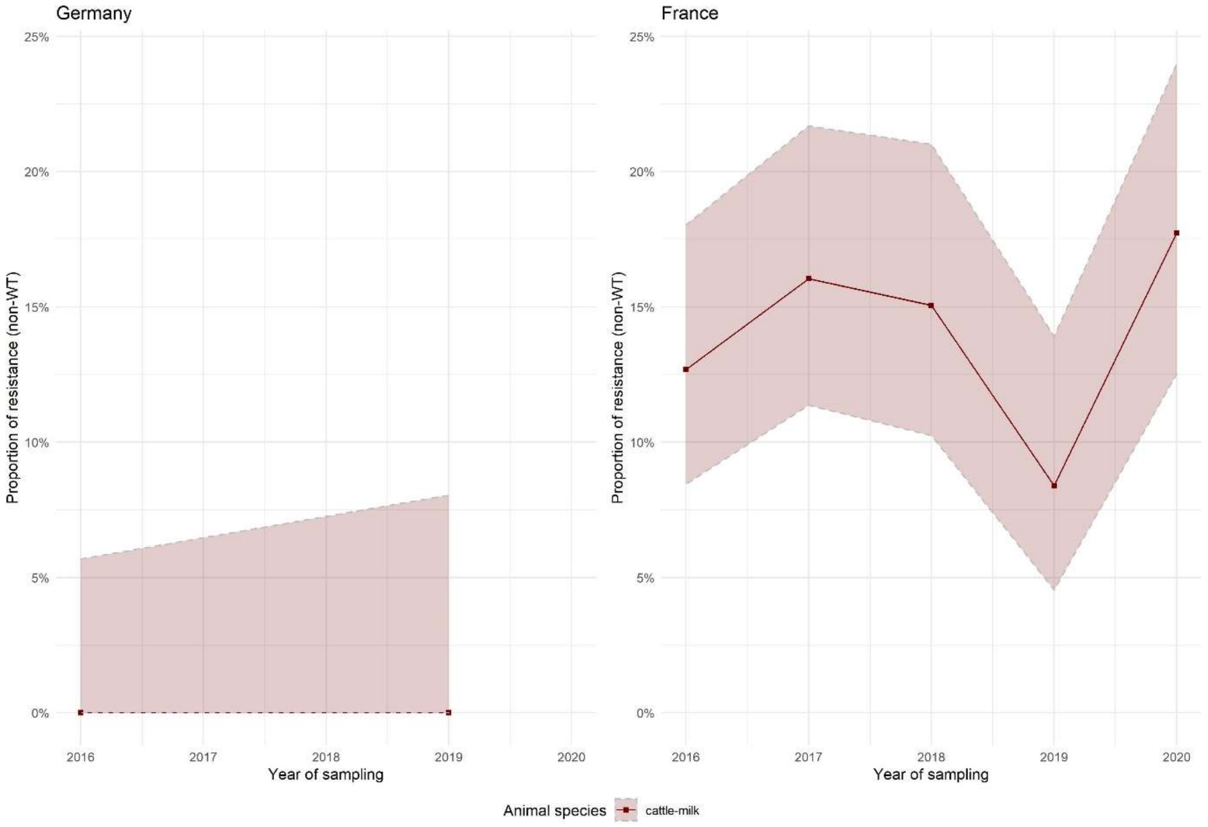

Figure S47. Trends of *Streptococcus dysgalactiae* resistance (non-wild-type) to sulfonamide-trimethoprim over 2016-2020

Only countries and animal species with sufficient data (at least 30 isolates per animal species and per year) are displayed on the figures.

Coloured areas around the curves represent 95% confidence intervals.

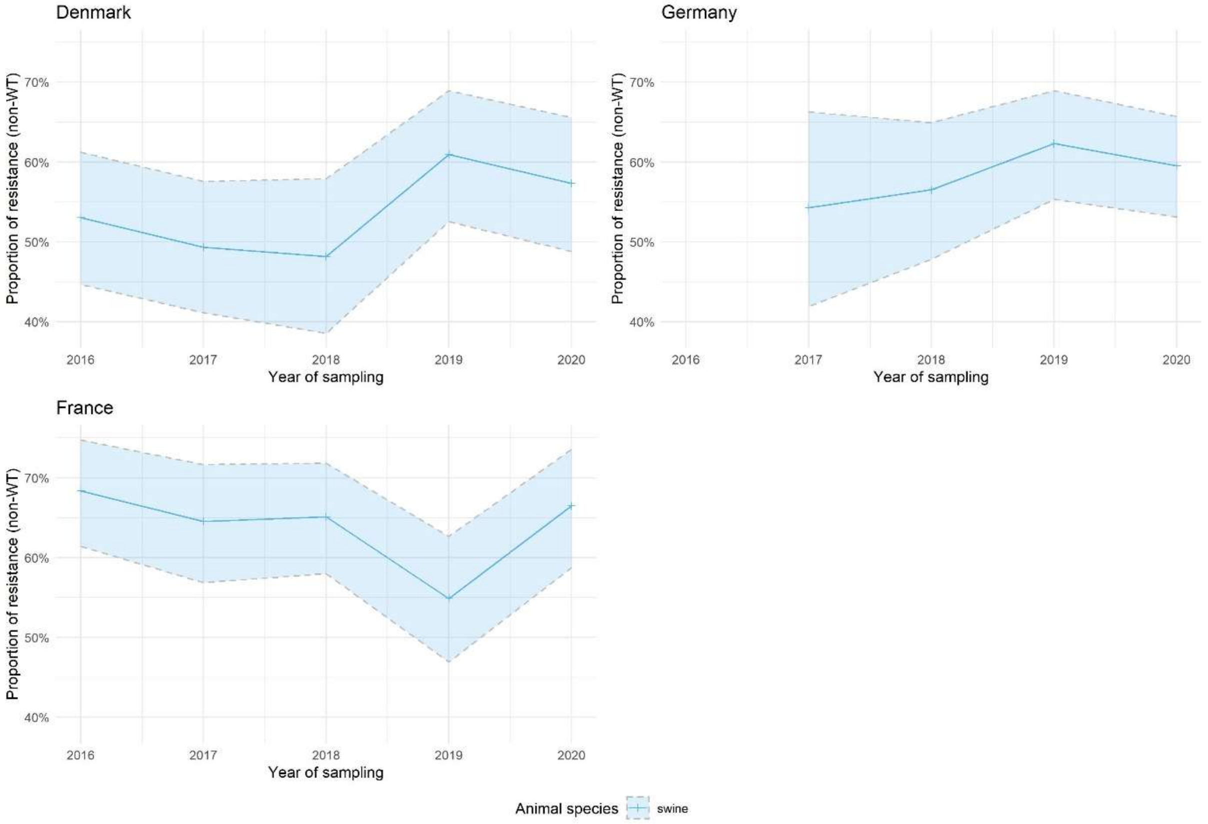

Figure S48. Trends of *Streptococcus suis* resistance (non-wild-type) to erythromycin over 2016-2020

Only countries and animal species with sufficient data (at least 30 isolates per animal species and per year) are displayed on the figures.

Coloured areas around the curves represent 95% confidence intervals.

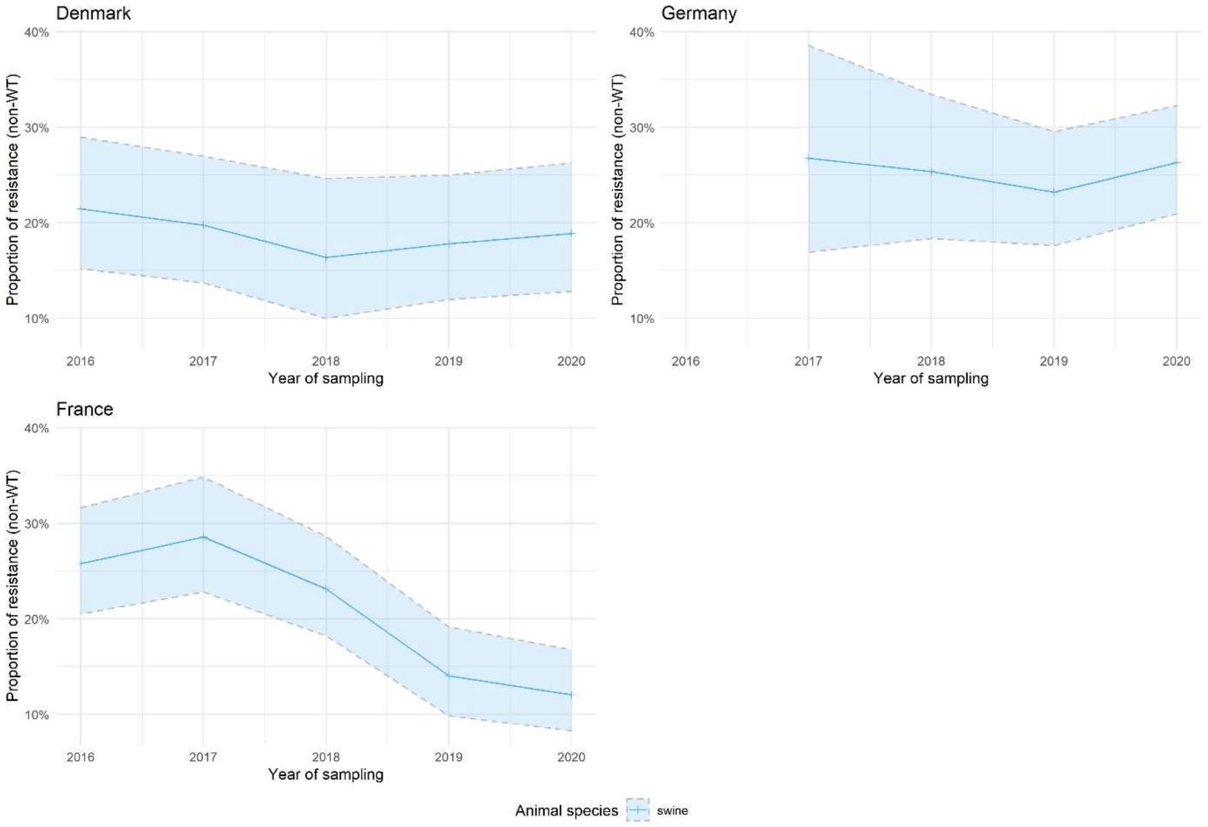

Figure S49. Trends of *Streptococcus suis* resistance (non-wild-type) to sulfonamide-trimethoprim over 2016-2020

Only countries and animal species with sufficient data (at least 30 isolates per animal species and per year) are displayed on the figures.

Coloured areas around the curves represent 95% confidence intervals.

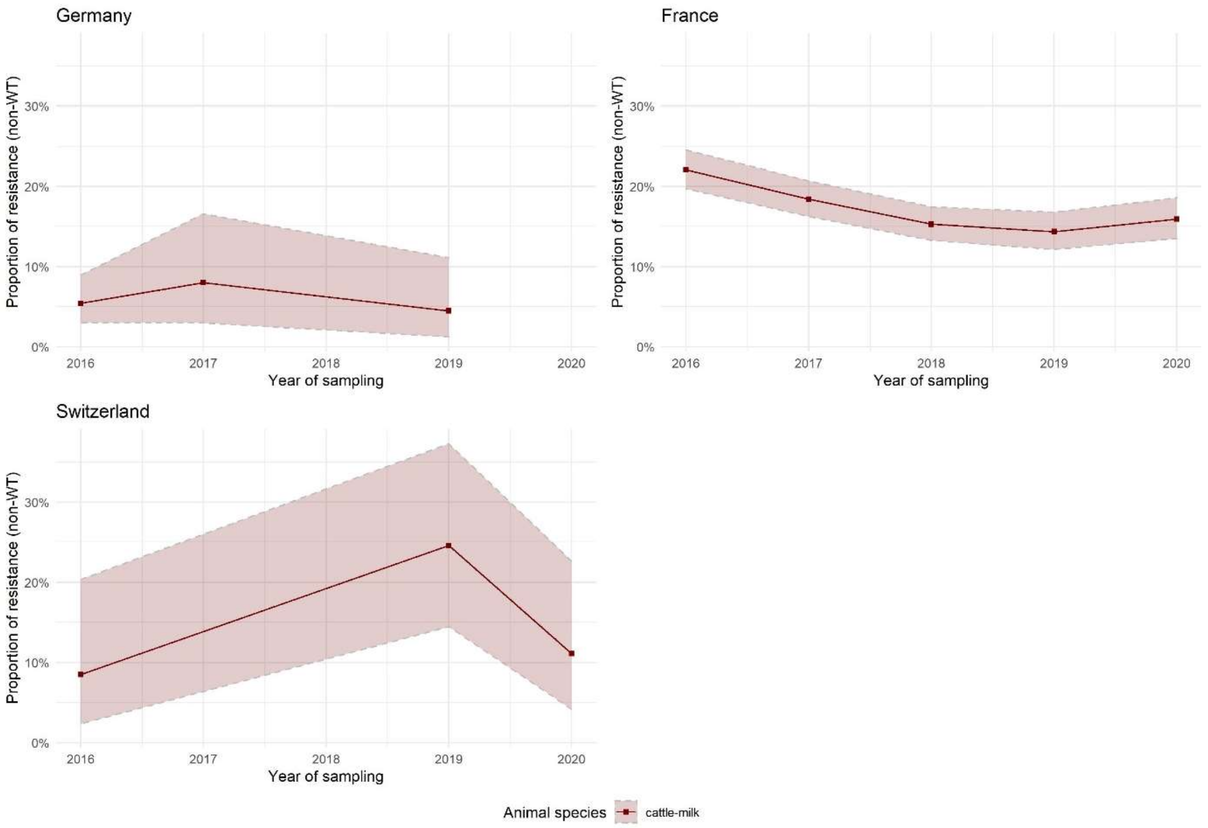

Figure S50. Trends of *Streptococcus uberis* resistance (non-wild-type) to erythromycin over 2016-2020

Only countries and animal species with sufficient data (at least 30 isolates per animal species and per year) are displayed on the figures.

Coloured areas around the curves represent 95% confidence intervals.

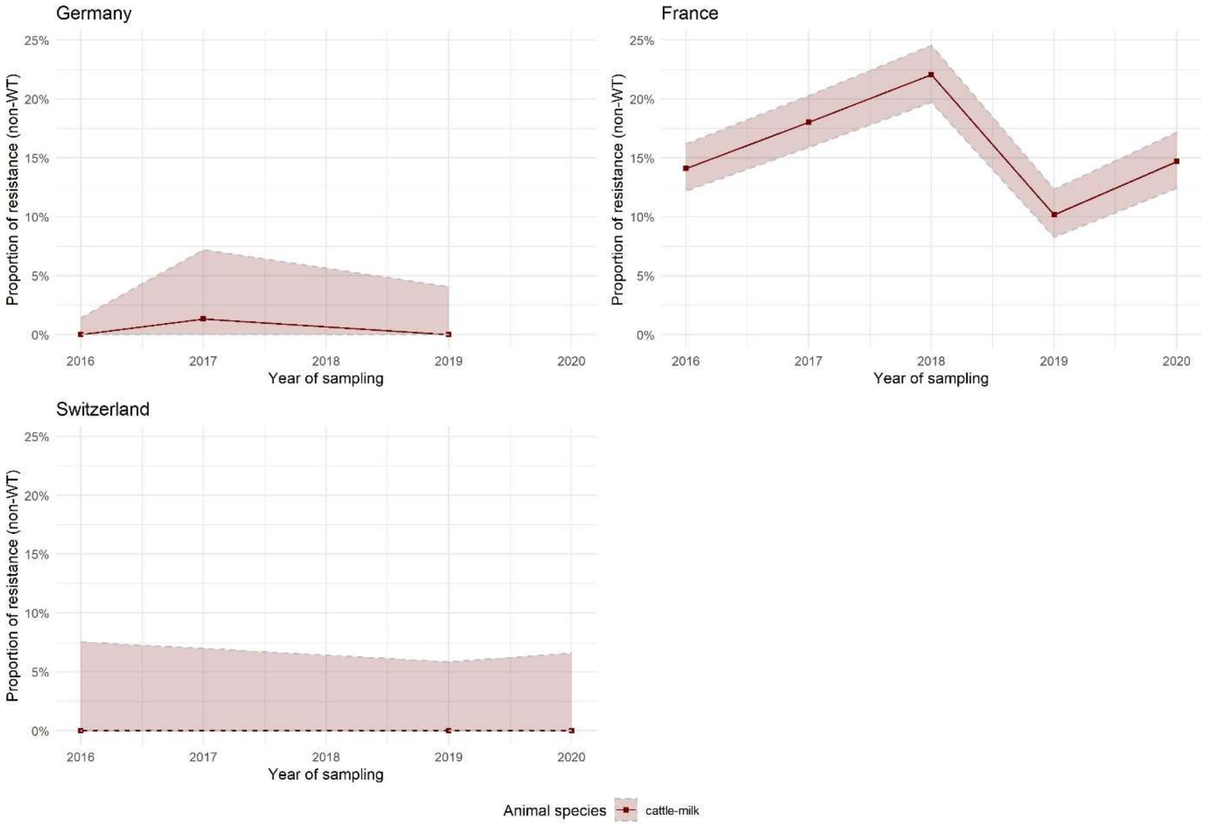

Figure S51. Trends of *Streptococcus uberis* resistance (non-wild-type) to sulfonamide-trimethoprim over 2016-2020

Only countries and animal species with sufficient data (at least 30 isolates per animal species and per year) are displayed on the figures.

Coloured areas around the curves represent 95% confidence intervals.
